# Transcription Factor 4 loss-of-function is associated with deficits in progenitor proliferation and cortical neuron content

**DOI:** 10.1038/s41467-022-29942-w

**Published:** 2022-05-02

**Authors:** Fabio Papes, Antonio P. Camargo, Janaina S. de Souza, Vinicius M. A. Carvalho, Ryan A. Szeto, Erin LaMontagne, José R. Teixeira, Simoni H. Avansini, Sandra M. Sánchez-Sánchez, Thiago S. Nakahara, Carolina N. Santo, Wei Wu, Hang Yao, Barbara M. P. Araújo, Paulo E. N. F. Velho, Gabriel G. Haddad, Alysson R. Muotri

**Affiliations:** 1 Department of Genetics, Evolution, Microbiology and Immunology, Institute of Biology, University of Campinas, Campinas, Sao Paulo 13083-862 Brazil; 2 Department of Pediatrics, School of Medicine, University of California San Diego, La Jolla, CA 92093 USA; 3 Center for Medicinal Chemistry, University of Campinas, Campinas, Sao Paulo 13083-886 Brazil; 4 Graduate Program in Genetics and Molecular Biology, Institute of Biology, University of Campinas, Campinas, Sao Paulo 13083-862 Brazil; 5 Lawrence Berkeley National Laboratory, Berkeley, CA 94720 USA; 6 School of Medical Sciences, University of Campinas, Campinas, Sao Paulo 13083-887 Brazil; 7 Department of Neurosciences, School of Medicine, University of California San Diego, La Jolla, CA 92093 USA; 8 Rady Children’s Hospital, San Diego, CA 92123 USA; 9 Department of Cellular & Molecular Medicine, School of Medicine, University of California San Diego, La Jolla, CA 92093 USA; 10 Kavli Institute for Brain and Mind, University of California San Diego, La Jolla, CA 92093 USA; 11 Center for Academic Research and Training in Anthropogeny (CARTA) and Archealization (ArchC), University of California San Diego, La Jolla, CA 92093 USA

**Keywords:** Autism spectrum disorders, Induced pluripotent stem cells

## Abstract

Transcription Factor 4 (*TCF4)* has been associated with autism, schizophrenia, and other neuropsychiatric disorders. However, how pathological *TCF4* mutations affect the human neural tissue is poorly understood. Here, we derive neural progenitor cells, neurons, and brain organoids from skin fibroblasts obtained from children with Pitt-Hopkins Syndrome carrying clinically relevant mutations in *TCF4*. We show that neural progenitors bearing these mutations have reduced proliferation and impaired capacity to differentiate into neurons. We identify a mechanism through which *TCF4* loss-of-function leads to decreased Wnt signaling and then to diminished expression of *SOX* genes, culminating in reduced progenitor proliferation in vitro. Moreover, we show reduced cortical neuron content and impaired electrical activity in the patient-derived organoids, phenotypes that were rescued after correction of *TCF4* expression or by pharmacological modulation of Wnt signaling. This work delineates pathological mechanisms in neural cells harboring *TCF4* mutations and provides a potential target for therapeutic strategies for genetic disorders associated with this gene.

## Abstract (web summary)

Transcription Factor 4 (TCF4) has been associated with autism and schizophrenia. Here, the authors demonstrate aberrant proliferation and differentiation in neural cells and organoids carrying mutations in TCF4.

## Introduction

Transcription Factor 4 (*TCF4*; OMIM 602272) encodes a helix-loop-helix transcription factor highly expressed during brain development^1–4^ and implicated in neural lineage commitment and neuronal function^5–10^. *TCF4* gene variants have been associated with neuropsychiatric diseases such as schizophrenia, bipolar disorder, post-traumatic stress disorder, and major depressive disorder^11–15^. Importantly, de novo heterozygous mutations in *TCF4* cause an autism spectrum disorder known as Pitt-Hopkins Syndrome (PTHS; MIM 610954)^16–19^. However, little is known about how alterations in *TCF4* lead to impaired neural tissue development and function. The unifactorial genetic nature of PTHS offers a unique opportunity to dissect the underlying pathological molecular mechanisms and characterize the cellular abnormalities resulting from *TCF4* loss-of-function.

Patients with PTHS carry private *TCF4* mutations^16–18,20,21^, which may be deletions, translocations, frameshift, nonsense, or missense changes^22^. Clinically, these individuals display profound cognitive impairment, motor delay, hypotonia, breathing abnormalities, typical autistic behaviors, constipation, and a distinctive facial *gestalt*^20,23^.

Some mouse lines with mutations in *Tcf4* display PTHS-like symptoms—including deficits in social interaction, associative memory, and sensorimotor gating^24,25^, as well as abnormal cortical development^26,27^, neuronal migration^28–30^, and oligodendrocyte differentiation^31,32^. However, mouse models carrying *Tcf4* mutations in the clinically relevant heterozygous state exhibit mild phenotypes only, without the severe symptoms observed in patients.

In this study, we generate neural progenitor cells (NPCs) and neurons from induced pluripotent stem cells (iPSCs) from patients with PTHS to analyze the diseased cellular phenotypes under relevant genomic context. Importantly, we also derive patterned brain organoids, which have been successfully used to model cellular pathology during early neurodevelopment in several disorders^33–36^. Our data show that PTHS organoids are aberrant in size and structure, containing a higher percentage of NPCs and fewer neurons. PTHS NPCs exhibit reduced proliferation and impaired ability to differentiate into neurons. Importantly, we identify reduced canonical Wnt/β-catenin signaling and expression of SOX transcription factors as dysregulated events that mechanistically result in the PTHS cellular abnormalities. We also pharmacologically manipulate Wnt signaling and genetically correct *TCF4* expression, which rescue the PTHS neural characteristics. Taken together, our data reveal novel cellular and molecular phenotypes in human cells with clinically relevant *TCF4* mutations and show that these aberrations are reversible, providing routes for therapeutic intervention in individuals carrying genetic diseases associated with this gene.

## Results

### PTHS cortical organoids exhibit aberrant size and morphology

To gain insight into the pathophysiology caused by mutations in *TCF4*, we generated iPSC lines via cellular reprogramming of skin fibroblasts from five patients with PTHS and corresponding parents of matching sex (Supplementary Table 1). The patients harbor mutations that either eliminate the *TCF4* gene, eliminate its essential DNA-binding domain, or impact one of its transcriptional activation domains (Supplementary Fig. 1a). We ensured that all iPSC clones express stem cell markers (Supplementary Fig. 1b) and carry no unwanted chromosomal abnormalities (Supplementary Fig. 1c). PTHS and control iPSC lines exhibit indistinct growth rate (Supplementary Fig. 1d) or general ability to derive NPCs and neurons in vitro (Supplementary Fig. 1e, f).

**Fig. 1:**
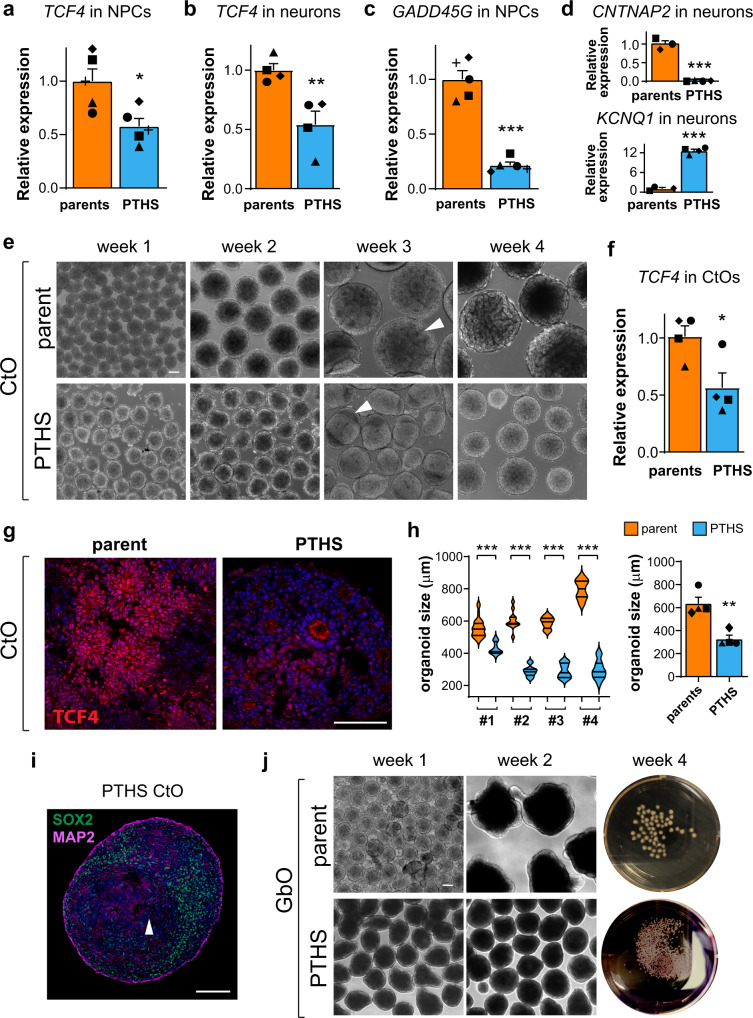
PTHS organoids display aberrant development. **a** Relative expression (RT-qPCR) of *TCF4* in parent and PTHS NPCs in 2D culture. *N* = 5 subjects per group (symbols; PTHS #1 to #5 in Supplementary Table 1). **b** Relative expression of *TCF4* in parent and PTHS neurons in 2D culture, after 3 months in neuronal medium. *N* = 4 subjects (symbols). **c** Relative expression of *GADD45G* in parent and PTHS NPCs in 2D culture. *N* = 5 subjects (symbols). **d** Relative expression of *CNTNAP2* (top) and *KCNQ1* (bottom) in parent and PTHS 2D neuronal cultures. *N* = 3 (control) or 4 (PTHS) subjects (symbols). **e** Bright-field microscopy images of parent and PTHS pallial cortical organoids (CtO) over 4 weeks of culture in vitro. Arrowhead in top row shows neural rosette. Arrowhead in bottom row indicates polarized phenotype. **f** Relative expression of *TCF4* in parent and PTHS CtOs evaluated at 4 weeks in vitro. *N* = 4 subjects (symbols). **g** Fluorescence images after TCF4 immunostaining (red) in parent and PTHS organoids at 4 weeks in vitro. **h** Left: CtO size distribution at 4 weeks in vitro, for 4 parent–child pairs (#1 to #4). Right: Mean CtO size at 4 weeks. *N* = 4 subjects per group (symbols). **i** Example of polarized phenotype (arrowhead) in PTHS CtO at 3 weeks in vitro after staining for SOX2 (green) and MAP2 (magenta). **j** Microscopy images of control and PTHS GABAergic-enriched organoids (GbOs) over 4 weeks of culture in vitro. Symbols in bar graphs indicate parent-patient identities: diamonds, pair #1; squares, pair #2; triangles, pair #3; circles, pair #4; crosses, pair #5. Colors in bar graphs and violin plots represent the parents (orange) or PTHS (blue) groups. Bar graphs represent mean + SEM. *p < 0.05, **p < 0.01, ***p < 0.001; two-sample ANOVA followed by Tukey-Kramer *post-hoc* test (left panel in **h**) or two-sample Welch’s *t* test assuming unequal variances (**a**–**d**, **f**, and right panel in **h**). The mean expression in the parental control group was normalized to 1 in (**a**–**d** and **f**). Blue staining is DAPI nuclear staining. Scale bars are 100 μm. See Supplementary Data 1 for statistical test results, including sample sizes, numbers of replicates, exact *p*-values, and effect sizes.

PTHS NPCs and neurons exhibit significantly reduced *TCF4* expression as compared to parental controls (Fig. 1a, b and Supplementary Fig. 1g, h; see Supplementary Data 1 for *p*-values and effect sizes). On average, PTHS lines display a half-way reduction in *TCF4* levels, in keeping with the presence of heterozygous whole-gene deletions or nonsense mutations in most lines (Supplementary Fig. 1a). In PTHS line #4 (circle symbol in Fig. 1a, b and Supplementary Fig. 1g, h), the reduction is less severe or statistically not significant, in keeping with its missense point mutation (Supplementary Fig. 1a), which is not expected to affect *TCF4* transcript abundance. Importantly, the expression of *GADD45G*, a direct transcriptional target of TCF4^37^, is strongly reduced in all PTHS NPCs (Fig. 1c). Likewise, PTHS neurons have altered expression of *CNTNAP2* and *KCNQ1* (Fig. 1d), previously shown to be directly positively and negatively regulated by TCF4, respectively^38,39^. Together, these data confirm that TCF4 function is impaired in our patient-derived cell lines.

Next, we generated brain cortical organoids (CtO)^35^ from the iPSC lines (Fig. 1e), followed by evaluation of aberrant phenotypes in PTHS organoids. PTHS CtOs exhibit reduced *TCF4* transcript (Fig. 1f) and TCF4 immunostaining (Fig. 1g and Supplementary Fig. 1i) levels. At 4 weeks in vitro, control CtOs display the expected spheroid-shaped organoid morphology and develop clearly visible rosette-like cellular aggregates (Fig. 1e). In marked contrast, PTHS CtOs are smaller (Fig. 1e, h), harbor fewer discernible rosettes (Fig. 1e), and some exhibit a polarized structure (Fig. 1e, i). These phenotypes are consistent across batches performed with different clones derived from the same patient (Supplementary Fig. 2a).

Because CtOs have few GABAergic inhibitory interneurons^35^, we additionally employed another derivation protocol to create brain organoids enriched with cells in the GABAergic lineage (GbOs) (Fig. 1j; see “Methods” for details; see GbO characterization below). Like in CtOs, PTHS GbOs are smaller (Fig. 1j) and display few or absent rosettes (Supplementary Fig. 2b).

Together, these results show that PTHS brain organoids have aberrant morphology and structure, suggesting that the development of PTHS neural tissue is abnormal.

### Altered content of progenitor cells and neurons in PTHS organoids

**Fig. 2:**
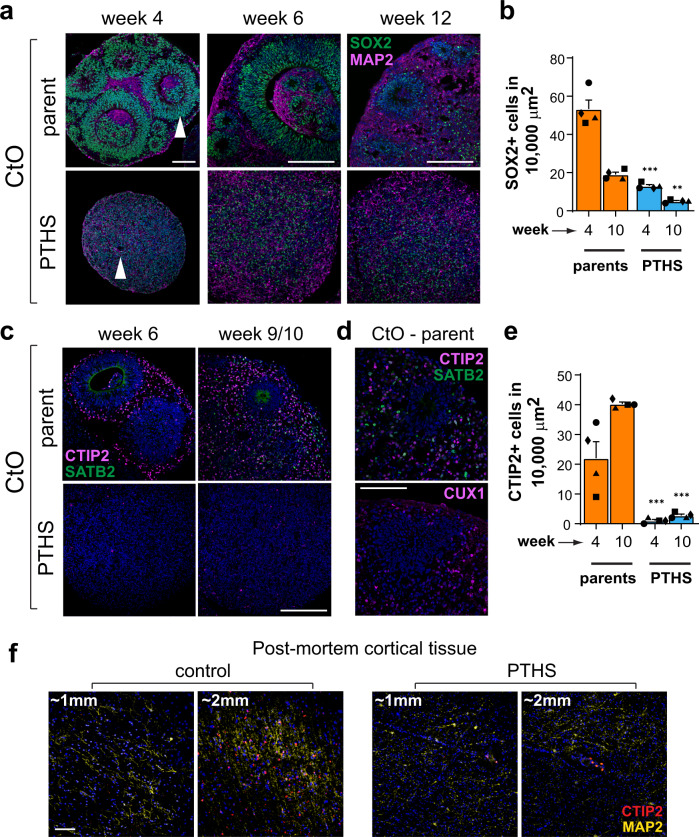
PTHS organoids display altered content of neural progenitors and cortical neurons. **a** Fluorescence microscopy images of parent and PTHS CtOs at different developmental stages after immunostaining for neural progenitor marker SOX2 and neuronal marker MAP2. Arrowheads indicate rosettes. **b** Quantification of SOX2+ cell density (cells in 100 × 100 μm area) at two stages of CtO development. *N* = 4 subjects (symbols), 3 batches per subject, 6 organoids per batch, 4 random 100 ×100 μm regions of interest (ROI) per organoid. See Supplementary Fig. 2c for quantification of SOX2+ cells in GbOs. **c** Immunostaining of parent and PTHS CtOs for cortical neuron subtype markers CTIP2 and SATB2 at two developmental stages. **d** Parent CtOs at 12 weeks in vitro after immunolabeling for CTIP2, SATB2, and CUX1. **e** Quantification of content of cortical neurons expressing CTIP2, at two stages of CtO development. *N* = 4 subjects (symbols), 3 batches per subject, 6 organoids per batch, 4 random ROIs per organoid. See Supplementary Fig. 2e for quantification of SATB2+ cells in CtOs. **f** Immunostaining for CTIP2 and MAP2 on post-mortem PTHS brain cortex tissue. Two ROIs are shown, at 2 mm cortical depth (equivalent to layer V in the control) and at 1 mm depth (equivalent to layer III). See Supplementary Fig. 2i for quantification of CTIP2+ cells at various cortical depths. Symbols in bar graphs indicate parent-patient identities: diamonds, pair #1; squares, pair #2; triangles, pair #3; circles, pair #4. Colors in bar graphs represent the parents (orange) or PTHS (blue) groups. Bar graphs represent mean + SEM. **p < 0.01, ***p < 0.001; one-way ANOVA followed by Tukey–Kramer’s HSD *post-hoc* test (**b** and **e**). Blue staining is DAPI nuclear staining. Scale bars are 100 μm. See Supplementary Data 1 for sample and effect sizes and exact *p*-values.

Smaller organoids may result from a range of altered cellular processes, such as decreased cell division or increased apoptosis, abnormal migration, or senescence. To identify which of these processes is defective in PTHS organoids, we analyzed the organization and contents of several key cellular subtypes. First, we performed immunostaining for neural progenitor marker SOX2 on CtOs and GbOs. At 4 weeks in vitro, control CtOs contain a large number of rosettes composed of neural progenitors surrounding a ventricle-like lumen (Fig. 2a), similar to the distribution of ventricular and sub-ventricular zone progenitors in the developing human brain^35^. As these progenitor-rich structures differentiate into several neuronal subtypes, the rosettes diminish in size (Fig. 2a). In contrast, PTHS organoids display very few rosette-like structures at 4 weeks in vitro and neural progenitors are dispersed and non-clustered (Fig. 2a; supporting controls in Supplementary Fig. 2a). In polarized PTHS CtOs, SOX2+ cells are concentrated on one side (Fig. 1i).

Importantly, we found that PTHS CtOs and GbOs have a significantly lower density of neural progenitors compared to control organoids (Fig. 2b and Supplementary Fig. 2b, c). The density of SOX2+ cells may not present a complete view of progenitor content, because cells in PTHS organoids are generally more dispersed, due to the absence of densely packed rosettes. Therefore, we additionally measured the percentages of SOX2+ cells over DAPI-stained nuclei, observing that, despite its lower density in PTHS organoids, neural progenitors represent a larger fraction of all cells in PTHS than in control CtOs (Supplementary Fig. 2d).

Immunostaining for MAP2 revealed that, in control organoids, neurons are distributed throughout the spheroid, particularly around and between rosettes, and neuronal content increases as development proceeds (Fig. 2a). Dissimilarly, PTHS CtOs and GbOs possess less evident MAP2 labeling, even at later stages of development (Fig. 2a and Supplementary Fig. 2b). Parental CtOs exhibit a typical pattern of cortical development, recapitulating the temporal progression of neuronal differentiation in the human cortex, in which deep-layer neurons (CTIP2+ cells) form first, followed by differentiation of superficial layer neurons (SATB2+ and CUX1+ cells) (Fig. 2c, d). In contrast, PTHS CtOs exhibit a severely reduced content of cortical neuron subtypes (Fig. 2c, e and Supplementary Fig. 2e). Additionally, PTHS CtOs display a reduction in staining for the excitatory neuron marker vesicular glutamate transporter family member 1 (vGLUT1)^33,35^ (Supplementary Fig. 2f, g). Similarly, PTHS GbOs have reduced staining for GABAergic neuron markers GAD65/67 (Supplementary Fig. 2f, g).

Importantly, we detected similarly decreased expression of *MAP2* and cortical neuron marker genes (Supplementary Fig. 2h) and fewer CTIP2+ neurons (Fig. 2f and Supplementary Fig. 2i) in a post-mortem PTHS cortex sample. Such deficit in CTIP2+ cells is probably not a consequence of mis-localized CTIP2+ neurons in the PTHS cortex, because fewer labeled cells are detected at several cortical depths (Supplementary Fig. 2i).

Together, these data strongly suggest that PTHS is characterized by severe deficits in cortical neuron content and indicate that patient-derived organoids closely match the neural phenotypes observed in vivo.

### Single-cell analyses confirm altered cellular content in PTHS

**Fig. 3:**
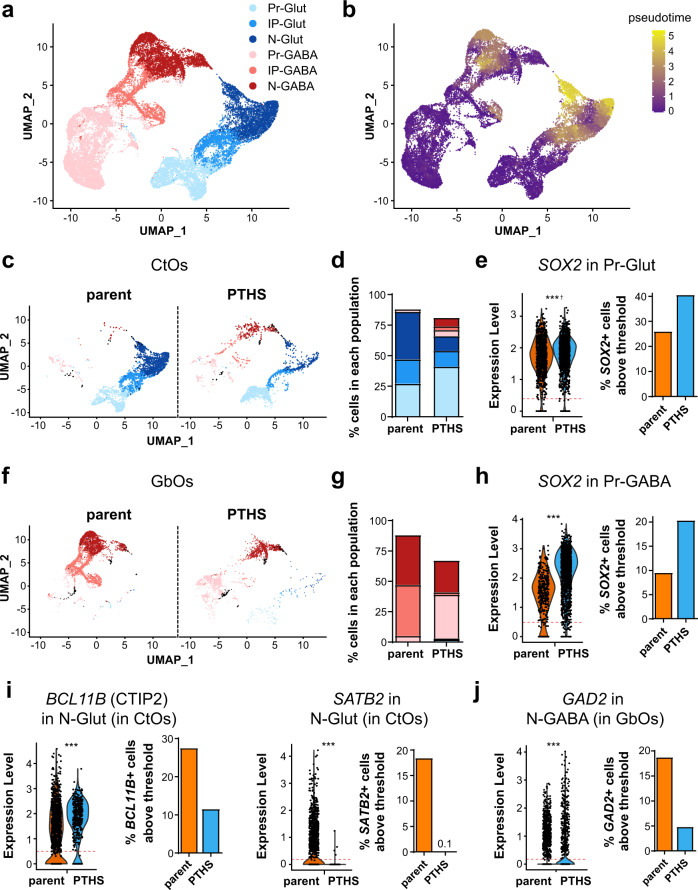
PTHS organoids have increased percentage of neural progenitors and decreased percentage of neurons. **a** Uniform Manifold Approximation and Projection (UMAP) bidimensional reduction of scRNA-Seq profiling of CtOs and GbOs at 8 weeks in vitro, integrating data from eight libraries: 3 libraries of parent #4 CtOs and 1 library each of PTHS #4 CtOs, parent #4 GbOs, PTHS #4 GbOs, CHIR99021-treated PTHS #4 CtOs, and CHIR99021-treated PTHS #4 GbOs, with pooled cells from 15 organoids per library. Color code represents 6 annotated subpopulations: Pr-Glut: neural progenitors in glutamatergic lineage; IP-Glut: intermediate progenitors in glutamatergic lineage; N-Glut: glutamatergic neurons; Pr-GABA: neural progenitors in inhibitory lineage; IP-GABA: intermediate progenitors in inhibitory lineage; N-GABA: neuronal population containing GABAergic interneurons. Minority subpopulations exist (‘Others’ in Supplementary Fig. 3) but were not the focus of our study. **b** Trajectory analysis indicating the existence of separate cell differentiation lineages in CtOs and GbOs. **c** Diversity of cell types (color-coded as in **a**) between parent and PTHS CtOs at 8 weeks in vitro. Black dots represent ‘Others’ group. **d** Percentages of cells in each CtO subpopulation (color code as in **a**). See Supplementary Table 2 for ‘Others’ and apoptotic subpopulations. **e** Left: Log-transformed expression abundance of *SOX2* (per cell basis) in the Pr-Glut subpopulation of CtOs; each dot represents a single cell. Right: Percentages of cells expressing *SOX2* above threshold (red line in violin plot, corresponding to 40% of overall average *SOX2* expression; see “Methods” for details). *N* = 959 (parent) and 1230 (PTHS) cells. **f** Diversity of cell types between parent and PTHS GbOs at 8 weeks in vitro. **g** Percentages of cells in each GbO subpopulation (color code as in **a**). **h** Left: Expression of *SOX2* in Pr-GABA cells of GbOs. Right: Percentages of *SOX2*+ cells above threshold (red line). *N* = 346 (parent) and 1376 (PTHS) cells. **i**, **j** Expression of *CTIP2* and *SATB2* (**i**) or *GAD2* (**j**) in N-Glut cells of CtOs (**i**) or N-GABA cells of GbOs (**j**). *N* = 1401 (parent) and 380 (PTHS) N-Glut (**i**), or *N* = 2661 (parent) and 988 (PTHS) N-GABA cells (**j**). Data are from scRNA-Seq analysis of parent #4 and PTHS #4 samples. ***p < 0.001; Kruskal–Wallis H test (left panels in **e**, **h**, **i**, and **j**). Cross symbol indicates that mean expression log2 fold change is lower than 0.5. Colors in bar graphs (except in **d** and **g**) and violin plots represent the parent (orange) or PTHS (blue) groups.

To corroborate the deficits in cellular diversity of PTHS brain organoids, we performed single-cell RNA sequencing (scRNA-Seq) on CtOs and GbOs at 8 weeks in vitro. We chose to analyze organoids derived from parent-patient pair #4, which display large differences in size and internal structure (Figs. 1h, 2b, e and Supplementary Data 1), thus increasing our chances of detecting cellular and molecular pathologies in the PTHS neural tissue. We focused our analyses on six annotated cellular subpopulations in CtOs and GbOs (Fig. 3a and Supplementary Fig. 3a–c), which compose distinct glutamatergic and GABAergic lineages, each with neural progenitors that progress through an intermediate progenitor stage towards the generation of neurons, as judged by differentiation trajectory analysis (Fig. 3b).

PTHS and control organoids do not contain cells expressing mesoderm or endoderm markers (Supplementary Figs. 2j, 3d) and, even though other minority neural populations are present (‘Others’ in Supplementary Fig. 3a, e–g), they were not studied because they could not be unequivocally assigned to the six populations chosen for analysis (Supplementary Fig. 3a; see “Methods” for details). As a control, we confirmed the reproducibility of our organoid experiments by determining that the cellular compositions of replicate scRNA-Seq libraries from independently derived parent CtOs are highly concordant (Supplementary Fig. 4a). We did not observe segregation of cells according to the sample of origin (batch effect) for these replicate libraries, and comparison between parent and PTHS organoids did not reveal segregation of the patient cells to a grossly distinct transcriptomic landscape (Supplementary Fig. 4b).

Analysis of the percentages of cells assigned to each subpopulation corroborated the existence of differences in cellular composition between parent and PTHS organoids (Supplementary Fig. 4c and Supplementary Table 2), including a higher percentage of progenitors in PTHS CtOs and GbOs (Fig. 3c–h). Astrocytes are rare and similarly infrequent in PTHS and control organoids at this stage (Supplementary Fig. 4d), ruling out differences in astroglia content as a potential cause for phenotypic abnormalities in PTHS organoids.

scRNA-Seq data also revealed lower percentages of cells in the N-Glut and N-GABA neuronal subpopulations in PTHS CtOs and GbOs, respectively, as compared to control organoids (Fig. 3c, d, f, g; Supplementary Table 2), in accordance with the scarcer neuronal populations detected by immunostaining (Fig. 2). Moreover, scRNA-Seq analyses showed that 41.6% of parent CtO cells express the glutamatergic markers *SLC17A7* (vGLUT1) or *SLC17A6* (vGLUT2), against 15.5% in PTHS CtOs, and the percentages of neurons expressing cortical markers *BCL11B* (CTIP2), *SATB2*, *TBR1*, and *CUX1* are lower in PTHS CtOs (Fig. 3i and Supplementary Fig. 4e, f). Likewise, 15.6% of cells in parent GbOs express *GAD1* (coding for GABAergic marker GAD65), against 6.4% in PTHS GbOs, and the percentage of neurons expressing *GAD2* (coding for GAD67) is smaller in PTHS GbOs as compared to controls (Fig. 3j). *GAD1/2*+ and *SLC17A6/7*+ neurons are rare in CtOs and GbOs, respectively, representing less than 4.0% of cells.

One possibility is that the reduced cortical neuron content in PTHS organoids is due to mis-patterning. To test this hypothesis, we conducted a comprehensive investigation of the expression of several neural lineage markers in CtOs and GbOs. Telencephalic markers, such as FOXG1, are expressed in most cells in CtOs, as judged from single cell data and immunostaining (Supplementary Fig. 4g–k), in keeping with the telencephalic origin of these cortical organoids. Importantly, the percentages of FOXG1+ cells are similar in PTHS and control CtOs (Supplementary Fig. 4h, i, k). Marker expression analysis in GbOs revealed that they contain a mixed population of telencephalic and non-telencephalic cells (Supplementary Fig. 4l). In fact, a fraction of all GbO cells is FOXG1+, and these occur at similar percentages in PTHS and parent organoids (Supplementary Fig. 4g–j, l). Metencephalic markers, such as *IRX3* and *TFAP2A* (found in some GABAergic lineage cells of non-telencephalic origin^40^), are expressed in a substantial fraction of all GbO cells (Supplementary Fig. 4g–i, l) and in *GAD1* + (GABAergic) GbO neurons (Supplementary Fig. 4l, m). Importantly, the percentages of GbO cells expressing these markers are similar in parent and PTHS organoids (Supplementary Fig. 4l, m), and expression of *TFAP2A* and its protein product AP2 are equivalent in both genotypes (Supplementary Fig. 4h, i, l). Finally, even though there is a small increase in the percentages of unassigned cell types (‘Others’ minority group) in PTHS organoids, their content is small and falls within the range of observed variability among replicates of control organoids (Supplementary Fig. 3f and Supplementary Table 2). In combination, these results show no evidence of mis-patterning in PTHS organoids.

Although scRNA-Seq data from additional parent-patient pairs are needed to expand these observations, our single cell transcriptomic results concur with the histological and molecular abnormalities found in PTHS organoids from all patient lines (Fig. 2). Together, both analyses suggest that PTHS organoids have proportionally more progenitors and fewer neurons and that the disease’s pathophysiology involves defects in progenitor proliferation and/or differentiation.

### PTHS neurons exhibit abnormal firing properties

To investigate neuronal function in PTHS, we analyzed organoids and neurons in 2D culture. First, we performed multi-electrode array (MEA) assays to measure neuronal activity in CtO organoids. We found that the firing rate is lower in PTHS CtOs (Supplementary Fig. 5a, b), concordant with the reduced expression of surrogate marker of neuronal activity *FOS* (Supplementary Fig. 5c, d) and with the lower numbers of c-Fos+ neurons (Supplementary Fig. 5e) in patient organoids.

Several potential explanations exist for the diminished activity in PTHS organoids, including changes in neuronal diversity. However, analysis of cellular composition in CtOs and GbOs did not reveal major changes in lineage commitment in PTHS organoids (Supplementary Fig. 4g). To further address this issue, we measured the impact of *TCF4* loss-of-function on neuronal diversity using 2D neuronal cultures. First, we confirmed that *TCF4* is expressed in control neurons in this type of culture (Supplementary Fig. 5f) and that PTHS neurons have a reduction in *TCF4* expression (Fig. 1b). Our iPSC-derived neuronal cultures are a mixture of different neuronal subtypes, including excitatory and inhibitory neurons, as previously reported^41^. Although we could not define the identity of neurons in these 2D cultures based on their electrophysiological properties in patch-clamp experiments (see below), deconvolution of RNA sequencing data revealed that PTHS samples possess fewer glutamatergic and GABAergic neurons than parental controls (Supplementary Fig. 5g), in agreement with the lower neuronal content observed in CtOs and GbOs (Fig. 3i, j). Importantly, we did not observe a preponderance of GABAergic inhibitory neurons in the PTHS organoid and 2D culture samples (Supplementary Fig. 5g and Supplementary Table 2) that could explain the lower electrical activity observed in PTHS organoids.

**Fig. 4:**
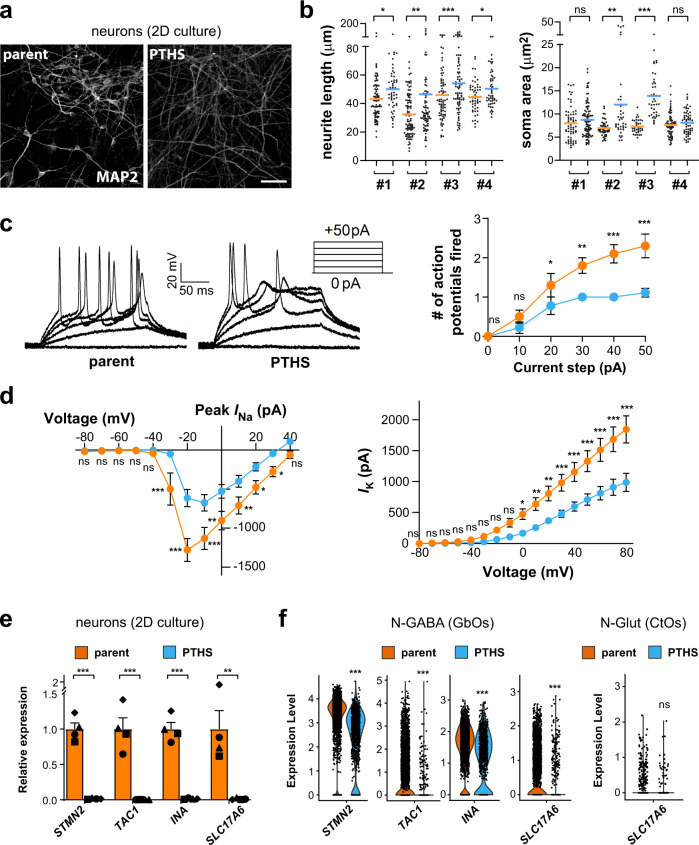
PTHS neurons exhibit abnormal electrophysiological properties and gene-expression program. **a** Neurons in 2D culture (3 months in neuronal medium) after MAP2 immunostaining (white). **b** Neurite length and soma area quantification in parent and PTHS neurons in 2D culture. *N* = 4 patients and respective controls (#1 to #4 pairs indicated below the graphs); 37-94 neurons per group (dots). Means are indicated by the colored lines. **c** Patch-clamp electrophysiological interrogation of parent and PTHS neurons, showing reduction in spike rate in PTHS neurons after 3 months in neuronal medium (right). Representative traces are shown on the left. *N* = 10 (parent) or 9 (PTHS) neurons from pair #4. **d** Sodium (left) and potassium (right) currents in parent (orange) and PTHS (blue) neurons. *N* = 10 (parent) or 9 (PTHS) neurons. **e** Relative expression (RT-qPCR) of selected neuronal genes in neurons in 2D culture (2 months in neuronal medium). *N* = 4 subjects per group (symbols). These genes were selected among the most differentially expressed in N-Glut and N-GABA neurons between parent and PTHS organoids (see Supplementary Data 3 for list of DE genes). **f** Left: Expression of the same genes shown in (**e**) in neurons of GbOs, at 8 weeks in vitro. *N* = 2661 (parent) and 988 (PTHS) cells. Right: For comparison with GbOs, the expression of *SLC17A6* (vGLUT2) in CtO neurons is also shown. *N* = 1401 (parent) and 380 (PTHS). Cells are from pair #4. Symbols in bar graphs indicate parent-patient identities: diamonds, pair #1; squares, pair #2; triangles, pair #3; circles, pair #4. Colors in the figure represent parent (orange) or PTHS (blue) groups. Bar graphs represent mean + SEM. n.s., not statistically significantly different, *p < 0.05, **p < 0.01, ***p < 0.001; one-way ANOVA with Geisser-Greenhouse correction for repeated measures followed by LSD *post-hoc* test (**b**), one-way ANOVA followed by HSD *post-hoc* test (**c** and **d**), two-sample Welch’s *t* test (**e**), or Kruskal–Wallis H test (**f**). Scale bar is 100 μm. See Supplementary Data 1 for sample and effect sizes and exact *p*-values.

A second possibility to explain the reduced firing rate in PTHS organoids is altered neuronal morphology, which, in combination with the PTHS organoids’ lower neuronal content (Figs. 2e, 3i and Supplementary Figs. 2e, 4e, f), might impair the way neurons project or establish synaptic connections. To assess if PTHS neurons are morphologically aberrant, we performed analysis of neuronal arborization architecture in neurons in 2D culture (Fig. 4a). We found that neuronal processes were longer in PTHS neurons and that the soma areas in some PTHS lines were larger than in parental controls (Fig. 4b).

Finally, a third hypothesis is that the reduced firing rate in PTHS organoids is caused by aberrant cellular-level electrophysiology. To assess this possibility, we employed patch-clamp analysis of neurons in 2D culture derived from the most significantly impaired patient line in the MEA recordings (Supplementary Fig. 5a). We found that PTHS neurons exhibit severely decreased intrinsic excitability (Fig. 4c), membrane capacitance, and sodium and potassium currents (Fig. 4d and Supplementary Fig. 5h, i), establishing that PTHS neurons display severe deficits in electrical properties at the cellular level.

The three hypotheses presented here are not mutually exclusive and further studies are needed to determine how the lowered neuronal content, aberrant morphological characteristics, or cellular-level electrophysiological alterations contribute to the diminished electrical activity in PTHS.

Next, we used RNA sequencing of PTHS and control neurons from 2D cultures to probe transcriptomic alterations that may shed light on the PTHS dysfunctional neuronal properties. Differential expression (DE) analysis revealed a range of mis-regulated genes, and those with highest fold-changes include some involved in neurogenesis, neuronal identity, differentiation, and regulation of neuronal excitability (Supplementary Fig. 5j, k and Supplementary Data 2), such as *STMN2* (stathmin 2), *TAC1* (tachykinin precursor 1), *CNTN2* (contactin 2), *INA* (internexin neuronal intermediate filament protein alpha), *ADCYAP1* (adenylate cyclase activating polypeptide 1), *SYT13* (synaptotagmin 13), and *SLC17A6* (vesicular glutamate transporter 2, vGLUT2) (Fig. 4e).

Many of these genes are also significantly downregulated in neurons of PTHS organoids, such as *STMN2*, *TAC1*, and *INA* (Fig. 4f and Supplementary Fig. 5d; see list of DE genes in organoid neurons in Supplementary Data 3). *SLC17A6* (vGLUT2)—which is expressed in glutamatergic cells of CtOs as well as in rare cells of GbOs (Supplementary Fig. 5l)—is also downregulated in PTHS organoids (Fig. 4f), and *SLC17A6*+ cells are fewer in both PTHS CtOs and GbOs (fewer dots in PTHS bars in Fig. 4f). Importantly, several genes coding for ion channels are significantly downregulated in PTHS neurons in 2D culture and in organoids (Supplementary Fig. 5d, m, n and Supplementary Data 2 and 3), including potassium channel genes *KCNQ2* and *KCNQ3*—related to *KCNQ1*, previously shown to dysregulate intrinsic excitability of mouse neurons after *Tcf4* knockdown^39^.

Together, these data indicate that PTHS neurons are aberrant in terms of morphology, physiology, and transcriptomic landscape, offering mechanistic insight into the PTHS neuronal intrinsic excitability defects and new opportunities for pharmacological therapeutic intervention.

### PTHS NPCs are less proliferative and senesce earlier

**Fig. 5:**
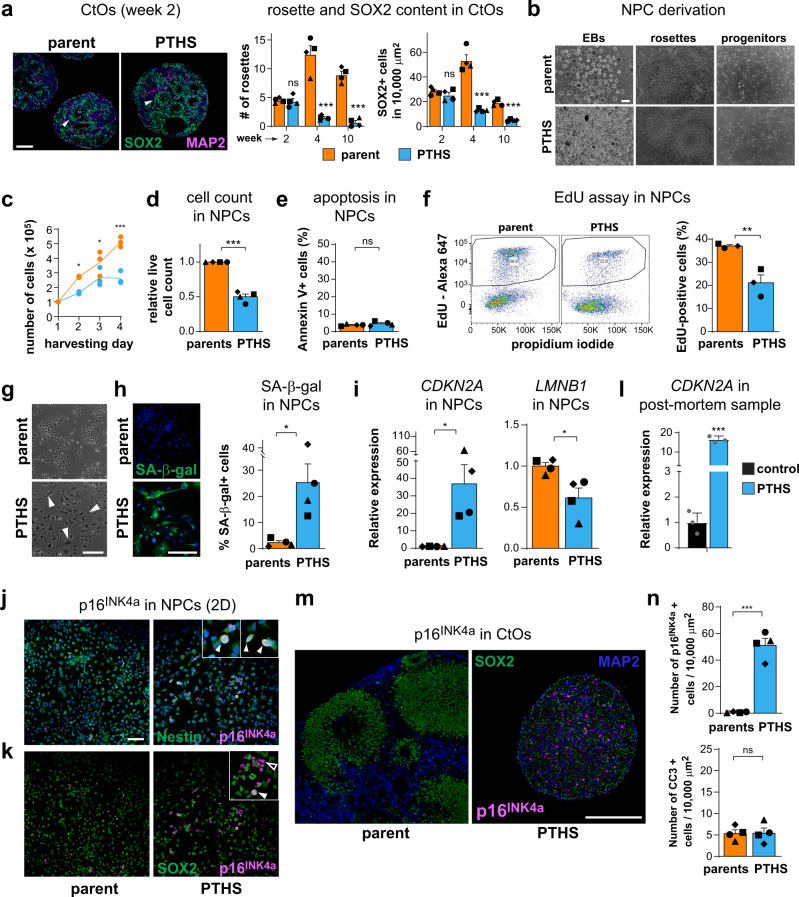
PTHS neural progenitors proliferate at a lower rate. **a** Left: CtOs at 2 weeks in vitro after staining for SOX2 and MAP2. Arrowheads mark neural rosettes. Middle: Number of rosettes in parent and PTHS CtOs at different organoid developmental stages in vitro. Right: Density of SOX2+ cells in CtOs. *N* = 4 parent–patient pairs (symbols). **b** Derivation of NPCs from iPSCs. **c** Example of growth curves for NPCs in 2D culture, for parent #4 (orange) and PTHS #4 (blue). *N* = 3 experiments (replicates) per time point. **d** Live cell counts for parent and PTHS NPCs in 2D culture. *N* = 4 pairs (symbols). **e** Quantification of Annexin V+ (apoptotic) NPCs in 2D culture. *N* = 4 pairs (symbols). **f** Left: Representative assessment of EdU+ (dividing) NPCs in 2D culture, for pair #1. Right: Percentage of EdU+ NPCs in parent and PTHS 2D cultures. *N* = 3 pairs (symbols). **g** Flat enlarged cells (arrowheads) in PTHS NPC 2D culture. **h** Left: Staining for senescence-associated β-galactosidase (SA-β-gal) activity (green) in NPCs in 2D culture (quantification on the right). *N* = 4 pairs (symbols). **i** Relative expression of *CDKN2A* (left) and *LMNB1* (right) in NPCs in 2D culture. *N* = 4 pairs (symbols). **j** Immunostaining of parent and PTHS NPCs for Nestin (green) and p16^INK4a^ (magenta; colocalization in insets). **k** Immunostaining of parent and PTHS NPCs for SOX2 and p16^INK4a^ (filled arrowhead: colocalization; open arrowhead: absence of co-staining). **l** Relative expression of *CDKN2A* in post-mortem PTHS brain cortex sample (PTHS #6). *N* = 3 replicates per group. **m** Immunostaining of parent and PTHS CtOs for SOX2 (green), MAP2 (blue), and senescence marker p16^INK4a^ (magenta), at 6 weeks in vitro. **n** Quantification of p16^INK4a^+ (top) and apoptotic (Cleave Caspase 3, CC3+; bottom) cells in CtOs at 6 weeks in vitro. *N* = 4 pairs (symbols). Symbols in bar graphs indicate parent-patient identities: diamonds, pair #1; squares, pair #2; triangles, pair #3; circles, pair #4; gray dots, post-mortem samples. Colors in bar and line graphs represent parents (orange), PTHS (blue), or control post-mortem sample (black) groups. Bar graphs represent mean + SEM. n.s. = not significant. *p < 0.05, **p < 0.01, ***p < 0.001; one-way ANOVA (**c**) or two-sample Welch’s *t* test in remaining comparisons. DAPI nuclear staining in blue (except in **m**). Scale bars are 100 μm. See Supplementary Data 1 for sample and effect sizes and exact *p*-values.

The fewer neural rosettes and abnormal progenitor content in PTHS organoids (Figs. 1–3) may result from abnormal neural induction, reduced progenitor proliferation, or impaired differentiation. To assess these possibilities, we first counted the numbers of rosettes at different organoid developmental stages (Fig. 5a). Even though rosette numbers and SOX2+ progenitor density are evidently different in PTHS organoids at weeks 4 and 10, these parameters are similar between PTHS and parent organoids at week 2, right after the neural induction phase (Fig. 5a). These results, together with the absence of cells expressing non-neural markers (Supplementary Fig. 3d, g), strongly suggest that neural induction is normal in PTHS organoids and that rosettes dwindle at later stages during organoid maturation.

To parse out between the remaining two possibilities—poor progenitor proliferation and impaired differentiation—we analyzed NPCs in 2D culture (Fig. 5b; Supplementary Fig. 6a). Parental NPCs indeed express TCF4 (Supplementary Fig. 6b–d), and expression of both *TCF4* and its target *GADD45G* are reduced in all PTHS NPC lines (Fig. 1a, c), confirming that TCF4 function is impaired. Importantly, we observed that PTHS NPCs grow significantly slower than control lines (Fig. 5c, d). This difference does not arise from increased apoptosis, because Annexin V+ cells are equally infrequent in PTHS and parental NPC cultures (Fig. 5e). Instead, the proliferative capacity of PTHS NPCs was shown to be impaired (Fig. 5f), as judged by flow-cytometry determination of the percentages of dividing cells labeled with thymine nucleoside analog 5-ethynyl-2′-deoxyuridine (EdU).

We also observed that PTHS NPCs frequently assume an atypical enlarged, flat morphology (Fig. 5g). The combination of aberrant morphology and diminished proliferative activity led us to hypothesize that PTHS neural progenitors are undergoing precocious replicative senescence, a process characterized by cell cycle arrest and subsequent halting of proliferation^42,43^. In fact, we observed three hallmarks indicative of replicative senescence in PTHS NPCs in addition to larger cell size: heightened β-galactosidase activity (SA-β-gal; Fig. 5h), reduced expression of the nuclear lamina protein lamin B (*LMNB1*; Fig. 5i), and markedly increased expression of cyclin-dependent kinase inhibitor genes *CDKN2A* (Fig. 5i) and *CDKN1A* (Supplementary Data 2)—whose expression causes cell division to stall, acting as replicative senescence markers^42^. Interestingly, these characteristics intensify with increasing number of passages in PTHS NPCs (Supplementary Fig. 6e, f and Supplementary Data 2). We also determined that PTHS NPCs expressing p16^INK4a^ (*CDKN2A* gene product) are Nestin+ (Fig. 5j), most are SOX2+ (Fig. 5k) and are negative for mesoderm marker Brachyury and endoderm marker SOX17 (Supplementary Fig. 6g), indicating that they are senescent NPCs, not mis-differentiated cells.

Strikingly, the expression of senescence markers is also up-regulated in the PTHS post-mortem cortex sample (Fig. 5l). Concordantly, PTHS CtOs contain many neural lineage cells expressing p16^INK4a^ (Fig. 5m, n and Supplementary Fig. 6h) and these are not apoptotic cells, which are similarly infrequent in both PTHS and control organoids (Fig. 5n). Importantly, shRNA-mediated *TCF4* knockdown in control NPCs led to decreased proliferation (Supplementary Fig. 6i) and higher expression of *CDKN2A* (Supplementary Fig. 6j), further strengthening the link between the reduction in *TCF4* expression and increased senescence and decreased proliferation in patient-derived NPCs.

Together, these data indicate that the PTHS cellular pathology involves decreased proliferation and augmented senescence of NPCs.

### Wnt signaling correction rescues aberrant PTHS phenotypes

To gain mechanistic insights into the aberrant proliferative activity of PTHS progenitors, we performed RNA sequencing of NPCs in 2D culture from 4 parent–child pairs, followed by unbiased investigation of differentially expressed (DE) genes between PTHS and control cells (Supplementary Fig. 7a and Supplementary Data 2).

Gene set enrichment analysis on the up-regulated genes in PTHS NPCs indicated enrichment for genes involved in cellular senescence or tissue architecture (Supplementary Fig. 7b). One example of the latter is HOP Homeobox (*HOPX*), expressed in outer radial glia (oRG) cells in the developing human brain^44^ and in some astrocytic precursors and astrocytes^45^. Its higher expression in PTHS NPC lines from several patients (Supplementary Fig. 7c, d) may indicate that a higher proportion of oRG-like and/or astrocytic cells are present in these 2D cultures. We investigated whether the PTHS neural tissue displays a higher content of *HOPX*+ cells and found that the percentages of these cells are lower in PTHS CtOs and higher in PTHS GbOs, as compared to the respective control organoids (Supplementary Fig. 7e), phenotypes that were confirmed by HOPX immunostaining (Supplementary Fig. 7f). Interestingly, only a fraction of *HOPX*+ cells express the astrocytic marker *S100B* in both CtOs and GbOs (Supplementary Fig. 7e), which suggests that the changes in the percentages of HOPX+ cells in PTHS organoids is, at least in part, due to changes in oRG content. Although these findings and their significance to PTHS pathophysiology require further investigation, it is noteworthy that *HOPX* expression is also higher in the post-mortem PTHS cortex (Supplementary Fig. 7g).

**Fig. 6:**
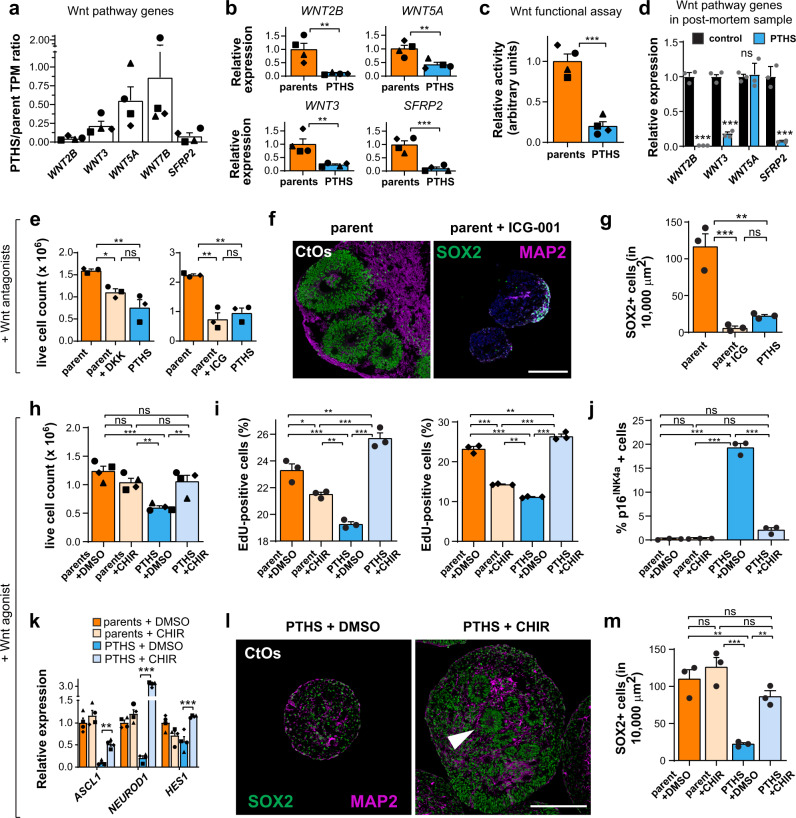
Manipulation of Wnt signaling rescues proliferation of PTHS neural progenitors. **a** Ratio of TPM expression abundances for selected Wnt genes between parent and PTHS NPCs in 2D culture. *N* = 4 parent–child pairs (symbols). **b** Relative expression of Wnt genes in NPCs. *N* = 4 pairs (symbols). **c** Reduced Wnt signaling activity in PTHS NPCs in 2D culture (TOP-Flash assay). *N* = 4 pairs (symbols). **d** Relative expression of selected Wnt genes in post-mortem PTHS cortex sample (PTHS #6). *N* = 3 replicates per group. **e** Treatment of control NPCs with Wnt pathway antagonists DKK-1 and ICG-001 (yellow bars) phenocopies proliferation deficit in PTHS progenitors. *N* = 3 pairs (symbols). **f** Parent CtOs at 4 weeks in vitro after treatment with ICG-001, stained for SOX2 and MAP2. **g** ICG-001 treatment of CtOs phenocopies low neural progenitor content (SOX2) of PTHS organoids. *N* = 3 replicates (circles) with pair #4 cells. **h** Live cell count after treatment of NPCs in 2D culture with Wnt pathway agonist CHIR99021. *N* = 4 pairs (symbols). **i** EdU assay in NPCs treated with CHIR99021. Left graph represents data for pair #4, and right graph shows data for pair #1. *N* = 3 replicates. **j** Quantification of p16^INK4a^+ (senescent) cells in NPCs in 2D culture treated with CHIR99021. Data shown are for pair #4 (see graph in Supplementary Fig. 8g for pair #1). *N* = 3 replicates. **k** CHIR99021 rescues expression of proliferation genes in treated PTHS NPCs in 2D culture. *N* = 4 pairs (symbols). See pairwise comparisons in Supplementary Data 1. **l** PTHS CtOs at 4 weeks in vitro after treatment with CHIR99021, showing marked increase in abundance of NPCs (SOX2; green) and neurons (MAP2) as well as reappearance of neural rosettes (arrowhead). **m** Quantification of SOX2+ cells after treatment of PTHS CtOs with CHIR99021. *N* = 3 replicates (circles) with PTHS #4 cells. Symbols in bar graphs indicate parent-patient identities: diamonds, pair #1; squares, pair #2; triangles, pair #3; circles, pair #4; gray dots, post-mortem samples. Colors in bar graphs represent parents (orange), pharmacologically treated parents (yellow), PTHS (blue), pharmacologically treated PTHS (light blue), or control post-mortem sample (black) groups. Bar graphs represent mean + SEM. n.s., not significant; *p < 0.05, **p < 0.01, ***p < 0.001; two-sample Welch’s *t* test (in **b**–**d**) or one-way ANOVA followed by Tukey’s HSD *post-hoc* test in remaining panels. Scale bars are 100 μm. See Supplementary Data 1 for sample and effect sizes and exact *p*-values.

Gene set enrichment analysis on the downregulated genes in PTHS NPCs revealed alterations mostly in the expression of Wnt signaling pathway and cadherin genes (Supplementary Fig. 7h). Because the Wnt pathway has been linked to progenitor proliferation in many tissues^46^, we raised the hypothesis that abnormal Wnt activity may be causally implicated with the lower NPC proliferation rates observed in PTHS cells. In fact, we confirmed that several Wnt pathway genes have lower mean expression in PTHS NPCs (Fig. 6a, b) and that *WNT2B, WNT3*, and *SFRP2* are statistically significantly downregulated in the PTHS lines (Supplementary Fig. 8a, b). Functional assessment of Wnt signaling using a luciferase reporter indicated prominent reduction in canonical Wnt/β-catenin activity in the NPCs in 2D culture (Fig. 6c). Importantly, expression of several Wnt pathway genes is markedly downregulated in the post-mortem PTHS cortex sample (Fig. 6d), and *WNT5A*, *SFRP1*, and *APC* (a key Wnt signaling regulator) are downregulated in neural progenitors of CtOs and GbOs (Supplementary Fig. 8c and Supplementary Data 3).

Treatment of control NPCs in 2D culture with Wnt signaling antagonists DKK-1 and ICG-001 phenocopied the reduction in PTHS progenitor proliferation (Fig. 6e) and the increase in *CDKN2A* expression (Supplementary Fig. 8d). Treatment of control CtOs with ICG-001, a diffusible small molecule that can easily penetrate the organoid, led to a polarized structure and marked reduction in organoid size (Fig. 6f and Supplementary Fig. 8e), as well as fewer SOX2+ cells (Fig. 6g). As a reverse approach, we treated PTHS NPCs in 2D culture with the Wnt signaling agonist CHIR99021. First, we confirmed that Wnt signaling was increased in the treated cells (Supplementary Fig. 8f). Treatment with CHIR99021 rescued the proliferation rates of PTHS NPCs (Fig. 6h, i), decreased the percentage of senescent (p16^INK4a^+) cells (Fig. 6j and Supplementary Fig. 8g), and increased the expression of pro-proliferative gene *HES1* and pro-neural genes *ASCL1* and *NEUROD1* (Fig. 6k). Importantly, treatment of PTHS CtOs with CHIR99021 caused a significant increase in organoid size (Supplementary Fig. 8h) and progenitor content (Fig. 6l, m and Supplementary Fig. 9a, b), with the reappearance of conspicuous neural rosettes.

CHIR-treated PTHS CtOs possess an increased percentage of GABAergic lineage progenitors (Supplementary Fig. 9a) and higher expression of GABAergic markers (Supplementary Fig. 9c), raising the possibility that a partial cellular fate change from a cortico-pallial to a non-pallial trajectory might have caused the reversal in proliferation and senescence phenotypes observed in PTHS cells after Wnt signaling activation. However, CHIR treatment also results in increased expression of GABAergic markers (Supplementary Fig. 9c) without increasing size (Supplementary Fig. 8h) or progenitor content (Fig. 6m) in parent organoids, nor does it increase proliferation in parent NPCs in 2D culture (Fig. 6h, i). These results suggest that fate restriction does not cause the phenotypic corrections after Wnt signaling activation in PTHS NPCs, although further experiments are needed to confirm this hypothesis.

It is possible that the phenotypic correction after CHIR treatment is due to the increased expression of *TCF4* in CHIR-treated PTHS NPCs in 2D culture (Supplementary Fig. 9d), an effect also reported in other cell types^47^. However, neural progenitors in the context of the organoid’s 3D structure do not exhibit an increase in *TCF4* expression levels after CHIR treatment (Supplementary Fig. 9e). Moreover, TCF4 protein levels are not increased after CHIR treatment in PTHS NPCs in 2D culture (Supplementary Fig. 9f, g). These data allow us to conclude that the rescue of proliferation defect in PTHS organoids was due to corrected Wnt signaling activation downstream of *TCF4*.

Together, our data reveal the mechanistic involvement of dysregulated Wnt signaling in the PTHS NPC proliferation defects and show that the aberrant PTHS phenotypes can be pharmacologically corrected.

### Investigation of β-catenin and cadherin dysregulation in PTHS

Since β-catenin is a key component of the Wnt pathway and an important regulator of epithelial cell adhesion and integrity^46^, a plausible hypothesis is that the diminished Wnt signaling in PTHS progenitors results in dysregulated β-catenin expression, leading to dismantling of rosettes and failure to organize the neuroepithelial architecture. In fact, even though the expression of β-catenin remains unchanged in PTHS NPCs and PTHS CtO progenitors (Supplementary Fig. 9h), β-catenin localization is disorganized in PTHS organoids (Supplementary Fig. 9i), strengthening the possibility that Wnt signaling downregulation leads to neuroepithelial integrity defects during neurodevelopment in PTHS.

We also sought to determine whether the expression of cadherins or protocadherins is altered in PTHS cells, because this functional category is downregulated in PTHS NPCs (Supplementary Fig. 7h). Closer examination revealed that most DE genes in this category are Wnt pathway components (Supplementary Data 2), except *CDH23* and *PCDH15. CDH23* was discarded as a potential mechanistic candidate because its expression is negligible in NPCs (Supplementary Fig. 9j). *PCDH15* was found to be significantly downregulated in PTHS NPCs, however CHIR99021 treatment further reduces its expression (Supplementary Fig. 9j) in the same conditions in which the cellular phenotypes are corrected (Fig. 6h–j), ruling out diminished *PCDH15* as a plausible cause for the abnormal phenotypes in PTHS NPCs.

### Altered *SOX* expression changes NPC proliferation and differentiation

Next, we sought to define mechanistic players downstream of TCF4 and the Wnt pathway that could control NPC proliferation and differentiation. Because an interplay has been described between SRY-related HMG-box (SOX) proteins and Wnt signaling and due to their known roles in cell proliferation/differentiation^48^, we investigated the expression of *SOX* genes in PTHS NPCs. We focused on the *SOXB* and *SOXC* subfamilies because *SOXB* members (*SOX1*, *SOX2*, *SOX3*, and *SOX21)* have been traditionally regarded as regulators of cell proliferation^49^, while *SOXC* members (*SOX4*, *SOX11*, and *SOX12*) are pro-differentiation factors^50^.

**Fig. 7:**
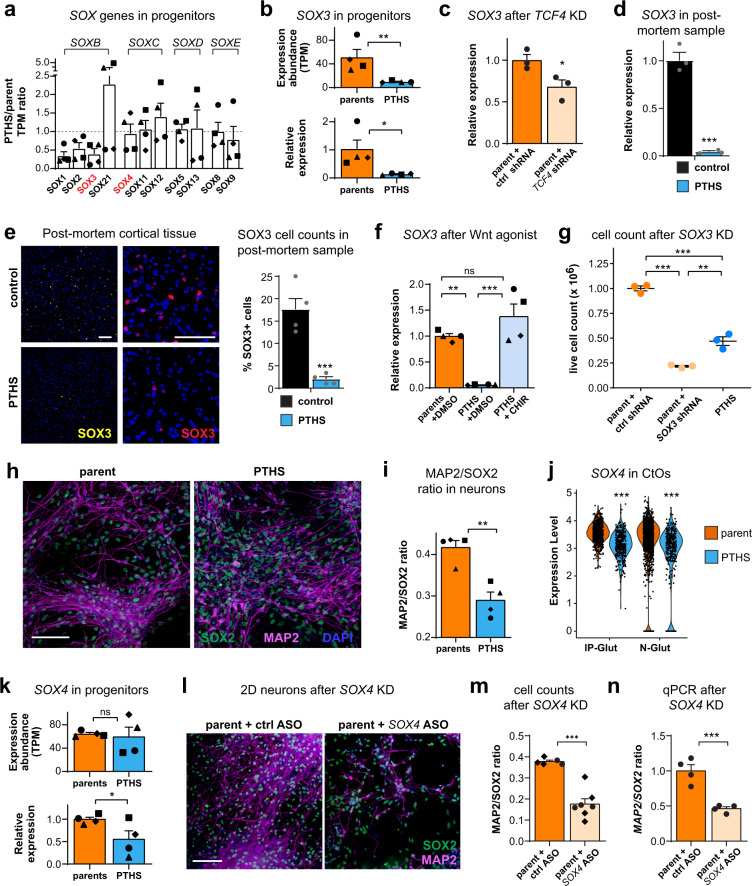
Aberrant expression of *SOX* genes in PTHS cells and organoids. **a** Ratio of TPM abundances for *SOX* genes in NPCs in 2D culture. *N* = 4 pairs (symbols). *SOX* gene subfamilies are shown above. **b** Top: *SOX3* TPM expression in NPCs. Bottom: *SOX3* relative expression (RT-qPCR). *N* = 4 pairs (symbols). **c** *SOX3* is downregulated after *TCF4* knockdown in NPCs in 2D culture. *N* = 3 replicates (circles), with parent #4 cells. **d** *SOX3* relative expression in post-mortem PTHS cortex sample (PTHS #6). *N* = 3 replicates per group. **e** Left: Immunostaining for SOX3 in post-mortem PTHS cortex sample (higher magnification images on the right, showing colocalization with DAPI). Right: Quantification of SOX3+ cells in post-mortem PTHS sample. *N* = 4 sections per group. **f** Treatment of PTHS NPCs in 2D culture with CHIR99021 rescues *SOX3* expression. *N* = 4 pairs (symbols). **g** *SOX3* knockdown reduces NPC proliferation in 2D culture. *N* = 3 replicates (circles), with pair #4 cells. **h** Immunostaining for SOX2 and MAP2 in neurons in 2D culture (2 months in neuronal medium). **i** Ratio between neurons (MAP2+) and NPCs (SOX2+) in neuronal 2D cultures. *N* = 4 pairs (symbols). **j** *SOX4* expression is reduced in IP-Glut and N-Glut cells of PTHS CtOs at 8 weeks in vitro. *N* = 717 (parent) and 382 (PTHS) IP-Glut cells, or 1401 (parent) and 380 (PTHS) N-Glut neurons from pair #4. **k** *SOX4* expression is reduced in PTHS NPCs in 2D culture. Top: TPM expression. Bottom: Relative expression (RT-qPCR). *N* = 4 pairs (symbols). **l** Immunostaining for SOX2 and MAP2 in differentiating neuronal 2D cultures after *SOX4* knockdown (2 months in neuronal medium). **m** Ratio between MAP2+ and SOX2+ after *SOX4* knockdown in differentiating neuronal 2D cultures. *N* = 6 (parent) or 7 (PTHS) replicates, with cells from pairs #1 and #4 (symbols). **n** Ratio between *MAP2* and *SOX2* gene-expression levels after *SOX4* knockdown in differentiating neuronal 2D cultures. *N* = 4 replicates, with cells from pair #4. Symbols in bar graphs indicate parent-patient identities: diamonds, pair #1; squares, pair #2; triangles, pair #3; circles, pair #4; gray dots, post-mortem samples. Colors in bar graphs, dot or violin plots represent parents (orange), genetically manipulated parents (yellow), PTHS (blue), pharmacologically treated PTHS (light blue), or control post-mortem sample (black) groups. Bar graphs represent mean + SEM. n.s., not significant; *p < 0.05; **p < 0.01; ***p < 0.001; one-way ANOVA (**f, g**), Kruskal–Wallis H test (**j**), or two-sample Welch’s *t* test in remaining panels. Scale bars are 100 μm. DAPI nuclear staining in blue. See Supplementary Data 1 for sample and effect sizes and exact *p*-values.

First, we found that *SOXB* genes *SOX1* and *SOX3* are significantly downregulated in all patient-derived NPCs in 2D culture (Fig. 7a and Supplementary Fig. 10a). In fact, these genes were found to be predominantly expressed in progenitors and intermediate progenitors of CtOs and GbOs (Supplementary Figs. 3b, 10b–d). However, *SOX1* was discarded as a candidate because it is not substantially expressed in organoids (Supplementary Fig. 10b and Supplementary Data 2). *SOX21* was discarded because it is not DE in most parent-patient NPC line pairs (Supplementary Fig. 10a). Even though *SOX2* is the most extensively investigated pro-proliferation *SOX* gene, it was also not significantly DE in PTHS NPCs in 2D culture (Supplementary Fig. 10a).

Therefore, we focused our experimental efforts on *SOX3*, whose expression is decreased in all PTHS lines (Fig. 7a, b). *SOX3* was found to be functionally downstream of TCF4, since shRNA-mediated *TCF4* knockdown in control NPCs led to a decrease in *SOX3* expression (Fig. 7c), an effect not observed for *SOX2* (Supplementary Fig. 10e). Importantly, the post-mortem PTHS cortex sample exhibits decreased expression of *SOX3* (Fig. 7d) and number of SOX3+ cells (Fig. 7e). We also concluded that *SOX3*, but not *SOX2*, is downstream of the Wnt signaling pathway, because treatment of PTHS progenitors with CHIR99021 increases *SOX3* expression, an effect not seen for *SOX2* (Fig. 7f and Supplementary Fig. 10f). Importantly, we determined that shRNA-mediated *SOX3* knockdown in control NPCs led to a reduction in cell counts (Fig. 7g and Supplementary Fig. 10g), increase in the expression of senescence marker *CDKN2A* (Supplementary Fig. 10h), and decrease in the expression of pro-proliferative gene *HES1* and pro-neural gene *ASCL1* (Supplementary Fig. 10h), matching the phenotypes observed in PTHS NPCs. Interestingly, transfection-mediated transient *SOX3* over-expression did not rescue the proliferative defect in PTHS NPCs (Supplementary Fig. 10i, j), probably due to lack of sustained *SOX3* over-expression during the NPC proliferation assay.

Next, we investigated whether PTHS progenitors harbor altered expression of *SOXC* genes, known to regulate neuronal differentiation^50^. The possibility that differentiation is impaired in PTHS is supported by the lower neuron-to-progenitor ratio in differentiating PTHS 2D cultures (Fig. 7h, i and Supplementary Fig. 11a). In accordance, the distribution of cells along the differentiation trajectory in PTHS organoids is skewed toward early time points, as compared with control organoids (Supplementary Fig. 11b). Moreover, intermediate progenitors are scarcer in PTHS CtOs and GbOs (Supplementary Fig. 11c and Supplementary Table 2), and the percentage of cells expressing intermediate progenitor marker *POU3F2* (BRN2) is lower in PTHS organoids (Supplementary Fig. 11d), as are the expression levels of *POU3F2* and pro-neural *NEUROD6* and *ID* genes in neurons of PTHS organoids (Supplementary Fig. 5d).

Indeed, we found that *SOXC* genes *SOX4*, *SOX11*, and *SOX12* are expressed in intermediate progenitors and neurons of CtOs and GbOs (Fig. 7j and Supplementary Fig. 11e–g). *SOX12* was not pursued further because it is not DE in most parent-patient pairs of NPCs in 2D culture (Supplementary Fig. 10a) and it is not DE in neural progenitors, intermediate progenitors, and neurons of CtOs (Supplementary Figs. 5d, 8c and 11g and Supplementary Data 3; see GO analysis of DE genes in intermediate progenitors in Supplementary Fig. 11h). We focused on *SOX4* because it was shown to be involved in intermediate progenitor-to-neuron differentiation^50^.

*SOX4* expression is lower in three of the PTHS NPC lines in 2D culture (Fig. 7k), in progenitors, intermediate progenitors and neurons of PTHS CtOs (Fig. 7j and Supplementary Figs. 5d, 8c and 11g), and in GABAergic neurons of PTHS GbOs (Supplementary Figs. 5d and 11e and Supplementary Data 3). Moreover, *SOX4* expression is significantly different along the differentiation trajectory between parent and PTHS CtOs (Supplementary Fig. 11i). To test the involvement of SOX4 in differentiation pathology, we performed locked nucleic acid antisense oligonucleotide (LNA ASO)-mediated *SOX4* knockdown in differentiating neuronal 2D cultures from two parent lines. Both cell count and transcriptomic analyses revealed that the differentiation rate (MAP2-to-SOX2 ratio) is reduced after *SOX4* knockdown (Fig. 7l–n), mimicking the aberrant phenotypes of PTHS neuronal cultures (Fig. 7i).

Based on these observations, we put forth a model according to which *TCF4* loss-of-function results in Wnt downregulation and, consequently, in reduced *SOX3* expression, leading to diminished proliferation and increased cellular senescence. In parallel, we hypothesize that reduced *SOX4* expression leads to impaired differentiation in the PTHS neural tissue.

### Reversal of PTHS phenotypes via correction of *TCF4* expression

**Fig. 8:**
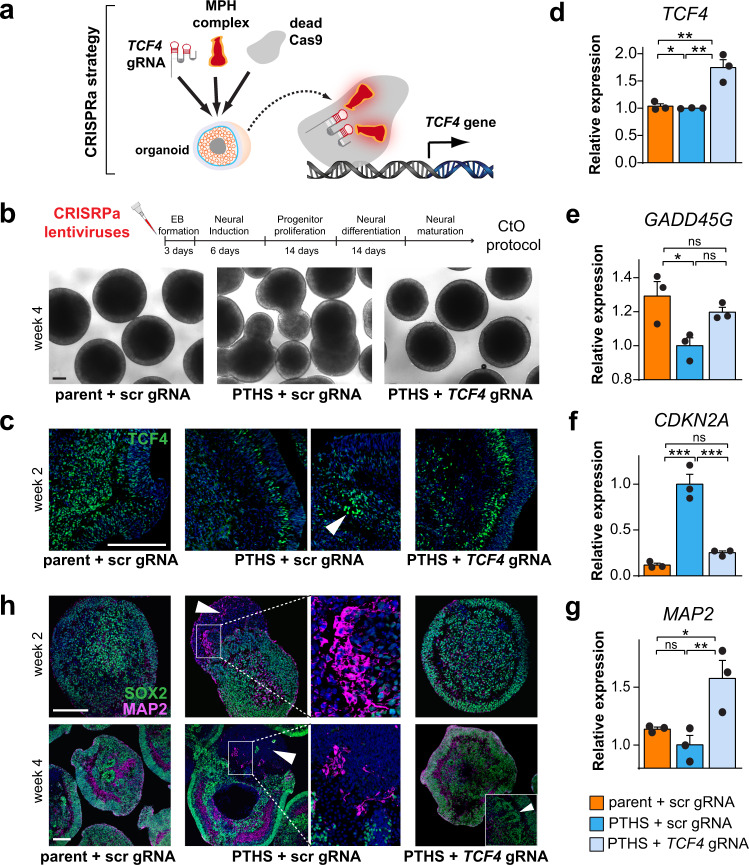
Reversal of abnormal phenotypes in PTHS organoids after trans-epigenetic correction of *TCF4* expression. **a** Schematic representation of CRISPR-based trans-epigenetic correction of *TCF4* expression using constructs for guide RNA (gRNA), transcriptional activation module MPH, and dead Cas9 (see “Methods” for details). **b** Top: Virus application regimen. Bottom: Brightfield images of PTHS brain organoids at 4 weeks in vitro after correction of *TCF4* expression (PTHS + *TCF4* gRNA), compared with controls transduced with scrambled gRNA (scr gRNA). **c** Fluorescence microscopy images of transduced organoids at 2 weeks in vitro after immunostaining for TCF4 (green). Clustered TCF4+ cells (arrowhead) can be seen in aberrant outgrowth in image on the right in PTHS + scr gRNA condition. See Supplementary Fig. 12d, e for quantification of number of TCF4+ cells and mean TCF4 staining pixel intensity in transduced organoids. **d** Increase in *TCF4* expression levels after CRISPR-mediated trans-epigenetic *TCF4* correction in organoids at 2 weeks in vitro. *N* = 3 replicates per group (circles). **e**–**g** Expression levels of *GADD45G* (**e**), *CDKN2A* (**f**), and *MAP2* (**g**) in organoids at 4 weeks in vitro after trans-epigenetic *TCF4* expression correction. *N* = 3 replicates per group (circles). Organoids are from parent–patient pair #4. **h** Transduced organoids stained for MAP2 (magenta) and SOX2 (green), at two developmental time points. Arrowheads in middle panels: polarized PTHS organoids. High mag insets: clustered abnormally shaped MAP2+ cells in polarized organoid outgrowth. Arrowhead in right panel: neural rosettes. Experiments were conducted with organoids from parent-patient pair #4 (circle symbols in bar graphs). Colors in bar graphs represent parents (orange), PTHS (blue), or genetically manipulated PTHS (light blue) groups. Error bars represent SEM. n.s., not significant; *p < 0.05; **p < 0.01; ***p < 0.001; one-way ANOVA followed by Tukey’s HSD *post-hoc* test in bar plots. Scale bars are 100 μm. DAPI nuclear staining in blue. See Supplementary Data 1 for sample and effect sizes and exact *p*-values. Attribution of DNA image in **a**: Ioana Davies/Shutterstock.com.

It is unknown if the PTHS pathophysiology can be corrected in human tissues. Our Wnt manipulation experiments indicate that some cellular phenotypes are amenable to correction, but the Wnt pathway acts downstream of TCF4 and may not correct all aberrant molecular and cellular characteristics of PTHS. Therefore, we decided to perform genetic manipulation of *TCF4* itself. First, we corrected *TCF4* expression in PTHS organoids using a recently described CRISPR-based trans-epigenetic strategy^51^. In this method, two viral vectors are used to deliver three expression cassettes to target cells: one encodes a short guide RNA (gRNA) coupled with an engineered RNA hairpin aptamer; the second encodes a transcriptional activation complex (MPH) that binds the aptamer; and the third encodes dead Cas9 (see “Methods” for details). Expression of these cassettes in target cells epigenetically transactivates the endogenous *TCF4* locus, because Cas9-mediated gRNA binding to the *TCF4* promoter congregates MPH, enhancing transcription from the downstream gene (Fig. 8a).

We created a collection of expression cassettes containing 15 different gRNAs targeting the three most active alternative promoters of the *TCF4* gene (Supplementary Fig. 12a). Some gRNAs were found to efficiently transactivate *TCF4* and its target genes in the neuronal cell line SH-SY5Y, and a few gRNAs ideally provided a 2-fold *TCF4* expression increment (Supplementary Fig. 12b, c). Next, we used lentiviral vectors to transduce the expression cassette containing the most efficient gRNA, together with MPH/Cas9 cassettes, into PTHS organoids derived from patient #4 and its respective parent control (Fig. 8b). We chose this patient line because it shows the largest differences in organoid size and cellular content compared to the respective control (Figs. 1h, 2b), allowing us to identify the benefits of *TCF4* correction more easily. We verified that *TCF4* correction led to an increase in TCF4 immunolabeling intensity (Fig. 8c and Supplementary Fig. 12d, e) and *TCF4* mRNA levels (Fig. 8d). As expected, *TCF4* expression in PTHS line #4 is not substantially reduced because it contains a point mutation (Fig. 8d and Supplementary Fig. 1a), but our CRISPR-mediated correction strategy enhanced both alleles (Supplementary Fig. 12f), and the globally increased TCF4 level (Fig. 8c and Supplementary Fig. 12g) was accompanied by correction of the downstream target *GADD45G* (Fig. 8e), revealing functional correction of the *TCF4* locus.

Organoids transduced with the *TCF4* gRNA vector displayed a decrease in *CDKN2A* expression, a correction of *MAP2* expression (Fig. 8f, g), and a partial correction of *SOX3* expression (Supplementary Fig. 12h). Importantly, the PTHS phenotypic abnormalities were rescued in the genetically corrected organoids (Fig. 8h), re-establishing normal rosette-bearing spheroids devoid of aberrant polarization.

We also investigated the presence of immature neurons in PTHS organoids, using DCX (doublecortin) as a marker, which might indicate alterations in the formation of cortical neurons. Although DCX+ cells were found in both parent and PTHS organoids (Supplementary Fig. 12i, j), these cells formed bundles of aberrantly shaped DCX+ fibers of high caliber in PTHS organoids (Supplementary Fig. 12i), a feature that was fixed upon *TCF4* correction. Interestingly, *DCX* expression is lower in the post-mortem PTHS brain cortex tissue sample (Supplementary Fig. 12k), in PTHS CtOs and GbOs (Supplementary Figs. 5b, 8c, 11g and 12l), and in PTHS neurons in 2D culture (Supplementary Fig. 12m). Notably, *DCX* expression increases in the PTHS organoids transduced with *TCF4* gRNA (Supplementary Fig. 12n), further suggesting that the number of immature neurons returns to normal after correction.

**Fig. 9:**
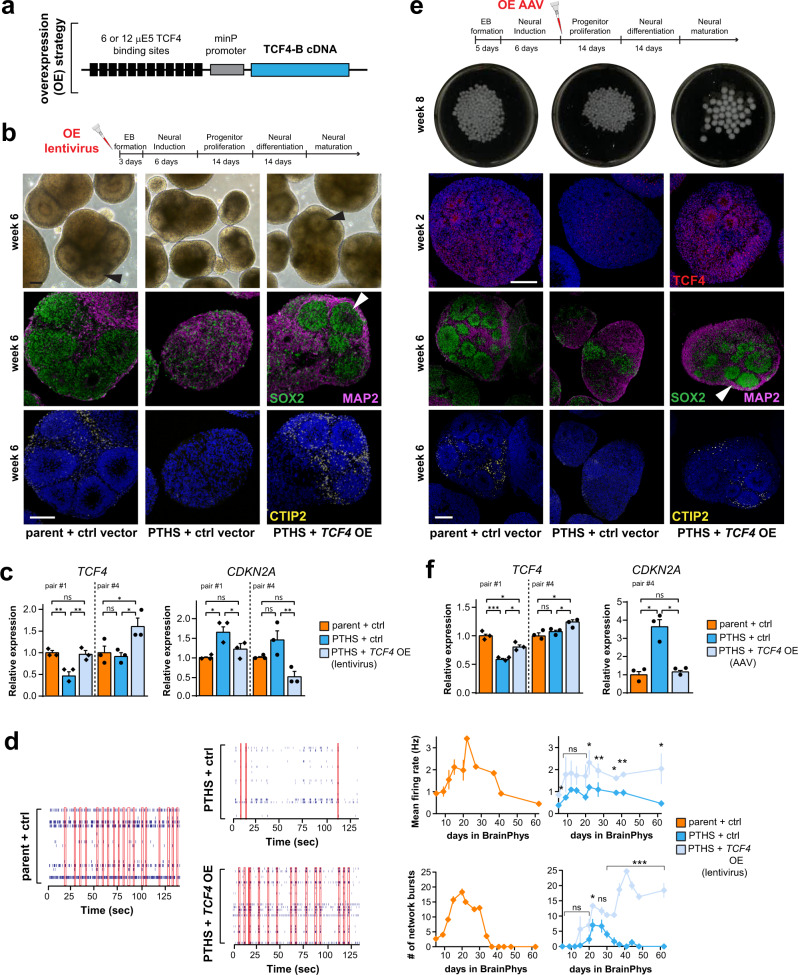
Reversal of PTHS abnormal phenotypes after *TCF4* over-expression in organoids. **a** Over-expression construct, with TCF4-B under the control of varying numbers of TCF4 binding sites (μE5 boxes). **b** Top: Virus application regimen. Bottom: Microscopy images showing general morphology of CtOs at 6 weeks in vitro after transduction with *TCF4* OE vector or a control vector (ctrl; see “Methods” for details) (first line), immunostaining for SOX2 and MAP2 (second line), and immunostaining for CTIP2 (third line). Arrowhead: neural rosette. **c** Relative expression (RT-qPCR) of *TCF4* and *CDKN2A* in CtOs subjected to lentivirus-mediated *TCF4* OE at the beginning of the derivation protocol and evaluated at 2 weeks in vitro. *N* = 3 biological replicates per subject, with organoids from parent-patient pairs #1 and #4. See Supplementary Fig. 13c for densities of SOX2+ and CTIP2+ cells. **d** Left: Raster plots showing electrical activity of transduced CtOs (parent-patient pair #1) at 2 to 3 months in vitro subjected to multi-electrode array (MEA) analysis. Each row of spikes represents an electrode. Vertical red rectangles represent events of network bursts of electrical activity. Right: Quantification of mean firing rate (top) in transduced organoids (see Supplementary Fig. 13d for raster plots), and number of network bursts (bottom). *N* = 3 independent replicates per group, 10 seeded organoids per replicate/group. **e** Top: Virus application regimen. Bottom: Fluorescence microscopy images of transduced CtOs after immunostaining for TCF4 (first line; 2 weeks in vitro), SOX2 and MAP2 (second line; 6 weeks in vitro), or CTIP2 (third line; 6 weeks in vitro). Arrowhead, neural rosette. **f** Top: Relative expression of *TCF4* and *CDKN2A* in CtOs subjected to AAV-mediated *TCF4* OE after the end of the neural induction phase and evaluated at 2 weeks in vitro. *N* = 3 replicates per subject, for organoids from parent/child pairs #1 or #4. See Supplementary Fig. 13f for densities of SOX2+ and CTIP2+ cells. Symbols in bar graphs indicate parent-patient identities: diamonds, pair #1; circles, pair #4. Colors in bar and line graphs represent parents (orange), PTHS (blue), or genetically manipulated PTHS (light blue) groups. Error bars represent SEM. ns, non-significant; *p < 0.05; **p < 0.01; ***p < 0.001; one-way ANOVA followed by Tukey’s HSD *post-hoc* test. Scale bars are 100 μm. DAPI nuclear staining in blue. See Supplementary Data 1 for sample and effect sizes and exact *p*-values.

The CRISPR strategy described above has the disadvantages of requiring two viral vectors, which slightly reduced cellular aggregation on the first days of the organoid derivation protocol (see “Methods” for details). Therefore, we tested a second procedure for correcting *TCF4* levels, in which cells and organoids were subjected to virus-mediated over-expression (OE) of an extra-copy of the *TCF4* gene under the control of TCF4 binding motifs (μE5 boxes) (Fig. 9a, b), an approach expected to prevent ectopic *TCF4* expression. First, we confirmed that *TCF4* and *GADD45G* levels were corrected in transduced PTHS NPCs (Supplementary Fig. 13a). We observed that transduction of organoids with lentiviral *TCF4* OE constructs at the beginning of the derivation protocol led to increased intensity of TCF4 labeling (Supplementary Fig. 13b), corrected *TCF4* and *CDKN2A* levels (Fig. 9c), presence of abundant neural rosettes (Fig. 9b), normal morphology, and rescued numbers of SOX2+ progenitor and CTIP2+ cortical neurons (Fig. 9b and Supplementary Fig. 13c). Importantly, PTHS organoids subjected to *TCF4* OE displayed a significant improvement in two key electrophysiological parameters indicative of functional rescue—mean firing rate and number of network electrical bursts (Fig. 9d and Supplementary Fig. 13d).

When *TCF4* OE was performed after the neural induction phase using AAV vectors (Fig. 9e), we also achieved an increase in TCF4 labeling (Fig. 9e and Supplementary Fig. 13e), corrected *TCF4* and *CDKN2A* expression (Fig. 9f), increased numbers of SOX2+ and CTIP2+ cells (Supplementary Fig. 13f), and reappearance of abundant rosettes (Fig. 9e). This experiment proves that *TCF4* loss-of-function does not result in impaired neural induction, because the PTHS phenotypes were corrected when *TCF4* OE was applied after the neural induction phase.

In combination, these manipulative experiments confirm that the PTHS alterations are due to diminished *TCF4* expression. Importantly, they prove that the cellular pathology associated with *TCF4* loss-of-function—including impaired progenitor proliferation, abnormal neuronal differentiation, and dysregulated cellular senescence and expression of *SOX* genes—can be genetically corrected during neurodevelopment.

## Discussion

**Fig. 10:**
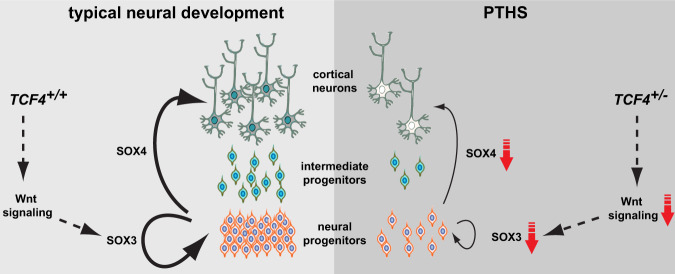
Model of dysregulated pathways underlying PTHS pathophysiology. Mechanistic model to explain aberrant cellular phenotypes in PTHS neural structures. Due to *TCF4* loss-of-function in PTHS, Wnt signaling activity diminishes, in turn leading to decreased *SOX3* expression in NPCs, impairing proliferation. Moreover, we observed that *SOX4* is also downregulated in PTHS cells, which we suggest impairs neuronal differentiation and content in the PTHS neural tissue.

Our data support a model according to which *TCF4* loss-of-function leads to impaired Wnt signaling activity in NPCs, causing lower proliferation (Fig. 10). Importantly, we highlight a possible route for Wnt pharmacological intervention to correct this aberrant phenotype. We also show a novel mechanistic link between transcription factor *SOX3* and PTHS NPC pathophysiology (Fig. 10). Interestingly, mutations in *SOX3* have been associated with another neurodevelopmental disorder, X-linked mental retardation^52^, suggesting the existence of an overlapping molecular mechanism between such a condition and PTHS.

We also hypothesize that *TCF4* loss-of-function mechanistically leads to *SOX4* downregulation, resulting in decreased neuronal differentiation (Fig. 10), a possibility that could be tested via manipulative experiments to overexpress *SOX4* in a sustained and conditional fashion. Our observation that PTHS NPCs exhibit impaired differentiation into neurons is in keeping with the pro-neural roles of transcription factors NEUROG1, NEUROG2, and ASCL1^53^, with which TCF4 is known to biochemically interact^22,54^.

Interestingly, PTHS NPCs and organoids have abnormal expression of *HOPX* (Supplementary Fig. 7). Further morphological and fate mapping experiments should be conducted to determine if *HOPX*+ cells in organoids are oRGs or astrocytes. It will also be interesting to verify if *HOPX* is differently regulated by TCF4 in distinct brain areas, which would justify why *HOPX* expression is downregulated in PTHS CtOs but up-regulated in GbOs. Finally, further studies should probe the interesting hypothesis that altered oRG content and/or function contribute to the aberrant proliferation of PTHS NPCs or differentiation into cortical neurons.

Importantly, we provide a direct report of the histological characteristics in the human PTHS brain (Figs. 1 and 3 and Supplementary Fig. 2), confirming that the aberrant features discovered in vitro exist in a post-mortem PTHS cortex sample. The lower neuron content in the post-mortem tissue is consistent with abnormalities observed in some children with PTHS via MRI, including small or absent *corpus callosum* (Supplementary Table 1)^23^. Strikingly, the significant reduction in CTIP2+ cells in the post-mortem cortex from a child patient clearly indicates that this state is not incompatible with life and does not seem to be associated with more severe symptoms, as this individual (PTHS #6) presented with mild symptomology (Supplementary Table 1). As more samples are analyzed in vitro, statistical power will allow the identification of correlations between levels of neuronal loss and severity of clinical symptoms across patients. Another interesting phenomenon to be further explored is the presence of aberrant cellular migration in the PTHS neural tissue, as suggested by the polarized structure of some PTHS organoids, particularly the causal link with Wnt dysregulation and/or aberrant β-catenin expression.

*Tcf4*^*−/−*^ mice carrying homozygous loss-of-function mutations exhibit altered populations of cortical neurons. However, the phenotypes of *Tcf4*^*+/−*^ heterozygous mice are significantly milder^26,27^ and less prominent than what we observed in the post-mortem PTHS cortical sample and in organoids. This suggests that mouse models are insufficient for studying the consequences of clinically relevant *TCF4* mutations, which happen in heterozygosity. Human brain organoid models provide thus a window of opportunity to observe neural abnormalities relevant to PTHS, although it should be emphasized that they are good models for neurodevelopment, and therefore other systems are necessary to illuminate PTHS pathophysiology in the fully formed neural tissue.

Our manipulative experiments to genetically correct *TCF4* expression in the PTHS neural tissue provide a gateway for the development of targeted therapeutics against PTHS, as well as clinically similar diseases caused by mutations in downstream TCF4-target genes^38^ or even schizophrenia, which may have *TCF4* as a genetic component^11,13^. Interestingly, because the CRISPR-mediated correction of *TCF4* expression simultaneously rescued phenotypes and enhanced transcription from both the mutated and normal endogenous alleles, this experiment also unanticipatedly suggests that PTHS is caused by *TCF4* haploinsufficiency and not by a dominant negative effect^16–18,22^, an important point that should be explored in future studies.

## Methods

### Human subjects

Subjects are members of volunteering families recruited through the Pitt Hopkins Research Foundation or the University of Campinas. Patients with PTHS (Supplementary Table 1) were selected based on availability of detailed clinical and molecular diagnostics information, including the types of *TCF4* mutation they carry. For patients harboring a point mutation, small indel, or translocation, we confirmed the details of each *TCF4* mutation via directing resequencing of the *TCF4* locus. Additionally, we used RNA-Seq data from NPCs and neurons to validate the identities of the cell lines employed in the study (see below). A detailed and personalized questionnaire to gather information related to the patients’ PTHS clinical symptoms was answered by all participating families, encompassing questions about neurological findings, cognitive, behavioral and gastroenterological manifestations, age at diagnosis, general quality of life, temporal evolution of motor milestones, communication level, dysmorphic facial features, urological symptoms, vision problems, sensory responsivity, sleep disturbances, respiratory anomalies such as apnea and hyperventilation, feeding habits and bowel symptoms, history of seizures, as well as MRI findings. These data are reported in Supplementary Table 1. To maximize comparability, we selected only male patients with PTHS for the histological and manipulative experiments in this study, and they are 4 to 14 years old (patients #1 to #5 in Supplementary Table 1). Control subjects were the patients’ corresponding fathers (30 to 50 years old), who had no history of psychiatric or genetic disorders. The post-mortem PTHS brain cortex sample is from a female individual who died during a surgical procedure to correct scoliosis, due to complications unrelated to the PTHS neurological symptoms (patient #6; Supplementary Table 1). The participation of all subjects was approved by the Human Subjects Ethics Committees of the institutes in which the study was conducted (University of California San Diego IRB/ESCRO Committee and University of Campinas Ethics on Human Subjects Committee). Written informed consent was obtained from all participating families after receiving a thorough description of the study and no compensation was provided to participants.

### Note on *TCF4* gene nomenclature

It is important to note that *TCF4* (Transcription Factor 4) should not be confused with *TCF-4* (T-Cell factor 4), an old and outdated name for *TCFL7*, the gene coding for one of the TCF-LEF proteins, the endpoints of the Wnt signaling pathway, which are totally unrelated to the *TCF4* mutated in PTHS.

### Reprogramming of skin fibroblasts into iPSCs

Skin fibroblasts were obtained from biopsies taken from PTHS and control subjects, followed by culturing in DMEM/F12 medium containing 10% fetal bovine serum and penicillin/streptomycin. iPSCs were derived from fibroblasts via cellular reprogramming, as described in Marchetto et al.^41^. Briefly, fibroblast cultures were transduced with Sendai viruses containing over-expression cassettes for *OCT4*, *SOX2*, *KLF4*, and *MYC* (Cytotune iPS 2.0 Sendai reprogramming kit; Thermo Fisher Scientific). Seven days after transduction, cells were re-plated onto a feeding layer composed of murine embryonic fibroblasts (mEFs) in DMEM/F12 containing 20% Knockout Serum Replacement (Thermo Fisher Scientific), 1% non-essential amino acids (NEAA), and 100 μM β-mercaptoethanol. iPSC colonies were identified after 2 weeks and transferred to 6 cm plates coated with Matrigel (BD Biosciences), after which time they were maintained in mTeSR1 Plus medium (Stem Cell Technologies) and passaged by manual picking with the aid of a pipette tip. A total of 20 iPSC clonal lines were produced for each subject in the study, all of which were analyzed through a combination of immunostaining and SNP mapping to rule out the presence of unwanted chromosomal abnormalities and mutations (example in Supplementary Fig. 1c). All iPSC clones were then passaged until P10 and 2 clones were chosen for further NPC and organoid derivation after this passage. Most results reported in this paper are from experiments conducted with one or two P15 iPSC clones per subject, and confirmation of consistency in the observed phenotypes was obtained from 2 independent iPSC clones per subject (Supplementary Fig. 2a). Cultures were tested every two weeks for mycoplasma, and contamination was never identified at any stage.

Validation of iPSC clones was performed via immunostaining for SOX2, OCT4, NANOG, and LIN28. Briefly, a total of 20 colonies were grown inside wells of LabTek II 8-well chambered slides (Thermo Fisher Scientific) until they reached a diameter of 2 mm. Colonies were then fixed with 4% paraformaldehyde solution for 10 min, washed once with 1× Phosphate Buffered Saline (PBS), permeabilized with 1% Triton X-100 for 5 min, washed again in 1× PBS, and blocked with 10% Bovine Serum Albumin (BSA)/1% Triton X-100/1× PBS. Incubation with primary antibodies was performed in the same blocking solution for 16 h at 4  °C. Primary antibodies used were rabbit anti-SOX2 (Abcam; ab97959; 1:1000), rabbit anti-OCT4 (Abcam; ab19857; 1:100), rabbit anti-NANOG (GeneTex; GTX100863; 1:100), and rabbit anti-LIN28 (Cell Signaling; 3978; 1:500). After 3 washes in 1× PBS, colonies were incubated with fluorescently labeled secondary antibodies for 3 h, and nuclei were counterstained with 1 μg/mL DAPI (Thermo Fisher Scientific) for 30 min. Slides were mounted with ProLong Gold anti-fading solution (Thermo Fisher Scientific).

For identifying unwanted chromosomal structural alterations, genome-wide profiling for amplifications, deletions, copy number variation, and rearrangements was performed via SNP mapping-based karyotypic analysis on genomic DNA extracted from the iPSC lines, using the iScan system (Illumina) and the Infinium HumanCytoSNP-12 BeadChip (Illumina; 299,140 genetic markers). Clones containing visibly large deletions and duplications were not found. An example of karyotyping conducted using this technique is presented in Supplementary Fig. 1c for patient #2, showing the expected deletion in the long arm of chromosome 18 for this patient line.

### Derivation of pallial and GABAergic-enriched organoids

For the generation of pallial (cortical) brain organoids (CtOs), we used our previously published protocol^35^. Briefly, iPSC colonies were dissociated using Accutase (Thermo Fisher Scientific; diluted with an equal volume of 1× PBS) for 12 min at 37 °C. After centrifugation for 3 min at 150 × *g*, the individualized cells were resuspended in mTeSR1 Plus medium (Stem Cell Technologies) supplemented with 10 mM SB431542 (Stemgent) and 1 mM dorsomorphin (R&D Systems). Approximately 3–4 million cells were seeded onto each well of a low-binding 6-well plate and placed on a shaker inside a CO_2_ incubator at 95 rpm. During the first 24 h, medium was supplemented with 5 mM Rho kinase inhibitor (Y-27632; Calbiochem, Sigma-Aldrich). Over three days, cells clustered to form spherical embryoid bodies, after which time mTeSR1 Plus medium was replaced with neural induction medium consisting of Neurobasal medium (Thermo Fisher Scientific) containing GlutaMAX, 1% Gem21 NeuroPlex supplement (Gemini Bio-Products), 1% N2 NeuroPlex supplement (Gemini Bio-Products), 1% NEAA (Thermo Fisher Scientific), 1% penicillin/streptomycin (Thermo Fisher Scientific), 10 mM SB431542, and 1 mM dorsomorphin, for 7 days. Next, such medium was replaced with NPC proliferation medium, consisting of Neurobasal medium containing GlutaMAX, 1% Gem21, 1% NEAA, 20 ng/mL FGF-2 (Thermo Fisher Scientific) for 7 days, followed by 7 additional days in the same medium further supplemented with 20 ng/mL EGF (PeproTech). Neuronal differentiation and organoid maturation were achieved by switching to Neurobasal medium containing 1% GlutaMAX, 1% Gem21, 1% NEAA, 10 ng/mL of BDNF, 10 ng/mL of GDNF, 10 ng/mL of NT-3 (all from PeproTech), 200 mM L-ascorbic acid, and 1 mM dibutyryl-cAMP (Sigma-Aldrich), for 7 days. After this period, CtOs were maintained in Neurobasal medium containing GlutaMAX, 1% Gem21, 1% NEAA for as long as needed, with media changes every 3-4 days. For every subject, most experiments were conducted with at least 3 independent batches, which were considered independent biological replicates in experiments throughout the study and in Supplementary Data 1, with at least 3 technical replicates (wells of organoids) per batch. For phenotypic evaluations conducted on 4 or more separate batches, we used two or more independent clones of iPSCs to produce the organoids (and NPCs) and to confirm the effect of genotype, as depicted in Supplementary Fig. 2a.

For the generation of GABAergic-enriched organoids (GbOs), we used the SHH pathway agonist SAG to divert neural specification to the inhibitory interneuron lineage, an approach adapted from a strategy used to generate organoids containing high content of GABAergic neurons^33^. After culturing embryoid bodies in mTeSR1 Plus for three days, they were transferred to neural induction medium (Neurobasal medium supplemented with 1% GlutaMAX, 1% Gem21, 1% N2, 1% NEAA, 1% penicillin/streptomycin, 10 mM SB431542, and 1 mM dorsomorphin) containing 5 μM IWP-2 (SelleckChem), from day 4 until day 10. Next, the medium was replaced with NPC proliferation medium, consisting of Neurobasal medium containing 1% GlutaMAX, 1% Gem21, 1% NEAA, 20 ng/mL FGF-2, and 100 nM SAG (SelleckChem) for 7 days, followed by 2 additional days in the same medium supplemented with 20 ng/mL EGF (PeproTech). Culturing for an additional 5 days in the same medium, but without SAG, completed the NPC proliferation phase. This was followed by neuronal differentiation and organoid maturation phases, which were conducted using the same types of medium and durations used in the CtO derivation protocol.

### Immunofluorescence staining

After being cultured in vitro for the required amount of time, CtOs and GbOs were fixed with 4% paraformaldehyde for 4–8 h at 4 °C and cryoprotected in 30% sucrose for 12 h. Organoids were then embedded in TissueTek (Leica Microsystems) and sectioned on a Leica CM1850 cryostat to produce 20 μm sections. For staining, slides were air-dried for 10 min, permeabilized in 1% Triton X-100/1× PBS for 2 min, and blocked with 0.1% Triton X-100/3% BSA/1× PBS for 1 h at 25 °C, followed by incubation with primary antibodies in the same solutions, for 16 h at 4 °C. Primary antibodies used were: rat anti-CTIP2 (Abcam; ab18465; 1:500); rabbit anti-SATB2 (Abcam; ab34735; 1:200); chicken anti-MAP2 (Abcam; ab5392; 1:1000); rabbit anti-SOX2 (Cell Signaling Technology; 2748; 1:500); rabbit anti-GAD65/67 (Abcam; ab11070; 1:200); rabbit anti-CUX1 (CUTL1 or CASP) (Abcam; ab54583; 1:200); rabbit anti-TCF4 (Abcam; ab217668; 1:1000); rabbit anti-vGLUT1 (Synaptic Systems; 135311; 1:500); rabbit anti-CC3 (Cleaved Caspase 3) (Cell Signaling; 9664 S; 1:500); rabbit anti-doublecortin (DCX) (Abcam; ab18723; 1:200); mouse anti-Cas9 (Abcam; ab210571; clone [8C1-F10]; 1:200); mouse anti-p16^INK4a^ (CDKN2A) (Abcam; ab54210; clone [2D9A12]; 1:1000); rabbit anti-SOX3 (Abcam; ab183606; 1:200); mouse anti-Nestin (Abcam; ab22035; clone [10C2]; 1:1000); goat anti-SOX17 (R&D Systems; AF1924; 1:200); rabbit anti-Brachyury (Sigma; B8436; 1:200); rabbit anti-β-catenin (Cell Signaling Technology; 9582 S; 1:100); rabbit anti-FOXG1 (Abcam; ab196868; 1:500); mouse anti-AP2 (TFAP2A) (Thermo Fisher; MA1-872; clone [3B5]; 1:50); rabbit anti-HOPX (Abcam; ab230544; 1:100); or rabbit anti-c-Fos (EMD Millipore; PC38, 1:1000). After incubation in a solution containing primary antibodies, slides were washed three times in 1× PBS, for 5 min each, and incubated with fluorescently labeled secondary antibodies (Alexa Fluor 488- or 555-conjugated antibodies; 1:500 dilution; Thermo Fisher Scientific) in the same type of solution as primary antibodies, for 3 h at 25 °C. After further washes in 1× PBS, slides were counterstained with DAPI solution (1 μg/mL) for 45 min and mounted with ProLong Gold. All images were taken using a Zeiss fluorescence microscope equipped with Apotome (Axio Observer Apotome, Zeiss).

For projections of z-series stacked images of DCX-stained organoids (Supplementary Fig. 12i), we used the maximum intensity feature of ZEN Lite software (Zeiss; version 3.1) after collecting 10 optical slices per section. For p16^INK4a^ staining, we applied antigen retrieval by incubating the slides at 60 °C for 10 min in 1× Universal HIER antigen retrieval reagent (Abcam; ab208572), followed by regular immunostaining. For counting SOX2+ cells after p16^INK4a^ co-staining (Supplementary Fig. 6g, h), we used raw unprocessed images, and defined strongly stained cells as those which had average pixel intensity between the upper third quartile and the maximum pixel intensity in each image. The remaining SOX2+ cells were considered weakly stained.

For the quantification of cell types in organoid sections, 3 or 4 random 100 ×100 μm regions of interest (ROI) were sampled across each imaged section. The mean number of labeled cells per sample was calculated by first averaging the number of labeled cells in each ROI to produce a mean value of labeled cells per section, and then averaging these mean values across all sections for each subject. The number of subjects and sections quantified are indicated in the figure legends and in Supplementary Data 1. Because vGLUT1 is mostly not found in the cell bodies, we chose to quantify vGLUT1 and GAD65/67 (Supplementary Fig. 2g) by counting pixels in raw unprocessed fluorescence microscopy images using the Color Pixel Counter plugin on the ImageJ software (version 1.53o), counting particles of size 1 pixel and color intensity above 50 (in a range from 0 to 255). The average percentage of pixels according to these rules was computed over four 100 × 100 μm ROIs per section and 6 sections per subject. Similarly, we used the Color Pixel Counter plugin on ImageJ to measure pixel intensity of TCF4 immunostaining in organoids subjected to transduction to correct *TCF4* expression (Supplementary Fig. 12e), setting color intensity in a range from 0 to 255 for each particle of size 1 pixel. The average pixel intensity was computed over 4 100 × 100 μm ROIs per section, with 3 or 4 sections per group. For the quantification of the numbers of c-Fos+ cells per unit of area in CtOs at 6–8 weeks in vitro (Supplementary Fig. 5e), we counted the numbers of cells with unequivocal c-Fos nuclear staining in an area of 300,000 μm^2^ at the organoid’s periphery, choosing an outer margin of length 300 μm along the surface of the organoid. We chose to analyze this ROI because c-Fos+ cells in unstimulated organoids tend to be localized close to the organoid surface, as previously reported^55^, where neurons mature in large numbers outside the underlying progenitor-rich neural rosettes and are exposed to high concentrations of neuronal maturation factors in the surrounding medium. For the quantification of the percentages of c-Fos+ cells in the organoids, we computed the number of immunostained cells over the number of DAPI-stained nuclei in the organoids. These analyses of c-Fos protein expression were performed as an additional line of investigation to support the data showing that PTHS organoids have decreased activity and to rule out the possibility that the expression of *FOS* gene in organoids is a consequence of the cellular dissociation applied prior to the generation of scRNA-Seq libraries. Because the organoids were immediately fixed and processed for immunostaining and because the transiently expressed c-Fos protein informs of activity that occurred 45 to 90 min prior to fixation (long before the harvesting procedure), this approach allowed us to confidently capture activity levels in parent and PTHS organoids without possible confounding factors. It should be noted that most c-Fos+ neurons in the organoids exhibit staining at the periphery of the nucleus. We hypothesize that this peripheral pattern of staining is a combination of the use of short exposure times and apotome-mediated imaging, which produces pixel-normalized images that capture the staining in a very thin optical section, detecting the highest concentrations of c-Fos protein at the nucleus periphery.

For immunofluorescence labeling of NPCs, these cells were seeded at a density of 50,000 cells per well of a LabTek II 8-well chambered slide. When cells reached 50% confluency, they were fixed and processed for immunostaining in the same manner as described for iPSC colonies, with the following primary antibodies: rabbit anti-TCF4 (Abcam; ab217668; 1:1000); and chicken anti-vimentin (VIM) (Abcam; ab22651; 1:2000). NPCs were also stained to detect senescence-associated β-galactosidase (SA-β-gal), using the CellEvent Senescence Green Detection Kit (Thermo Fisher Scientific; C10850) after antigen retrieval as described above. The same method described above for counting weakly and strongly stained SOX2+ cells after p16^INK4a^ co-staining was applied to NPCs in Supplementary Fig. 6g.

### Post-mortem brain sample collection and analysis

Hospital pathologists dissected the brain from patient #6 immediately after death and harvested cortical tissue encompassing the entire width of the cortex at the boundary between the pre-motor and prefrontal areas. Hippocampus tissue was also harvested but is not described in this study. Brain tissue was fixed for 24 h under formalin, then fixed in 4% paraformaldehyde for 6 h, prior to being cryoprotected in 20% sucrose and sectioned under a vibratome followed by immunostaining, as described above. PTHS images were compared with those obtained from control sections stained in parallel (Figs. 2f, 7e) using normal brain tissue from a commercial source (NOVUS; NBP2-77523), harvested from a 12 years old male without signs of disease or neuropathology. Comparisons were performed with matching images collected from ROIs at equivalent depths (measured in millimeters from the cortex surface in Supplementary Fig. 2i), because the use of cortical layering structure as a proxy was not possible for PTHS brain tissue, due to its characteristic disorganized histology. No significant difference was observed by the pathologists in terms of general appearance of the brain gyri and width of the cortex tissue prior to dissection.

### Organoid single cell RNA sequencing analysis

Single cell RNA-Seq was performed on dissociated CtOs and GbOs at 8 weeks in vitro, totaling 8 libraries, consisting of 3 replicate libraries of independently produced CtOs from parent #4 and one library each of PTHS #4 CtOs, parent #4 GbOs, PTHS #4 GbOs, CHIR99021-treated PTHS #4 CtOs, and CHIR99021-treated PTHS #4 GbOs. For each library, a total of 15 organoids (5 organoids from each of three wells from independent experiments) were dissociated to produce a single cell suspension via a combination of mechanical dissociation with forceps and enzymatic digestion with Accutase for 10 min, and the resulting cells were pooled and subsequently filtered to isolate single cells for RNA sequencing analysis on the same day. Dissociated cells were pelleted (3 min, 100 × *g*, 4 **°C**) and resuspended in 10 mL of Neurobasal medium. We determined the concentration of single cells in each library using the Chemometec automatic cell counter, and the population viability across all libraries was found to be within the range 85–92%. Single cell RNA-seq libraries were prepared using the Chromium Single Cell 3’ v3 Library kit (10× Genomics) according to the manufacturer’s protocol. We loaded approximately 20,000 cells per sample on the Chromium chip. All steps, including GEM (Gel beads in emulsion) preparation, reverse transcription, PCR amplification, and Illumina library construction were carried out on a T100 thermal cycler (Bio-Rad). cDNA extracted from GEMs was cleaned up using MyOne Silane Beads (Thermo Fisher Scientific), PCR-amplified for a total of 10 cycles, and then purified using SPRIselect Reagent Kit (B23317, Beckman Coulter). Next, we enzymatically fragmented the cDNA pool for each library and performed a double size selection using the SPRIselect Reagent Kit. Finally, we ligated Illumina adapters to prepare the libraries for sequencing, followed by another round of double size selection with the SPRIselect Reagent Kit. Final library sizes ranged from 300 to 700 bp, with an average size around 450 bp. We quantified the Illumina libraries using Qubit dsDNA HS Assay Kit (Thermo Fisher Scientific) and performed size quality control on a High Sensitivity D1000 tapestation (Agilent). Libraries were sequenced on a NovaSeq 6000 S4 sequencer (Illumina) to produce 20,000 reads per single cell, or 400 million reads per library, with 26 cycles for read_1, 8 cycles for the index, and 98 cycles for read_2, which contains the gene sequence.

Feature count matrices for each single-cell RNA-Seq library were generated separately using the ‘cellranger count’ command in the Cell Ranger software (version 4.0.0) and the GRCh38 2020-A reference dataset of human transcripts. The independent libraries were then normalized to the same sequencing depth and aggregated into a single feature-barcode matrix using the ‘cellranger aggr’ command. The estimated number of cells across all libraries was determined to be within the range 2,944 to 6,466 per library, with a mean number of reads per cell ranging from 58,501 to 169,931.

Cell type subpopulations were delineated via a combination of automated annotation and refinement after manual inspection. First, processed data were transferred to Cell Loupe software (10× Genomics; version 4.1.0) and analyzed to partition groups of single cells using *k*-means clustering (*k* = 8). Next, we visually inspected the expression of each marker gene in Supplementary Fig. 3b in each *k*-mean-assigned subpopulation and manually adjusted the subpopulations based on the expression pattern of these genes. The combined approach of first performing unbiased determination of subpopulations followed by manual refinement maximizes the identification of biologically relevant groups of cells. It is evident that these subpopulations could be further subdivided into other groups of cells, but we decided to focus on groups containing progenitors, intermediate progenitors, and neurons in the excitatory and inhibitory lineages shown by the single-cell data (Fig. 3). Other minor subpopulations (collectively named ‘Others’ in Supplementary Fig. 3a, b) exist but were not displayed in most panels because the focus of our paper was on the 6 subpopulations mentioned above and because the ‘Others’ cells could not be unequivocally assigned to the populations chosen for analysis based on the expression of marker genes. Further analysis led to the conclusion that the numbers of cells in the ‘Others’ category do not differ between parent and PTHS organoids, and they are not cells of non-neural origin, which are not present in conspicuous amounts in CtOs or GbOs (Supplementary Fig. 3g). We employed mitochondrial genes as a proxy for identifying apoptotic cells, which were generally infrequent in all libraries (usually less than 5%) and were excluded from the analyses. Supplementary Data 4 contains associations between cell barcodes and assigned subpopulations, to ensure reproducibility of our results.

Next, we used the Seurat library (version 3.2.2)^56^ for downstream processing and analysis of the feature-barcode matrix. First, the aggregated matrix generated by Cell Ranger was imported into Seurat and normalized by dividing the feature counts of each cell by the total counts for that cell, followed by scaling the data to 10,000 counts per cell prior to performing a ln(count + 1) log transformation (‘NormalizeData’ function). Next, variable features were identified with the ‘FindVariableFunctions’ function, which fits a polynomial curve to the mean-variance relationship, standardizes feature counts based on their expected variance given their expression, and selects the 3000 features with the highest variance. The highly variable features were then scaled to a distribution (‘ScaleData’ function) with mean expression 0 and variance 1 across cells, which was subsequently used to perform linear dimensional reduction (PCA, ‘RunPCA’ function). The first 15 PCA dimensions were used to embed the cells into a non-linear reduced dimension space using the UMAP algorithm (‘RunUMAP’ function; version 0.1.10). The UMAP plot presented in Fig. 3a aggregates data from cells across all 8 libraries sequenced.

Unsupervised trajectory (pseudotime) inference (Fig. 3b) was performed independently for the excitatory and inhibitory lineages using Monocle 3 (version 0.2.2)^57^. Specifically, the Leiden method was used to cluster cells within the UMAP embedding (‘cluster_cells’ function), and unpartitioned principal graphs representing the differentiation trajectories were then fit to the data (‘learn_graph’ function). Finally, cells were ordered by rooting the trajectories at the manually annotated progenitor subpopulations (‘order_cells’ function). Pseudotime is the transcriptional distance (abstract units) between a cell and the start of the trajectory, measured along the shortest path. It should be noted that this pseudotime analysis recognizes patterns of trajectory across the entire groups of cells being analyzed, and therefore the trajectory’s total length is the total amount of transcriptional change that the entire group of cells undergoes from the starting state (root) to the end state. Because each one of our six subpopulations probably contains many types of sub-lineages, each with its own specific temporal differentiation trajectory, it is possible that some cells within the neuronal (N-Glut or N-GABA) subpopulations are assigned to early pseudotime points in the overall analysis. That does not mean that they represent early differentiation stage cells, and analyzing the behavior of the overall group of cells is more important than trying to identify the specific pseudotime position of each cell along the differentiation trajectory.

For DE analysis on the cellular subpopulations of the organoid single-cell transcriptomic data, we created a subset of the main Seurat object to include just the libraries being compared (for example, parent and PTHS CtOs). Next, we used the ‘FindMarkers’ function in Seurat on this subset object, setting the identities (‘ident.1’ and ‘ident.2’ functions) to the subpopulations being compared. The statistical algorithm used was DESeq2 (version 3.14), chosen by setting the ‘test.use’ function to ‘DESeq2’, with a ‘min.pct’ of 0 (minimum percentage of cells expressing the gene) and ‘logfc.threshold’ of 0 (minimum log_2_ of fold change) to produce Supplementary Data 3 with all genes in the dataset. The adjusted *p*-values were calculated by Seurat using Bonferroni correction based on the total number of genes in the dataset. When using the DESeq2 option, the ‘slot’ function is automatically set to ‘counts’, which exponentiates the log-transformed expression data as *e*^log-transformed counts^ - 1 for each cell in the Seurat object prior to calculating the non-log-transformed average expression values and fold-changes (FC). FCs were then converted to log_2_FC values, as presented in Supplementary Data 3.

We used Cell Loupe software to quantify the percentages of cells in each subpopulation and library (Fig. 3d, g and Supplementary Figs. 4a, c, 9a, b, 11c). To calculate the percentage of cells expressing a given gene, we used Seurat and R package to count the number of cells expressing said gene above a threshold level corresponding to 40% of the gene-expression mean in all cells belonging to the groups being compared (Fig. 3e, h–j and Supplementary Figs. 4e, f, h, 5c, 7e, 11d). We decided to use a threshold for identifying and objectively counting cells with detectable expression for each gene because of the existence of cells with very low expression in each group, the inclusion of which in the calculations would not make biological sense. We picked an arbitrary threshold of 40% of the mean gene expression after analyzing the effects of counting the percentages of cells in control versus PTHS organoids with different threshold levels for every gene analyzed in the study, concluding that these percentages do not vary in the interval from 20 to 80% of the mean. The percentages of cells in parent CtOs shown throughout the figures were calculated based on data from the parent replicate #2 library. For statistical comparisons of gene-expression levels between specified subpopulations, we used Mann–Whitney *U* test (for 2 groups) or Kruskal–Wallis test followed by Dunn’s *post-hoc* test (for more than 2 groups and pairwise comparisons).

### NPC derivation and neuronal differentiation

iPSC colonies maintained in mTeSR1 Plus medium were switched to DMEM/F12 medium containing N2 and GEM21 supplements (Stem Cell Technologies). After 2 days, colonies were lifted from the plate with Accutase and cultured in the same medium with the addition of 10 mM SB431542 and 1 mM dorsomorphin, in suspension on a platform shaker, until embryoid bodies formed. After 2 weeks of culturing in this manner, the embryoid bodies were plated directly onto Matrigel-coated dishes and maintained in DMEM/F12 medium containing N2 and SM1 supplements (Stem Cell Technologies), 20 ng/mL FGF-2, and 1% penicillin/streptomycin. After 3 to 5 days, rosettes emerged, and 7 days later the rosettes were manually picked and replated onto Matrigel-coated dishes. NPCs sprouted around the rosettes and were dissociated with Accutase for 5 min prior to being re-seeded onto plates coated with 10 μg/mL poly-ornithine (Sigma-Aldrich) and 5 μg/mL laminin (Thermo Fisher Scientific) to produce passage 1 (P1). NPCs were maintained in DMEM/F12 medium containing N2 and SM1 supplements, 20 ng/mL FGF-2, and 1% penicillin/streptomycin for up to 20 passages. We chose not to derive or culture NPCs in medium containing Wnt or Shh agonists/antagonists, such as cyclopamine, because treatment of progenitors with artificially high concentrations of these substances might affect the cells’ proliferation rate, thereby potentially adding a confounding factor in our evaluations of NPC proliferation.

For neuronal differentiation, NPCs were seeded onto plates coated with poly-ornithine and laminin and cultured in NPC medium until they reached 90% confluency, at which time the medium was changed to DMEM/F12 containing N2 and SM1 supplements and 1% penicillin/streptomycin, with media changes occurring every 3–4 days. When neuronal processes started to grow one week later, the medium was changed to BrainPhys neuronal medium (Stem Cell Technologies) and cells remained under these conditions for up to 4 months, with media changes occurring every 3–4 days.

Electrophysiological measurements in Fig. 4c, d were performed on neuronal cultures after 3 or 4 months in BrainPhys medium, a time point at which the vast majority of cells in the culture are MAP2 + (95.4 ± 2.4% in parent versus 93.2 ± 1.4% in PTHS; *p* = 2.4; unpaired Welch’s *t* test).

Quantification of neuronal differentiation rates (Fig. 7i, m and Supplementary Fig. 11a) was accomplished by counting MAP2+ and SOX2+ cells in differentiating neuronal cultures seeded onto LabTek II chambered slides after 2 months of differentiation in BrainPhys medium, followed by immunofluorescence staining as described above.

### RNA sequencing of NPCs and neuronal cultures

Using the RNeasy Mini Plus kit (Qiagen), RNA was isolated from NPCs of 4 subjects and 4 respective parental controls at passage 15 for most analyses, from NPCs of 2 subjects and 2 respective controls at passage 5 for analysis in Supplementary Fig. 6e, f, 7a and Supplementary Data 2, and from differentiating neuronal cultures of 3 parent-patient pairs and an additional patient line after 2 months in BrainPhys medium followed by FACS sorting to purify the CD184−/CD44−/CD24+ population^58^ (Figs. 1b, 4e and Supplementary Figs. 5j, k, m, 12m). For each subject, RNA was extracted from 3 independently prepared biological replicates. A total of 1 μg of RNA was used from each sample for Illumina library preparation using the stranded TruSeq kit (Illumina). RNAs were sequenced on an Illumina NovaSeq 6000 S4 instrument with 150 bp paired-end reads, generating approximately 40 million sequencing fragments per library.

To estimate transcript-level expression from bulk RNA-Seq data, we used Salmon (version 0.14.1) software^59^, with selective mapping (‘–validateMappings’) and correction for sequence-specific biases (‘–seqBias’), GC-content biases (‘–seqBias’), and fragment position bias (‘–posBias’). Reference transcripts for read mapping were obtained from the GENCODE 32 basic annotation^60^. For every sample, outliers were defined by high between-replicate Euclidean distances (after transformation to achieve homoskedasticity, as described below), which led to the exclusion of just one neuron library replicate from patient #3 from the follow-up expression analysis (see computational codes and quality control results in the repositories described in the Data Availability and Code Availability sections). All remaining 56 libraries passed the quality control phase and were retained.

The transcriptomic data were also used to validate the identities of the cell lines employed in the study. For patients PTHS #1 and #4, the sequences of *TCF4* transcripts encompassing the respective mutated sites were retrieved from GENCODE, followed by the generation of mutated sequences to include the mutations each patient carries (insertion and point mutation, respectively; see Supplementary Table 1). The resulting sequences (Supplementary Data 5) were added to the reference GENCODE transcriptome prior to quantification of transcript expression abundances with Salmon. The quantification results informed that the expression of each mutated *TCF4* transcript is only found in the respective patient and not in the other patients nor in the parent samples (Supplementary Data 5). For patients PTHS #2 and #3, we could not produce the sequences of the mutated transcripts because they carry a whole-gene deletion and a chromosome translocation, respectively (Supplementary Table 1).

Pairwise differential expression (DE) tests between cells derived from each patient with PTHS and his respective parental control were performed with DESeq2 (version 1.22.1)^61^. tximport (version 1.10.1)^62^ was employed to aggregate transcript abundances into gene-level counts. Next, between-sample normalization was performed using the size factors approach^63^ and a local dispersion model was fit to the normalized counts. Lastly, a negative binomial generalized linear model was fit to the data, the effect sizes (log2FoldChange) were shrunken with the apeglm algorithm (version 1.16.0)^64^, and strict statistical testing was accomplished using threshold-based Wald tests (‘lfcThreshold = 0.5’). DE transcripts were determined based on their *s*-values (<0.005). Transformation of count data into an approximately homoskedastic matrix for clustering and visualization purposes (Supplementary Figs. 5j and 7a) was attained with the ‘varianceStabilizingTransformation’ function using the ‘blind’ parameter set to ‘TRUE’.

To obtain lists of DE genes across all subjects, we first derived a list of DE genes between each patient with PTHS and his respective parent (Supplementary Data 2), followed by cross examination of all lists and selection of DE genes common to all child-parent pairs. The final list was used for gene-set enrichment assessment followed by Gene Ontology (GO) and pathway analyses, using the web-based WebGestalt tool (version 2019)^65^, with default parameters. WebGestalt conducts permutations to obtain an over-representation *Z* score and enrichment *p-*value for each GO term. For pathway analysis, we chose the KEGG option, with default parameters. For all analyses, a minimum of 5 genes per category was employed, with BH multiple test correction, and a significance level chosen for a false-discovery rate of 0.05.

For the deconvolution of neuronal RNA sequencing to assess the diversity of neuronal subtypes produced in vitro, we used a matrix containing gene-expression counts (transcripts per million, TPM) for all genes and the ‘Cell Fractions’ module in the CibersortX tool (version 2021)^66^ to enumerate the proportions of distinct cell subpopulations in the bulk tissue expression profiles. First, we used our PTHS and control single cell RNA sequencing data (data aggregated from CtO and GbO libraries) to obtain a custom signature matrix file, which consists of barcode genes that can discriminate each of the six subpopulations analyzed in this study (Pr-Glut, IP-Glut, N-Glut, Pr-GABA, IP-GABA, and N-GABA). The signature matrix was subsequently used to impute cell fractions to each bulk neuronal RNA-Seq library (mixture file), using 1,000 permutations. We employed ‘S-mode’ (single-cell mode) batch correction, which is tailored for single cell-derived signature matrices generated from droplet-based or UMI-based platforms, such as the 10× Genomics Chromium platform used here. The ‘absolute mode’ was used, which scales relative cellular fractions into a score that reflects the absolute proportion of each cell type in a mixture; the final values were converted to relative fractions (which could then be used to compare across samples) by normalizing the absolute scores of the control samples to 100%.

### Real-time quantitative PCR

Total RNA was extracted using the RNeasy Mini Plus kit (Qiagen), followed by DNase I treatment on the column, as per the manufacturer’s recommendations. Two and a half micrograms of total RNA were reverse transcribed into cDNA using the Superscript III First-Strand Reverse Transcription System (Thermo Fisher Scientific). Real-time quantitative PCR (RT-qPCR) was performed using pre-validated FAM-MGB TaqMan probes (Thermo Fisher Scientific) and the TaqMan universal master mix II without UNG (Thermo Fisher Scientific) on a CFX Connect Real Time PCR detection system with a Maestro software (Bio-Rad; version 1.1), with the following cycling parameters: 94 °C for 3 min, followed by 40 cycles of 94 °C for 30 s and 68 °C for 1 min. Amplification and denaturation curves for all probes were analyzed to verify amplification of just one amplicon. All RT-qPCR analyses were conducted using RNA extracted from at least 3 independent biological samples per subject/condition, with 2 or 3 technical replicates for each probe set/sample combination. Normalization was achieved with endogenous control genes *TBP*, *ACTB*, and *GAPDH*, and relative expression was calculated using the traditional ΔΔCt method.

The following TaqMan probes were used: *STMN2* (Hs00199796_m1), *TAC1* (Hs00243225_m1), *INA* (Hs00190771_m1), *SLC17A6* (Hs00220439_m1), *CDKN2A* (Hs00923894_m1), *LMNB1* (Hs01059210_m1), *WNT2B* (Hs00921615_m1), *WNT3* (Hs00902257_m1), *WNT5A* (Hs00180103_m1), *SFRP2* (Hs00293258_m1), *ASCL1* (Hs00269932_m1), *NEUROD1* (Hs00159598_m1), *HES1* (Hs00172878_m1), *SOX2* (Hs04234836_s1), *SOX3* (Hs00271627_s1), *SOX4* (Hs00268388_s1), *TCF4* (Hs00972432_m1), *CNTNAP2* (Hs01034283_m1), *GADD45G* (Hs00198672_m1), *MAP2* (Hs01103234_g1), *VIM* (Hs00185584_m1), *NES* (Hs04187831_g1), *ID3* (Hs00171409_m1), *KCNQ1* (Hs00165003_m1), *BCL11B* (CTIP2) (Hs00256257_m1), *SATB2* (Hs00392652_m1), *CUX1* (Hs00738851_m1), *TBR1* (Hs00232429_m1), *CDH23* (Hs00254446_m1), *PCDH15* (Hs00263709_m1), and *HOPX* (Hs04188695_m1).

### Neuronal morphometric measurements

Neurons were morphologically analyzed (Fig. 4b) using Neurolucida Neuron Tracing Software (MBF Bioscience; version 2017.01.1). Individual MAP2+ neurons were identified from microscopy images that clearly exhibited either the number of processes branching from the cell body, processes of complete root-to-tip length, or complete cell bodies. We only computed neurons whose shortest dendrite was at least 3 times longer than the diameter of the cell soma. Random images from at least 2 clones of each cell line were assessed. The ‘contour’ function was used to trace and sum incremental lengths of each curve along the longest path of a complete process to yield its total length. The outlines of cell bodies were also traced using the ‘contour’ function and the resulting surface areas were automatically calculated by the software.

### Multi-electrode array analysis

We used 12-well multi-electrode array plates from Axion Biosystems to acquire electrical activity reads from organoids. Ten organoids were plated onto each well at 20 days into the organoid derivation protocol, using Neurobasal medium containing GlutaMAX, 1% Gem21, 1% NEAA, 10 ng/mL of BDNF, 10 ng/mL of GDNF, 10 ng/mL of NT-3, 200 mM L-ascorbic acid and 1 mM dibutyryl-cAMP. They were maintained in this medium for 7 days, then switched to Neurobasal medium containing 1% GlutaMAX, 1% Gem21, 1% NEAA, and 0.5% penicillin/streptomycin for an additional 7 days. After this timeframe, the seeded organoids were kept in BrainPhys medium until the time of measurement. At least 2 independent experiments were conducted for each subject, with 3 independent replicates (wells) per subject in each experiment. Organoids were assessed for electrophysiological parameters starting 7 days after switching to BrainPhys medium. Data reported in Supplementary Fig. 5a were from organoids cultured for 30 days in BrainPhys medium. Data reported in Fig. 9d were from organoids cultured for up to 90 days in BrainPhys medium.

Recordings were performed using a Maestro system and the AxIS software (Axion Biosystems; version 1.0), with a bandwidth filter from 10 Hz to 2.5 kHz^41^. Spike detection was computed with an adaptive threshold of 5.5 times the standard deviation of the estimated noise for each electrode. Plates were left untouched in the Maestro instrument for 3 min prior to recording, which proceeded for 3 additional minutes. Data were analyzed using the Neural Metrics Tool (Axion Biosystems; version 2.5.1), under the condition that an electrode was deemed active if at least 5 spikes occurred over 1 min. The mean firing rate for a subject was calculated across active electrodes in all wells for that subject. Network bursts were defined as bursts of more than 10 spikes that occurred in more than 25% of the active electrodes in the well, with a maximum inter-spike interval of 100 ms.

### Patch-clamp electrophysiological analysis

Whole-cell patch-clamp recordings were performed on neurons in 2D (monolayer) culture from parent #4 and PTHS #4 individuals, differentiated from NPCs on 35 mm dishes coated with poly-ornithine and laminin for 4 months after withdrawal of FGF-2, as described above. Similar densities of cells were achieved in all plates, and cells were randomly selected in the dishes of control and PTHS groups. In total, 10 of 10 cells recorded in the parental control group and 9 of 10 cells recorded in the PTHS group exhibited action potential firings and voltage-dependent Na^+^ currents indicative of a classical neuronal identity. Therefore, the electrophysiological and immunostaining data (Fig. 4) together confirm that non-neuronal cells do not represent the majority of cells in the 2D cultures used for electrophysiological recordings, and that, therefore, no exclusion needed to be applied on recorded data. The extracellular solution used was 130 mM NaCl, 3 mM KCl, 1 mM CaCl_2_, 1 mM MgCl_2_, 10 mM HEPES, and 10 mM glucose, at pH 7.4 adjusted with 1 M NaOH (∼4 mM Na^+^ added). The internal solution for glass electrodes was 138 mM K-gluconate, 4 mM KCl, 10 mM Na_2_-phosphocreatine, 0.2 mM CaCl_2_, 10 mM HEPES (Na^+^ salt), 1 mM EGTA, 4 mM Mg-ATP, 0.3 mM Na-GTP, at pH 7.4 adjusted with 1 M KOH (∼3 mM K^+^ added). The osmolarity of all solutions was adjusted to 290 mOsm. Filamented borosilicate glass capillaries (1.2 mm OD, 0.69 mm ID, World Precision Instruments) were pulled on a Flaming/Brown micropipette puller (Model P-87, Sutter Instrument). The electrode resistances were 4–6 MΩ for the whole-cell recording. Axon CV-4 headstage and Axopatch 200 A amplifier (Molecular Devices) were used for the electrophysiological recordings at room temperature. For evoked AP recordings, current-clamp configuration was employed with the injection of small currents to maintain the membrane potential at -70mV. Then, voltage-clamp configuration was used to record voltage-dependent neuronal Na^+^ and K^+^ currents. Recordings were low-pass filtered at 1 kHz, and digitized at 10 kHz using a DigiData 1322 A (Molecular Devices). Liquid junction potentials were nulled. Electrophysiology data were analyzed offline using Axon pCLAMP 10 software (Molecular Devices; version 10.7). Statistical comparisons were performed with 10 (parent) or 9 (PTHS) neurons per group, using two-tailed Welch’s *t* test with a significance threshold of *p* = 0.05.

### Proliferation and apoptosis assays

For quantifying cell proliferation via cell counting, we seeded individual wells of a poly-ornithine/laminin-coated 12-well plate with 100,000 cells per well. At least 2 experiments were conducted per subject, with three technical replicates (wells) per subject per experiment. After the indicated number of days, cells were lifted by Accutase treatment for 5 min, resuspended in equal volumes of DMEM/F12 and counted using a Chemometec Via-1 cassette, which also calculated total live cell counts.

For EdU cell cycle assays, we used the Click-iT EdU Flow Cytometry Assay Kit (Thermo Fisher Scientific), following the manufacturer’s protocol. Briefly, 70% confluent NPCs from 10 cm dishes were dissociated with Accutase, resuspended in StemDiff Neural Progenitor Medium (Stem Cell Technologies) and plated onto Matrigel-coated 6-well plates at a density of 0.2 × 10^6^ cells/well. Cells were incubated at 37 °C and 5% CO_2_ for 12 h before EdU was added to the culture medium at a final concentration of 10 μM. Cells were incubated for another 2.5 h for EdU incorporation, and subsequently harvested by Accutase-mediated dissociation, resuspension in 3 mL of 1% BSA in 1× PBS, and pelleting at 500 × *g* for 5 min. Pellets were resuspended and incubated in the kit’s fixative solution for 15 min in the dark at 25 °C, followed by the addition of 3 mL of 1% BSA in 1× PBS to stop fixation. Next, NPCs were pelleted at 500 × *g* for 5 min, the supernatant was removed, and the pelleted cells were incubated for 15 min in 1× Click-iT saponin-based permeabilization and wash reagent. During incubation, the Click-iT reaction cocktail was prepared based on the manufacturer’s protocol, and then added to the samples, followed by homogenization and incubation for 30 min, protected from light. Cells were re-homogenized every 5 min and then washed in 3 mL of 1× Click-iT permeabilization and wash reagent, pelleted, and resuspended in the same solution, before nuclear staining in 1× PBS/0.1% Triton X-100/100 μg/mL RNase A solution containing 20 μg/mL propidium iodide. Immediately after nuclear staining, cells were transferred to ice and kept at 4 °C, protected from light, until analysis on a LSR Fortessa X-20 cell cytometer (BD Biosciences). Data were analyzed using the FlowJo software (FlowJo, LLC; version 10.8.1) and the following gating strategy (Supplementary Fig. 14a): cell events in a SSC-A versus FSC-A plot were gated to include events between 0 and 150,000 in the SSC-A dimension and between 20,000 and 220,000 in the FSC-A dimension; this population was further gated to exclude doublets and clumps by including events below 100,000 in the FSC-W dimension and then events below 120,000 in the SSC-W dimension. The final population was analyzed for Alexa Fluor 647 fluorescence (EdU labeling) and propidium iodide fluorescence with excitation at 482 nm to reveal DNA content. The EdU+ population was defined in a parental control sample and the same gates were used for all remaining samples under analysis. The EdU+ population corresponds to the S cell cycle population and was gated according to standard practice, following the manufacturer’s instructions, by selecting the arch of cells above 1000 in the APC-A (650 nm excitation) dimension. The effectiveness of the gating strategy was confirmed with negative controls not labeled with EdU, not labeled with propidium iodide, or not labeled with both (Supplementary Fig. 14b).

Apoptosis assays on NPCs were conducted using the Dead Cell Apoptosis Kit with Annexin V FITC and PI (V13242, Thermo Fisher Scientific), following the manufacturer’s protocol, and analysis by flow cytometry in the same instrument described above.

### TOP-Flash luciferase reporter Wnt functional assay

For assessing levels of Wnt signaling, 70% confluent cultures of NPCs in 24-well plates (with Neural Progenitor Medium; Stem Cell Technologies) were transfected with the M50 Super 8×TOPFlash plasmid (Addgene #12456; http://n2t.net/addgene:12456; RRID:Addgene_12456), which is used to assess β-catenin-mediated transcriptional activation. This plasmid contains a minimal TA viral promoter driving the expression of a firefly luciferase gene preceded by seven binding sites (AGATCAAAGG) for TCF/LEF^67^ (not to be confused with the *TCF4* mutated in PTHS). Control NPCs were transfected with the M51 Super8×FOPflash plasmid, which has mutant TCF/LEF binding sites (Addgene plasmid #12457; http://n2t.net/addgene:12457; RRID:Addgene_12457).

Transfection was performed using the Amaxa Mouse Neural Stem Cell Nucleofector kit for NPCs (Lonza), using the manufacturer’s recommendations. After 24 h, medium was replenished and the luciferase assay was performed using the Pierce Firefly Luciferase Flash Assay Kit (Thermo Fisher Scientific) on a sample of 50,000 cells, using a Synergy microplate reader (BioTek Instruments). All assays were conducted on three independent replicates per NPC line (per subject) and three technical replicates. Activity levels were expressed as arbitrary units normalized against the mean activity in the respective controls.

### Wnt signaling manipulation

For manipulating the Wnt/β-catenin signaling pathway in NPCs, we seeded 200,000 cells on a 6-well plate, followed by treatment with specific agonist CHIR99021 (1 μM) for 4 days. Controls were treated with DMSO (CHIR diluent) at the same concentration and for the same duration. The final concentration of DMSO in all experiments was 0.05% (v/v). In separate experiments, cells were treated with Wnt signaling antagonists DKK-1 (25 μM) or ICG-001 (1 μM) for 3 to 5 days. In all cases, treated cells were assayed to confirm modulation of the activity of the Wnt pathway, via transfection with the TOP-Flash plasmids described above. For all experiments, we used 3 biological replicates per subject line, and similar results were obtained in at least 3 independent experiments.

We chose a CHIR99021 concentration of 1 μM because higher concentrations resulted in toxic effects, particularly on the senescent PTHS NPCs. It should be noted that, while the effects of treatment with 1 μM CHIR99021 are pronounced in the PTHS lines, the same treatment does not increase proliferation of the parent NPC lines in 2D culture (Fig. 6h, i), probably because these cultures are maintained in a highly proliferative state due to the presence of FGF-2, and agonistic Wnt activation with 1 μM CHIR99021 is unable to further enhance proliferation.

For the quantification of TCF4 protein levels in CHIR0021-treated NPCs in 2D culture, we could not perform Western Blotting due to the inefficiency of our TCF4 antibody in detecting the SDS-denatured target protein at native levels in this type of experiment. Alternatively, we conducted fluorometric measurement of TCF4 immunostaining intensity (Supplementary Fig. 9f). Briefly, NPCs (3 replicate wells per subject/condition) were seeded onto 24-well plates at equivalent numbers and treated with DMSO (control) or CHIR99201 for 10 days. Cells were then washed with cold PBS and subjected to immunostaining as described in previous sections, using the following primary antibodies: rabbit anti-TCF4 antibody (Abcam; ab217668; 1:10,000) and mouse anti-β-actin (Abcam; ab6276; clone [AC-15]; 1:10,000). Cells were washed 4 times of 5 min each with 1× PBS/0.1% Triton X-100, followed by incubation for 2 h at 23-25 °C with the following Li-COR secondary antibodies (diluted 1:5,000): IRDye 680RD goat anti-rabbit IgG (H + L) (Li-COR; #926-68071) and IRDye 800CW donkey anti-mouse IgG (H + L) (Li-COR; #926-32212). After additional 4 washes of 5 min each with 1× PBS/0.1% Triton X-100, fluorescent detection and quantification was conducted on an Odyssey XF Scanning and Imaging System (Li-COR), using default parameters. For the presentation of TCF4 immunostaining intensity in Supplementary Fig. 9f, we normalized the TCF4 fluorescence levels by β-actin fluorescence in each well, followed by calculating the means among replicates for each subject.

We additionally conducted flow cytometry to determine TCF4 protein levels in NPCs in 2D culture after treatment with CHIR99021 (Supplementary Fig. 9g). NPCs (3 replicate wells per subject/condition) were seeded onto 6-well plates at equivalent numbers and treated with DMSO (control) or CHIR99201 for 10 days. Cells were then washed with cold PBS, dissociated with Accutase for 5 min, followed by inactivation with 5 volumes of DMEM/F12 and centrifugation to pellet the cells. The NPCs were then subjected to incubation for 20 min with the LIVE/DEAD Fixable Dead Cell Stain 367/526 nm kit (Thermo Fisher Scientific; L34965) to stain dead cells, following the manufacturer’s recommendations. Cells were then immediately fixed with 0.5% formaldehyde in 1× PBS for 15 min, pelleted, and permeabilized with 0.1% Triton X-100 in 1× PBS for 15 min. After centrifugation, cells were resuspended in 0.1% Triton X-100/1× PBS/2% BSA and incubated for 30 min for blocking. Next, the NPCs were incubated with anti-TCF4 primary antibody (Abcam; ab217668; diluted 1:100 in the same solution) for 1 h, with constant flicking. After 3 washes in 0.1% Triton X-100 in 1× PBS, cells were centrifuged and incubated with 0.1% Triton X-100/1× PBS/2% BSA containing anti-rabbit Alexa-488 secondary antibody (Thermo Fisher; A-11034; 1:500) for 30 min in the dark. After 3 washes in 0.1% Triton X-100 in 1× PBS, cells were centrifuged and resuspended in ice-cold PBS and kept on ice in the dark until flow cytometry analysis on a LSR Fortessa X-20 cell cytometer (BD Biosciences). Data were analyzed using FlowJo software (FlowJo, LLC; version 10.8.1) and the following gating strategy (Supplementary Fig. 14c): live cells were kept by selecting events below 1,000 in the BV510 dimension in a plot of LIVE/DEAD Fixable Dead Cell Stain kit fluorescence versus FSC-A, as per the kit’s recommendations; this population was further gated in a SSC-A versus FSC-A plot to include events between 0 and 100,000-150,000 in the SSC-A dimension and between 20,000 and 260,000 in the FSC-A dimension; this population was further gated to exclude doublets and clumps by including events below 100,000 in the FSC-W dimension and then events below 110,000 in the SSC-W dimension. The final population was analyzed for Alexa Fluor 488 fluorescence (TCF4 labeling) with excitation at 515 nm to reveal TCF4 expression levels. The TCF4+ population was defined in a parental control sample and the same gates were used for all remaining samples under analysis. The TCF4+ population was defined as cells containing fluorescence above 125.00 in the Alexa Fluor 488 dimension. The effectiveness of this gating strategy was confirmed with negative controls not labeled with anti-TCF4 primary antibody or not labeled with both primary and secondary antibodies (Supplementary Fig. 14d). An average fluorescence intensity was then calculated for each replicate and mean TCF4 fluorescence values were computed for all subjects, as presented in the bar plot in Supplementary Fig. 9g.

CtOs or GbOs were treated with 1 μM CHIR99021 (or DMSO, as a control) on the first day of the progenitor proliferation phase (when FGF-2 is first added to the growing organoids), in the same type of medium as untreated organoids. Similarly, treatment with Wnt antagonist ICG-001 (1 μM) was performed on the first day of the progenitor proliferation phase. In all cases, treatment was performed on at least 3 independent replicates of each organoid line.

### *TCF4*, *SOX3*, and *SOX4* knockdown

For *TCF4* and *SOX3* knockdown in NPCs, we transfected 100,000 cells using the Amaxa Mouse Neural Stem Cell Nucleofector kit (Lonza) with shRNA Mission plasmids (Sigma Millipore), using the manufacturer’s recommendations. We used the SHCLND-NM-005834 (*SOX3*) and SHCLND-NM_003199 (*TCF4*) pre-validated Mission shRNA vectors (Sigma Millipore), which are made in the pLKO.1 plasmid backbone (TRC2 series). SHC201 empty TRC2 vector was used as a control. Four days after transfection, cells were counted and RNA was extracted using the RNeasy Mini Plus kit (Qiagen), followed by analysis of gene expression for selected genes via RT-qPCR, as described above. Each experiment was conducted with three replicates per subject/condition for cell counting or three independent replicates for RNA extraction and gene-expression assessment. Because no selection was applied after transfection, the observed effects of *SOX3* or *TCF4* knockdown on the expression of other genes should be interpreted as the mean variation across all cells in the transfected population. This may explain why, for example, *TCF4* knockdown in NPCs leads to reduction in *SOX3* expression (Fig. 7c) with an effect size smaller than the one observed in the comparison between control and PTHS NPC samples (Fig. 7b; see Supplementary Data 1 for effect sizes).

For *SOX4* knockdown in neurons (Fig. 7l–n), we decided to avoid methods that require transfection of the differentiating neuronal culture, as this would result in phenotypic alterations, cell death, and changes in cell density. Therefore, we adopted antisense oligonucleotides (ASOs), two of which were used in combination in all experiments. ASOs were designed using the manufacturer’s design tool as antisense 16 nucleotides long locked nucleic acid (LNA) oligos (Qiagen), which are enriched with LNAs in the flanking regions but contain regular DNA nucleotides in a LNA-free central gap (GapmeRs). Each ASO was resuspended in 10 mM Tris pH 7.5/0.1 mM EDTA and used at 1 μM final concentration. Differentiating neuronal cultures were treated with ASOs on days 15, 20 and 25 after withdrawal of FGF-2 via direct application to the culture medium for unassisted uptake (gymnosis). Cultures were fixed or harvested for RNA extraction three days after the last treatment with ASOs.

### *SOX3* over-expression

For *SOX3* over-expression in NPCs (Supplementary Fig. 10i, j), we transfected 100,000 cells using the Amaxa Mouse Neural Stem Cell Nucleofector kit (Lonza) with 1.5 μg of pENTER-CMV-SOX3 plasmid (Vigene Biosciences; CH850241), in which the *SOX3* coding sequence is controlled by the cytomegalovirus (CMV) promoter. Four days after transfection, cells were counted and RNA was extracted using the RNeasy Mini Plus kit (Qiagen), followed by analysis of gene expression for selected genes via RT-qPCR, as described above. Each experiment was conducted with 3 replicates per subject/condition for cell counting or 3 independent replicates for RNA extraction.

### *TCF4* over-expression

We tested the effects of overexpressing *TCF4* by transfecting control and PTHS NPCs with a construct containing the *TCF4* coding sequence under the control of an artificial promoter (Fig. 9a and Supplementary Fig. 13a; see details below). Numerous alternative promoters exist in the *TCF4* locus, which give rise to different transcripts^1–4^. We chose to overexpress the TCF4-B transcript variant, because it is the most highly expressed in most tissues^4^ and is transcribed from the most active promoter in NPCs, according to our promoter usage analysis (Supplementary Fig. 12a). For control conditions, the TCF4-B coding sequence was placed under the control of the artificial minP promoter (AGAGGGTATATAATGGAAGCTCG ACTTCCAG). The *TCF4* OE constructs contain the TCF4-B coding sequence preceded by the minP promoter and by varying numbers (6 or 12) of μE5 TCF4 regulatory DNA-binding sites (CACCTG) separated by spacer sequences (CAAGAA). These constructs were prepared via PCR-based reactions to ligate Ultramer oligonucleotides (minP_TCF4, E-box-x6-minP_TCF4, or E-box-x12-minP_TCF4; Integrated DNA technologies) containing the artificial promoter to the TCF4-B coding sequence, which was separately amplified via RT-PCR from human brain cDNA (Promega) using primers TCF4B_cDNA Forward and TCF4B_cDNA Reverse (see Supplementary Data 6 for oligo sequences). The resulting PCR fragments were cloned into *EcoR*I and *Xho*I restriction sites of the pLenti-III-promoterless vector (Applied Biological Materials). NPCs were transfected with these plasmids using the protocol described above, followed by extraction of total RNA with the RNeasy Mini Plus kit (Qiagen) and RT-qPCR, as described above.

For organoid transduction experiments (Fig. 9 and Supplementary Fig. 13), we prepared lentiviral particles containing the constructs described above using the second-generation lentiviral production plasmids psPAX2 (Addgene #12260) and pMD2.G (Addgene #12259). Thirty 10 cm plates of 80% confluent HEK293T cells were transfected with 10 μg of *TCF4* OE (or control) plasmids per plate, the 2^nd^ Generation Packaging mix (ABM; LV003), and Lentifectin transfection reagent (ABM; G074), using the manufacturer’s recommendations. Two days after transfection, the supernatant from all plates was harvested and the viruses were purified by PEG precipitation using PEG-it Virus Precipitation Solution (Systems Biosciences; LV810A-1). Titer determination was achieved using the qPCR Lentiviral Titration kit (ABM; LV900). Equivalent AAV vectors of AAV9 serotype were ordered from a commercial source (Applied Biological Materials), containing the human growth hormone (hGH) terminator in each construct. All titers were determined to be >10^9^ IU (particles) per μL. Transduction of organoids (CtOs) was achieved by mixing 3.5 million dissociated iPSCs on the first day of organoid derivation (lentiviruses; Fig. 9b) or by adding AAV virus directly to the medium after the last day of the neural induction phase (Fig. 9e), with appropriate virus quantities to obtain a multiplicity of infection (MOI) of 5 for each type of vector.

### CRISPR-mediated trans-epigenetic correction of *TCF4* expression

First, we analyzed RNA sequencing libraries from PTHS and control NPCs to determine the transcriptional activity from the numerous alternative promoters of the human *TCF4* gene^4^. Promoter activity estimation was performed using the junction read counts approach described in Demircioğlu et al.^68^. Briefly, exon junction counts were obtained by mapping the RNA-seq reads onto the GRCh38.p13 genome assembly with the STAR aligner (version STAR 2.7.6a), using the GENCODE 34 primary annotation as a reference to determine exon coordinates. Next, the proActiv R package (version 0.99.0) was used to estimate promoter activity by counting the junction reads mapping to the first set of introns of each *TCF4* transcript, followed by normalization of the counts using the size factors approach and log-transformation of the data. This approach identified promoters upstream of exons 3b, 8a, and 10a as the most active in both parent and PTHS samples (Supplementary Fig. 12a), which were therefore chosen for CRISPR-mediated trans-epigenetic manipulation of expression of the endogenous *TCF4* locus.

At each promoter, we designed gRNAs based on sequences located between −100 and +50 from the corresponding transcriptional start site (TSS)^51^ (Supplementary Fig. 12a). For each promoter, we selected 3 sense and 2 antisense gRNAs based on the score generated by the computational tool designed by Hsu et al.^69^. As a control gRNA sequence, we selected a non-targeting scrambled sequence (see Supplementary Data 6 for gRNA sequences). gRNAs were validated by first inserting the corresponding sequences into the traditional CRISPR pSpCas9(BB)-2A-Puro plasmid (Addgene #48139; http://n2t.net/addgene:48139; RRID:Addgene_48139), followed by testing the efficiency of each gRNA to generate indels in pilot experiments. To this end, pSpCas9(BB)-2A-Puro was digested with *Bpi*I (Thermo Fisher Scientific). Each synthesized gRNA oligonucleotide pair was phosphorylated with T4 polynucleotide kinase (Promega) and annealed by incubation in a thermocycler under the following conditions: 30 min at 37 °C, 5 min at 95 °C, and ramp down to 25 °C at 5 °C per min. Phosphorylated oligonucleotide duplexes for each gRNA were then ligated to the digested plasmid by incubation at 25 °C for 1 h with T4 DNA ligase (Promega). Competent cells (Stbl3 *E. coli* bacterial strain; Thermo Fisher Scientific) were transformed with each ligation product and plasmid DNA was extracted from each clone with the PureYield Plasmid Miniprep System (Promega), followed by validation via Sanger sequencing with the hU6-F universal primer.

Next, HEK293T cells (ATCC) cultured in DMEM containing 10% FBS and 1% penicillin/streptomycin (70% confluency) were transfected with each gRNA plasmid using polyethylenimine (PEI; Sigma-Aldrich) at a ratio of 3:1 PEI/DNA (w/w), with 1 μg of DNA per mL of culture medium. Both PEI and DNA were diluted in Opti-MEM (Gibco) at a volume 1/20 of the total volume of culture medium, followed by incubation for 30 min before being applied directly on top of the cells. Transfection medium was replaced with culture medium 16 h after transfection. To confirm that the selected *TCF4* gRNA sequences indeed targeted the promoters upstream of exons 3b, 8a and 10a of the human *TCF4* gene, we performed T7 endonuclease I assays. Transformed cells were selected by replacing the transfection medium with culture medium supplemented with 1 μg/mL puromycin until all cells in the negative control died (~72 h). Genomic DNA was then extracted using the Illustra Blood Genomic Prep Minispin kit (GE), according to the manufacturer’s instructions. We used one designed primer pair flanking each gRNA’s target site in the genome (sequences in Supplementary Data 6) and performed end-point PCR with Q5 high-fidelity DNA polymerase (NEB). Amplicons were purified with the Wizard SV Gel and PCR Clean-Up System (Promega) and quantified using the Qubit DNA BR Assay Kit (Thermo Fisher Scientific). For the T7 endonuclease I assay, 300 ng of amplicons of each sample were incubated with 2 μL NEBuffer 2 and H_2_O (to a final volume of 19.5 μL) in a thermocycler, with the following cycling parameters: 5 min at 95 °C, ramp down to 85 °C at −2 °C per min, ramp down to 25 °C at −0.1 °C per min. After denaturation and gradual re-annealing to allow formation of DNA heteroduplexes, 5U of T7 endonuclease I (NEB) were added to the samples, which were incubated for 30 min at 37 °C. The products were run on a 1.5% agarose gel and the CRISPR-mediated efficiency for the creation of indels was estimated for each gRNA based on the ratio between the masses of undigested bands and digested fragments. Additionally, the amplicons were deep sequenced and the percentages of clones with indels were computed.

All gRNAs were also cloned into the pLentiSAMv2 plasmid (Addgene plasmid #75112; http://n2t.net/addgene:75112; RRID:Addgene_75112), harboring the gRNA sequence with MS2 loops at both the tetraloop and the stem loop 2 under the control of the U6 promoter, along with the dead Cas9 (dCas9) gene fused with the *VP64* gene under the control of the EF1α promoter. Cloning was performed in pLentiSAMv2 via digestion with *Esp*3I (Thermo Fisher Scientific). Next, lentiviral particles were prepared by transfecting HEK293T cells (ATCC) with suitable pLentiSAMv2 vectors carrying the tested gRNAs or the scrambled gRNA (control).

To evaluate the efficiency of the designed *TCF4* gRNA sequences at increasing the endogenous expression of the *TCF4* gene via trans-epigenetic activation, we transfected SH-SY5Y cells with the pLentiSAMv2 and pLentiMPHv2 (Addgene #89308; http://n2t.net/addgene:89308; RRID:Addgene_89308) plasmids, followed by RT-qPCR to verify the levels of *TCF4* transcripts. The pLentiMPHv2 vector contains the MS2-P65-HSF1 activator helper (MPH) complex gene under the control of the EF1α promoter^51^, which is needed, in combination with the gRNA and dead Cas9, for the trans-epigenetic activation of the *TCF4* locus. SH-SY5Y cells were cultured in DMEM/F12 containing 10% FBS and 1% penicillin/streptomycin, and then transfected with FuGENE HD Transfection Reagent (Promega) at a ratio of 4:1 FuGENE/DNA (v/w), with 2 μg of DNA per mL of culture medium. Both FuGENE and DNA were diluted in Opti-MEM (Gibco) at a volume 1/10 of the total volume of culture medium, without an incubation period before being applied to the cells. Transfection medium was replaced with culture medium supplemented with 10 μg/mL blasticidine S (Sigma-Aldrich) 16 h later, for selection of transfected cells. After control cells died (~72 h), selection medium was replaced with culture medium to allow cells to expand. For each gRNA, transfection was performed in triplicates. RNA from selected cells was purified with TRIzol reagent (Thermo Fisher Scientific), according to the manufacturer’s instructions. cDNA was synthesized with ImProm-II Reverse Transcription System (Promega). For RT-qPCR reactions, we designed primer pairs able to detect (I) transcripts encoding TCF4-B, TCF4-D, or TCF4-A (depending on the corresponding promoter targeted by each gRNA), (II) transcripts of the endogenous *TBP* gene, and the exogenous dCas9 (encoded by lentiSAMv2) and MPH (encoded by lentiMPHv2) genes, and (III) transcripts from genes transcriptionally regulated by TCF4. See Supplementary Data 6 for a complete list of oligonucleotides used. All reactions were performed with technical duplicates, using the PowerUp SYBR Green Master Mix (Applied Biosystems) on a QuantStudio 6 Flex Real-Time PCR System (Applied Biosystems). A melt-curve step was always included at the end of each run. Samples transfected with an empty lentiSAMv2 plasmid or with a plasmid containing the scrambled gRNA were used as references for the quantification of relative levels of *TCF4* and TCF4-target gene transcripts via the traditional ΔΔCt method, with transcript levels of *TBP*, dCas9 and MPH used for normalization between samples.

For organoid transduction experiments (Fig. 8 and Supplementary Fig. 12), we prepared lentiviral particles from pLentiMPHv2 vector and the several pLentiSAMv2 versions containing different gRNAs. For virus preparation, the second-generation lentiviral production plasmids psPAX2 (Addgene #12260) and pMD2.G (Addgene #12259) were used. Twenty 10 cm plates of 80% confluent HEK293T cells were transfected with 10 μg of plasmid per plate, the 2nd Generation Packaging mix (ABM; LV003), and Lentifectin transfection reagent (ABM; G074), using the manufacturer’s recommendations. Two days after transfection, the supernatant from all plates was harvested and the viruses were purified by PEG precipitation using PEG-it Virus Precipitation Solution (Systems Biosciences; LV810A-1). Titer determination was achieved using the qPCR Lentiviral Titration kit (ABM; LV900). All titers were determined to be above 10^9^ IU (particles) per μL.

Transduction of organoids (CtOs) was achieved by mixing 2.5 million dissociated iPSCs on the first day of organoid derivation with the appropriate virus quantities to obtain a multiplicity of infection (MOI) of 5 for each type of lentivirus. When using the regular organoid derivation protocol, the addition of two lentiviral vectors on the first day led to slightly impaired cellular aggregation. This forced us to alternatively use micro-wells for these trans-epigenetic *TCF4* correction experiments. This was achieved by placing the mixture of dissociated iPSCs and viruses onto a well of an Aggrewell micro-well plate (Stem Cell Technologies; #34411) on the first day of the protocol. Cells were left undisturbed, without shaking, for 16 h inside the Aggrewell. During this period, iPSCs collected at the bottom of the Aggrewell and formed very homogeneous embryoid bodies inside the micro-wells. On the following day, embryoid bodies were carefully dislodged with the aid of a tissue culture pipettor and transferred to 6-well plates. From this point onwards, organoids were cultured under agitation on a shaker, following the same regimen applied to the regular derivation protocol. Organoids in the ‘scrambled gRNA’ condition (control) were co-transduced with lentiviruses produced from pLentiMPHv2 and pLentiSAMv2 plasmids containing a scrambled gRNA. Organoids in the ‘*TCF4* gRNA’ group were co-transduced with lentiviruses produced from pLentiMPHv2 and pLentiSAMv2 plasmids containing gRNA version 3bS3. On the second and third days of organoid derivation, medium was replaced and lentiviruses were added again. During these 3 days, embryoid bodies were formed in the presence of mTeSR1 Plus medium containing **SB431542** and dorsomorphin, as described above. From the fourth day onward, medium was replaced as per the regular protocol, without the addition of viruses. Transduction was confirmed by the evaluation of Cas9 expression via immunostaining, using the protocol described above. It should be noted that the use of Aggrewell on the first day of the organoid derivation protocol led to organoids with fewer rosettes in the middle and clustered progenitors at the organoid’s periphery.

### Statistical analyses and reproducibility

Data are presented as mean + SEM, unless otherwise indicated. We did not use statistical methods such as power analysis to determine sample size, because we were restricted by the PTHS samples available, which were chosen based on availability of detailed information about the types of *TCF4* mutation carried by each patient. However, based on the strong and consistent effect sizes observed throughout the study (Supplementary Data 1) and on the level of variability across cell lines from all subjects (in NPCs and organoids), further power analysis determined that increasing sample size is not expected to change statistical significance of our results.

Different types of statistical test were used throughout the study, as indicated in the corresponding figure legends. Usually, comparisons of means between two groups (PTHS against parent) in experiments that measured organoid size, relative expression levels, or expression abundances used two-sample Welch’s *t* test, assuming unequal variances and heteroskedasticity. When comparing these types of means among more than two groups, we used one-way Analysis of Variance (ANOVA), followed by Tukey’s Honestly Significantly Different (HSD) *post-hoc* test. For comparing mean gene expression in single cell RNA-Seq data between two samples, we used the non-parametric Mann–Whitney *U* test. For the same type of comparison among more than two samples, we used the Kruskal–Wallis test, followed by Dunn’s *post-hoc* test. For comparisons of mean gene-expression values in single-cell transcriptomic data, we presented the calculated *p*-values as asterisks in the figures but added a cross symbol (†) to those comparisons in which the log2 fold change between PTHS and parents was smaller than an arbitrary value of 0.5 (equivalent to a gene-expression variation of approximately 40% compared to the parent group mean), in either direction. For comparing neurite length and soma area between PTHS and control neurons, we used one-way ANOVA with Geisser-Greenhouse correction for repeated measures, followed by Fisher’s Least Significantly Different (LSD) *post-hoc* test. For comparing the expression of *SOX4* along the differentiation trajectory pseudotime (Supplementary Fig. 11i), we used the Kolmogorov-Smirnov two-sample distribution test.

Sample sizes are indicated in the figure legends and in Supplementary Data 1. *P-*values are reported as asterisks in the figures for significance levels defined as *p* < 0.05 (*), *p* < 0.01 (**), or *p* < 0.001 (***). Supplementary Data 1 presents extended results for all statistical tests performed, including sample sizes, statistical tests employed, effect sizes, statistics metrics (*H, F, t*, or *W*), along with exact *p*-values, listed according to the order of appearance in figure panels throughout the study. When experimentation involved more than one independent replicate per subject cell line, or more than one technical replicate per independent replicate, the numbers of replicates are also indicated in the figure legends and Supplementary Data 1, even though each statistical test was run based solely on the comparison between the means of different subjects. All attempts at replication were successful. Experiments conducted with 4 subjects always included samples from PTHS #1 to #4, and experiments conducted with 3 subjects always included samples from PTHS #1, #2 and #4, along with the respective parental controls, and no randomization was applied because we were restricted by the limited number of patients with PTHS included in the study (*N* = 5) and by the different types of *TCF4* mutation each subject carries. When experiments involved data collected from independent biological replicates, results were collected from randomly chosen replicates (batches in organoid preparation or wells/plates of experiments involving cells in 2D culture). For each batch, organoids were randomly selected from each well for data collection. Blinding was used for most analyses comparing patients and control samples, including immunostaining, measurement of organoid size, cell counting, patch-clamp electrophysiological measurements, and multi-electrode array assays. Blinding was not used when analyzing results from RNA sequencing and single cell RNA sequencing experiments, due to the inherently unbiased nature of the bioinformatic approaches used for quantitating gene-expression and determining differential expression between genotypes or cell types. Microscopy images that appear in Figs. 1e, g, j, 2a, c, d, f, 5b, g, j, k, m, 6f, l, 7h, l, 8b, c, h, and 9b, e (bottom panel) and in Supplementary Figs. 1b, e, f, 2b, f, j, 4j, 5f, 6c–e, 9i, 12g, i, j, and 13b,e are representative images from three experiments which were repeated independently with similar results.

Statistical analyses were performed using Prism software (GraphPad; version 9.2.0), RStudio (version 1.4.1106), G*Power (version 3.1), and WebPower (version 2018). Figures were composed with Prism and Illustrator CS4 (Adobe; version 14.0.0).

### Reporting summary

Further information on research design is available in the Nature Research Reporting Summary linked to this article.

## Supplementary information

Supplementary Information

Description of Additional Supplementary Files

Supplementary Data 1

Supplementary Data 2

Supplementary Data 3

Supplementary Data 4

Supplementary Data 5

Supplementary Data 6

Reporting Summary

## Source data

Source Data

## Data Availability

Data supporting the findings in this study are included within the Supplementary Material. The source data relevant to Figs. 1–9 and Supplementary Figs. 1–13 are provided as a Source Data file. RNA sequencing and single cell RNA sequencing raw and processed data generated during this study were deposited at the Gene Expression Omnibus (GEO) of the National Center for Biotechnology Information (NCBI), under accession numbers GSE159392, GSE159859, GSE159860, and GSE189121, which are available publicly without restriction. The following public databases have been used in this study and can be accessed via the corresponding weblinks in parentheses: GRCh38-2020-A (10x) (support.10xgenomics.com/single-cell-gene-expression/software/release-notes/build#GRCh38_2020A); GENCODE release 32 (www.gencodegenes.org/human/release_32.html); and GENCODE release 34 (www.gencodegenes.org/human/release_34.html). Large tables containing data related to gene-expression and differential expression analyses of bulk RNA sequencing experiments and data related to differential expression analysis of single-cell RNA sequencing experiments (Supplementary Data 2 and 3) are publicly available without restriction from the Zenodo repository (10.5281/zenodo.6325406)^70^. Microscopy images obtained during this study were not deposited in public repositories as they contain human patient sensitive information, but requests for these data will be fulfilled by the corresponding authors upon reasonable request following appropriate procedures of the Ethics Committees of the institutions where the patient biological samples and cells were collected or are maintained. Source data are provided with this paper.

## Code availability

No new custom software or code has been used in this paper. Codes (R programming language) used for bioinformatic analyses are all strictly based on pre-existing, regularly used published codes for RNA-Seq and single cell RNA-Seq analyses, as described above. These codes are available at https://github.com/apcamargo/2020-pths-rna-seq-analysis (Zenodo repository 10.5281/zenodo.6323774)^71^, along with examples of their use with our dataset and validation data, to ensure reproducibility.

## Materials availability

Further information and requests for resources and reagents should be directed to and will be fulfilled by the corresponding authors, Fabio Papes (fpapes@ucsd.edu; papesf@unicamp.br) and Alysson Muotri (muotri@ucsd.edu), upon reasonable request. When provided to others, unique reagents generated in this study will be available with a completed Materials Transfer Agreement.
