# Author Correction: Transcription Factor 4 loss-of-function is associated with deficits in progenitor proliferation and cortical neuron content

**DOI:** 10.1038/s41467-025-56808-8

**Published:** 2025-02-12

**Authors:** Fabio Papes, Antonio P. Camargo, Janaina S. de Souza, Vinicius M. A. Carvalho, Ryan A. Szeto, Erin LaMontagne, José R. Teixeira, Simoni H. Avansini, Sandra M. Sánchez-Sánchez, Thiago S. Nakahara, Carolina N. Santo, Wei Wu, Hang Yao, Barbara M. P. Araújo, Paulo E. N. F. Velho, Gabriel G. Haddad, Alysson R. Muotri

**Affiliations:** 1 Department of Genetics, Evolution, Microbiology and Immunology, Institute of Biology, University of Campinas, Campinas, Sao Paulo 13083-862 Brazil; 2 Department of Pediatrics, School of Medicine, University of California San Diego, La Jolla, CA 92093 USA; 3 Center for Medicinal Chemistry, University of Campinas, Campinas, Sao Paulo 13083-886 Brazil; 4 Graduate Program in Genetics and Molecular Biology, Institute of Biology, University of Campinas, Campinas, Sao Paulo 13083-862 Brazil; 5 Lawrence Berkeley National Laboratory, Berkeley, CA 94720 USA; 6 School of Medical Sciences, University of Campinas, Campinas, Sao Paulo 13083-887 Brazil; 7 Department of Neurosciences, School of Medicine, University of California San Diego, La Jolla, CA 92093 USA; 8 Rady Children’s Hospital, San Diego, CA 92123 USA; 9 Department of Cellular & Molecular Medicine, School of Medicine, University of California San Diego, La Jolla, CA 92093 USA; 10 Kavli Institute for Brain and Mind, University of California San Diego, La Jolla, CA 92093 USA; 11 Center for Academic Research and Training in Anthropogeny (CARTA) and Archealization (ArchC), University of California San Diego, La Jolla, CA 92093 USA

Correction to: *Nature Communications* 10.1038/s41467-022-29942-w, published online 02 May 2022

In the version of the article initially published, the text “UCSD has filed a patent application (WO2022072709A1), in which F.P. and A.R.M. are inventors, containing some results regarding the TCF4 correction overexpression strategy described in this paper. The patent was published on 04-07-2022” was missing from the Competing interests section and has now been added to the HTML and PDF versions of the article.

