# Supplementary material for: Transcription Factor 4 loss-of-function is associated with deficits in progenitor proliferation and cortical neuron content
Source: Nat Commun. 2022 May 2;13:2387. doi: 10.1038/s41467-022-29942-w (PMC9061776; doi:10.1038/s41467-022-29942-w)
Supplement: Supplementary file 1 — Supplementary Information [file 41467_2022_29942_MOESM1_ESM.pdf]

Supplementary Figures and Tables for

Transcription Factor 4 loss-of-function is associated with deficits in progenitor proliferation and cortical neuron content

Authors: Fabio Papes*, Antonio P. Camargo, Janaina S. de Souza, Vinicius M.A. Carvalho, Ryan A. Szeto, Erin LaMontagne, José R. Teixeira, Simoni H. Avansini, Sandra M. Sánchez-Sánchez, Thiago S. Nakahara, Carolina N. Santo, Wei Wu, Hang Yao, Barbara M.P. Araújo, Paulo E.N.F. Velho, Gabriel Haddad, and Alysson R. Muotri*

Journal: *Nature Communications*

Supplementary Figure 1

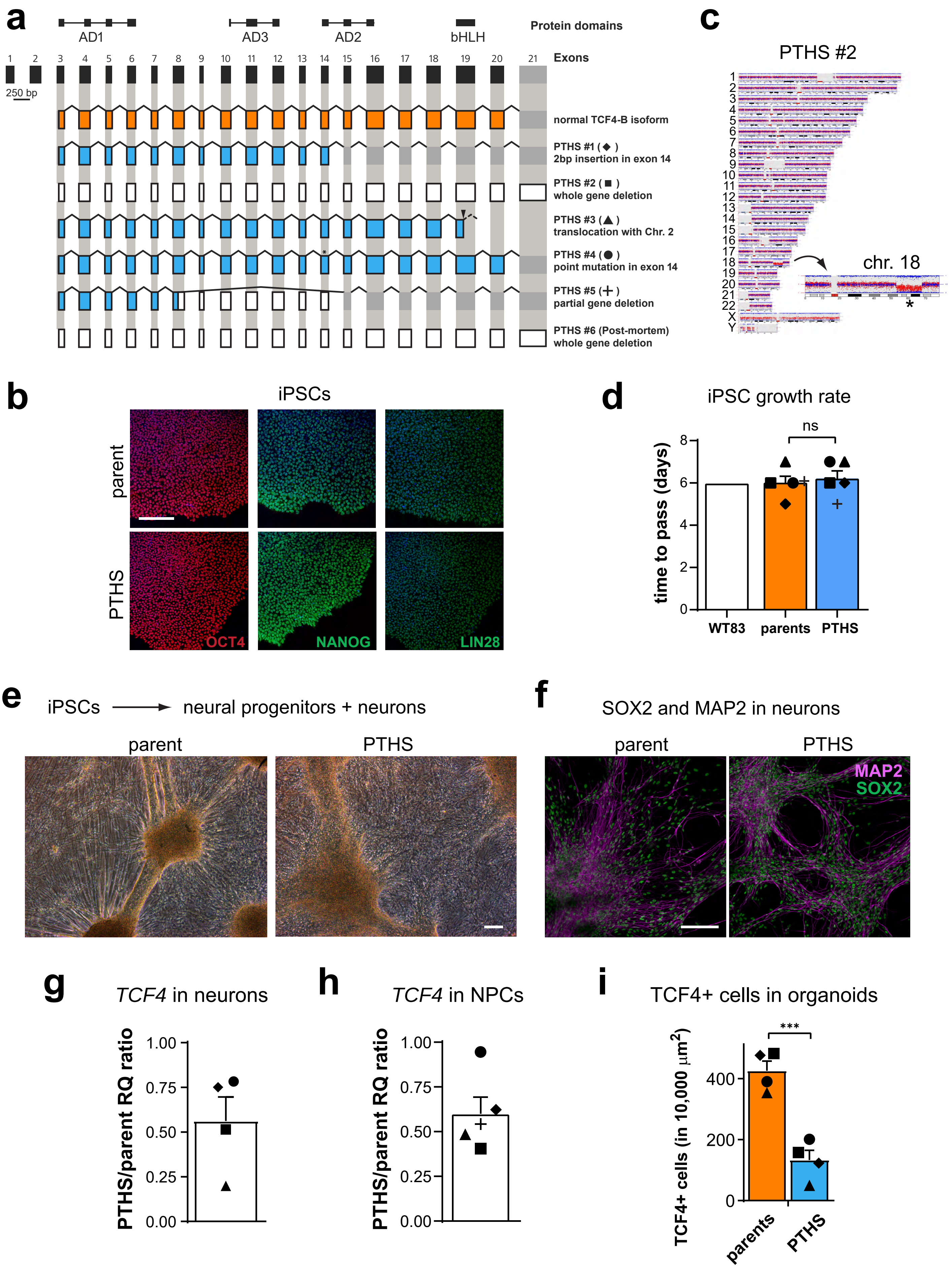

Supplementary Fig. 1: PTHS iPSCs exhibit normal growth rate and can be differentiated into neurons.

a, Structure of the *TCF4* locus (exon numbers on top of each rectangle) in different patients. White rectangles symbolize missing exons due to partial or whole gene deletion. Rectangles with thick borders represent the coding sequence in each case. AD1 to AD3: transcriptional activation domains; bHLH: basic helix-loop-helix DNA-binding domain. Exons 1 and 2 are shown but they are not part of the main transcript for the *TCF4* gene, called TCF4-B. Details on the types of mutation carried by each patient are given on the right (see also Supplementary Table 1 for further information). Note that, in all patients expect PTHS #4, the mutated allele yields no protein or a truncated protein without the essential DNA-binding domain. **b**, Example of immunostaining of parent and PTHS iPSC colonies for pluripotency markers OCT4, NANOG, and LIN28. **c**, Example of digital karyotyping by SNP mapping via chip hybridization, on sample from patient PTHS #2 (Supplementary Table 1), showing large deletion on chromosome 18 (asterisk). Numbers on the left represent chromosomes. The *y* axis in each chromosome graph represents the logR ratio for individual hybridized chip probes (dots). **d**, Comparison of iPSC colony growth rate (in days to reach size required for passaging, which is 2 mm) between parents (controls), PTHS, and a control iPSC line derived from a subject not involved in the study (WT83). N = 5 subjects per group (symbols). Measurement for each subject is the median from 5 independent plates. **e**, Representative bright-field microscopy image showing iPSC-derived neurons in culture (2 months in neuronal medium). Notice the formation of islands of progenitors and neuronal cell bodies connected by bundles of neuronal fibers in both the parent and PTHS groups. **f**, Fluorescence microscopy images of cultures in **e** stained for MAP2 (magenta) and SOX2 (green), evidencing general ability of iPSCs to differentiate into NPCs (SOX2+) and neurons (MAP2+) in both groups. **g**, Ratio of relative quantitation of *TCF4* expression measured via RT-qPCR (RQ) between iPSC-derived PTHS and parent neuronal cultures (3 months in neuronal medium). N = 4 subjects per group (symbols), 3 independent replicates per subject, 2 technical replicates per sample. **h**, Ratio of relative quantitation of *TCF4* expression measured via RT-qPCR (RQ) between PTHS and parent in NPCs in 2D culture. N = 5 subjects per group (symbols), 3 independent replicates per subject, 2 technical replicates per sample. Notice that for one PTHS subject (PTHS #4, circle symbol) the expression is not diminished, concordant with the expectation for this patient, whose mis-sense point mutation is not predicted to affect transcript abundance. All other patients have mutations expected to decrease transcript content (non-sense mutation, frameshift mutation, whole gene deletion, or translocation). **i**, Numbers of cells immunostained for TCF4 in a 100 × 100 μm

square, in parent and PTHS CtOs, at 4 weeks in vitro. N = 4 subjects per group (symbols), 6 sections per subject, 4 ROIs per section. Symbols in bar graphs indicate parent-patient identities: diamonds, pair #1; squares, pair #2; triangles, pair #3; circles, pair #4; crosses, pair #5. Colors in bar graphs and gene model represent parents (orange) or PTHS (blue) groups. Bar graphs represent mean + SEM. n.s., not statistically significantly different, ***$p<0.001$; two-sample Welch's t test (**d,i**). Scale bars are 100 μm. See Supplementary Data 1 for statistical test results, including sample sizes, numbers of replicates, exact *p*-values, and effect sizes.

Supplementary Figure 2

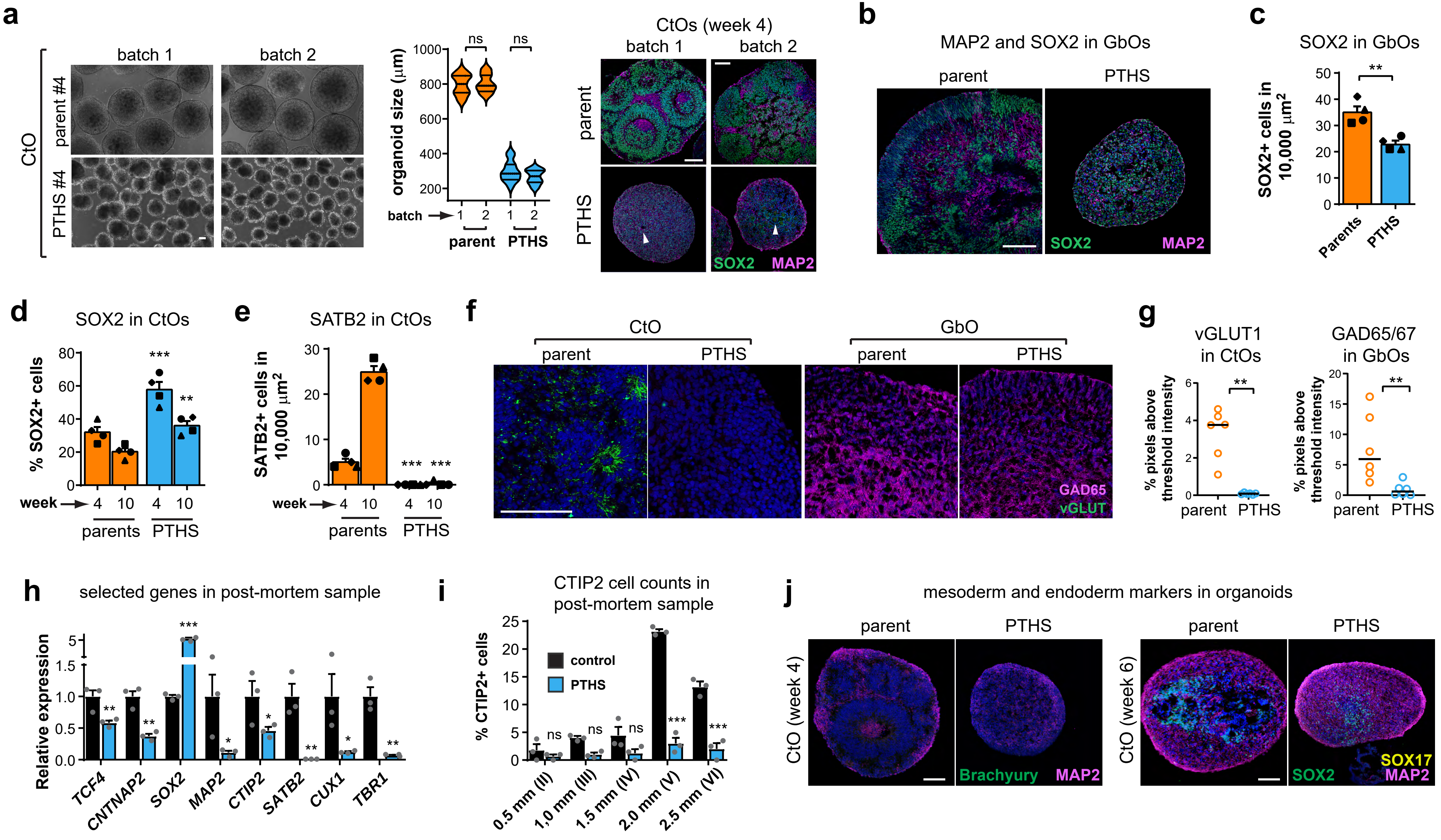

Supplementary Fig. 2: PTHS neural tissue exhibits severe diminishment of neuronal content.

a, Left: Bright-field images of PTHS and parental control CtOs at 4 weeks in vitro, derived from iPSCs of two different batches (clones of parent-patient pair #4). Middle: Organoid size distribution for parent (orange) and patient (blue) clones, evidencing high reproducibility across clones. The horizontal lines in violin plots represent the median, upper, and lower quartile. N = 12 organoids per group. Right: Immunostaining for SOX2 (green) and MAP2 (magenta) in different clones of PTHS and parental control CtOs at 4 weeks in vitro, exemplifying reproducibility in organoid structure and cellular composition. Arrowheads indicate small rosettes in the patient clones. **b**, Fluorescence microscopy images of parent and PTHS GbOs after immunostaining for NPC marker SOX2 (green) and neuronal marker MAP2 (magenta), at 6 weeks in vitro. **c**, Quantification of the density of SOX2+ cells in parent and PTHS GbOs at 6 weeks in vitro. N = 4 subjects (symbols), 2 independent experiments (replicates) per subject, 6 organoids per subject in each replicate, 4 random 100 × 100 μm ROIs per organoid. **d**, Percentage of SOX2+ cells (cell count over DAPI+ cells) in CtOs of parent and PTHS subjects at 4 and 10 weeks in vitro. N = 4 subjects (symbols), 3 independent experiments (replicates), 6 organoids per subject in each replicate, 4 random 100 x 100 μm regions of interest (ROIs) per organoid. **e**, Quantification of the density of cortical neurons expressing SATB2 in parent and PTHS CtOs at two stages of development in vitro. N = 4 subjects (symbols), 3 independent experiments (replicates) per subject, 6 organoids per subject per replicate, 4 random ROIs per organoid. **f**, Reduced staining for glutamatergic neuron marker vGLUT1 (green fluorescence) in PTHS CtOs and for GABAergic markers GAD65/67 (magenta) in PTHS GbOs at 8 weeks in vitro. **g**, Quantification of vGLUT1 and GAD65/67 expression, as judged from number of pixels above threshold intensity per unit area, in PTHS and control CtOs and GbOs at 8 weeks in vitro (see Methods for details). N = 6 sections per group (circles), 4 ROIs per section, with organoids from parent-pair #4. **h**, Relative expression of neural markers in post-mortem PTHS cortex sample (PTHS #6), in comparison with control brain sample. N = 3 independent replicates per group, 4 technical replicates. **i**, Quantification of percentages of CTIP2+ cells in post-mortem sample at different depths in the cortex (in mm). N = 3 sections per group at each depth, 4 random ROIs per section. Approximate cortical layers in the parent group are indicated in parentheses. **j**, Fluorescence microscopy images of CtOs at 4 weeks in vitro after staining for mesoderm marker Brachyury and MAP2 (left), and endoderm marker SOX17, MAP2 and SOX2 (right). Symbols in bar graphs indicate parent-patient identities: diamonds, pair #1; squares, pair

#2; triangles, pair #3; circles, pair #4; gray dots, post-mortem samples. Colors in bar graphs, violin and dot plots represent parents (orange), PTHS (blue), or control post-mortem sample (black) groups. Bar graphs represent mean + SEM. n.s., not statistically significantly different, *$p<0.05$, **$p<0.01$, ***$p<0.001$; two-sample Welch's t test (**c**,**h**), one-way ANOVA followed by Tukey-Kramer's HSD post-hoc test (**a**,**d**,**e**,**i**), or two-sided Wilcoxon-Mann-Whitney U test (**g**). Scale bars are 100 μm. See Supplementary Data 1 for statistical test results, including sample sizes, numbers of replicates, exact *p*-values, and effect sizes.

Supplementary Figure 3

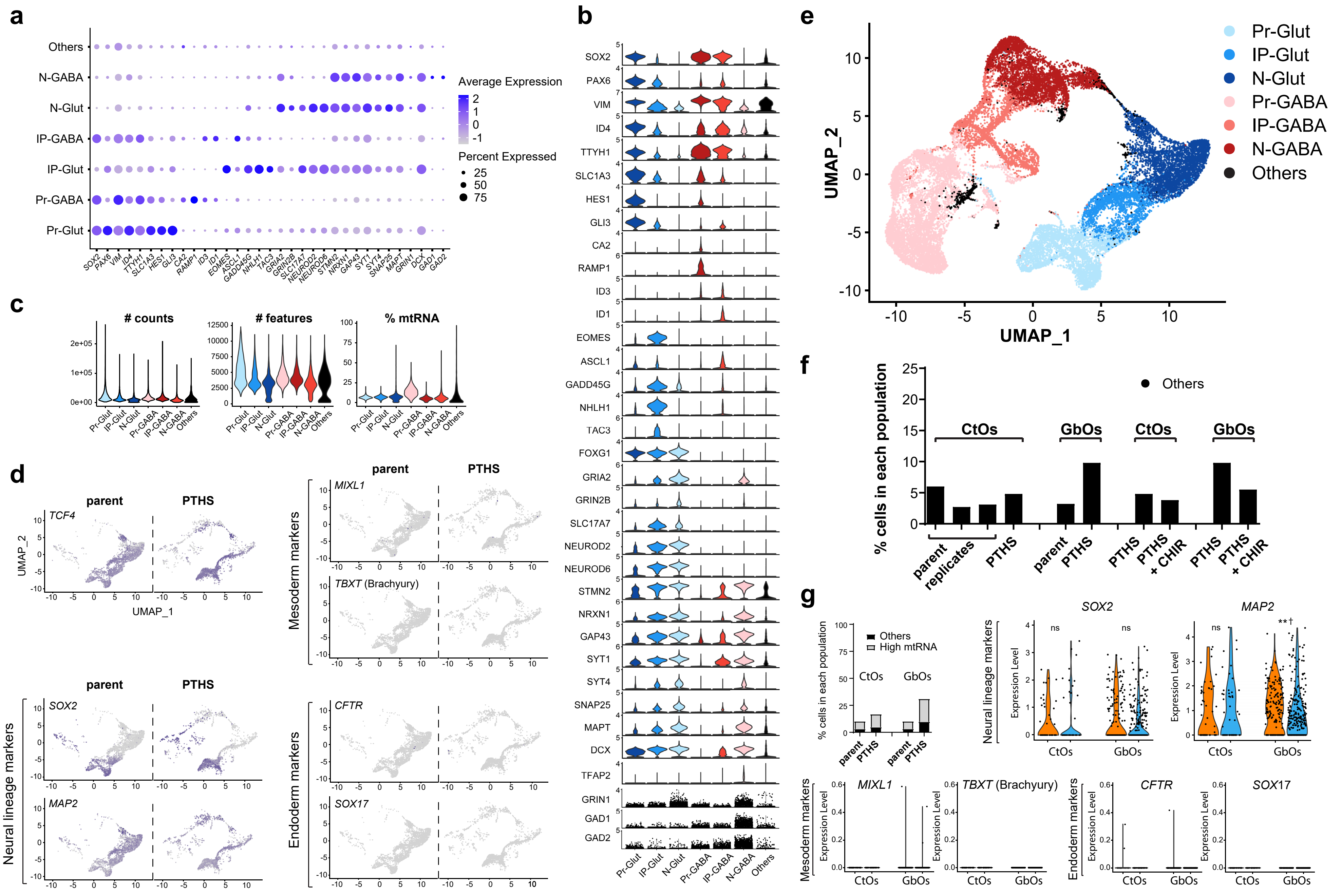

Supplementary Fig. 3: Annotation of subpopulations in single cell RNA-Seq experiments and associated controls.

a, Dot plot showing expression of selected marker genes (see Methods for marker gene selection and validation) in the six subpopulations of cells analyzed in the single cell transcriptomic data of PTHS and parent CtOs and GbOs, as depicted in Fig. 3a. Pr-Glut: neural progenitor cells in glutamatergic lineage; IP-Glut: intermediate progenitors in glutamatergic lineage; N-Glut: glutamatergic neurons; Pr-GABA: neural progenitors in inhibitory lineage; IP-GABA: intermediate progenitors in inhibitory lineage; N-GABA: neuronal population containing GABAergic interneurons. 'Others' represents a minor and heterogeneous group of cells not included in the previous six categories (see panel **e** for analysis of this population and Methods for details). Dot sizes are the percentages of cells in each subpopulation that have detectable expression for the corresponding gene. Dot color intensity represents average gene expression. **b**, Violin plots for marker genes shown in **a**, displaying range of expression in the six analyzed subpopulations of cells and in the 'Others' category. Color code is the same as in Fig. 3a. For *GRIN2*, *GAD1*, and *GAD2*, the medians were low and therefore the expression in each cell is represented as a dot. **c**, Single cell RNA-Seq quality control data. Violin plots represent the numbers of read counts, numbers of detected genes (features), and percentages of expressed mitochondrial genes (mtRNA) in the subpopulations listed in **a**. Color code is the same as in Fig. 3a and 'Others' group is shown in black. **d**, UMAP embedding of single cell transcriptomic data showing expression of *TCF4,* neural lineage markers *SOX2* and *MAP2*, mesoderm markers *MIXL1* and *TBXT* (Brachyury), and endoderm markers *CFTR* and *SOX17* in PTHS and parent CtOs. Intensity of purple indicates relative expression level. **e**, UMAP embedding of scRNA-Seq profiling of CtOs and GbOs at 8 weeks in vitro, integrating data from all eight libraries (see Methods for details), to evidence the position of cells in the 'Others' category. Color code is the same as in Fig. 3a and 'Others' category is shown in black. **f**, Percentages of cells in 'Others' category in the 8 scRNA-Seq libraries. Comparisons are separated into 4 groups: CtOs, GbOs, PTHS CtOs treated with CHIR99021, and PTHS GbOs treated with CHIR99021 (see data with CHIR-treated organoids below). Notice that cells in the 'Others' category are rare in all libraries. The replicate libraries of parent CtOs are shown in the order 'replicate #3', 'replicate #1', and 'replicate #2', to highlight the comparison between parent CtO replicate #2 and PTHS CtO libraries used elsewhere in the figures. **g**, Top left: Percentages of cells in 'Others' category and in a group of cells exhibiting high mitochondrial RNA content (which was not included in the analyses), in parent and PTHS CtOs and GbOs. Parent CtO bar indicates data from replicate #2, as an example. Top right: Expression levels of neural lineage markers *SOX2* and *MAP2* in

the ‘Others’ category of CtOs and GbOs in parent (orange) and PTHS (blue). Bottom: Expression levels of the same mesoderm and endoderm markers shown in **d,** in the ‘Others’ category of CtOs and GbOs of parent (left in each group) and PTHS (right in each group) genotypes. All single cell transcriptomic data in the figure were obtained with parent #4 and PTHS #4 CtOs and GbOs that have been maintained for 8 weeks in vitro. n.s., not statistically significantly different, ***p*<0.01, two-sided Wilcoxon-Mann-Whitney U test (**g**). The cross symbol indicates that the means of gene expression on a per cell basis was statistically significantly different between the parent and PTHS groups, but the log2 fold change between PTHS and parents was smaller than an arbitrary value of 0.5 (equivalent to a variation of approximately 40% compared to the parent group mean). See Supplementary Data 1 for statistical test results, including sample sizes, numbers of replicates, exact *p*-values, and effect sizes.

Supplementary Figure 4

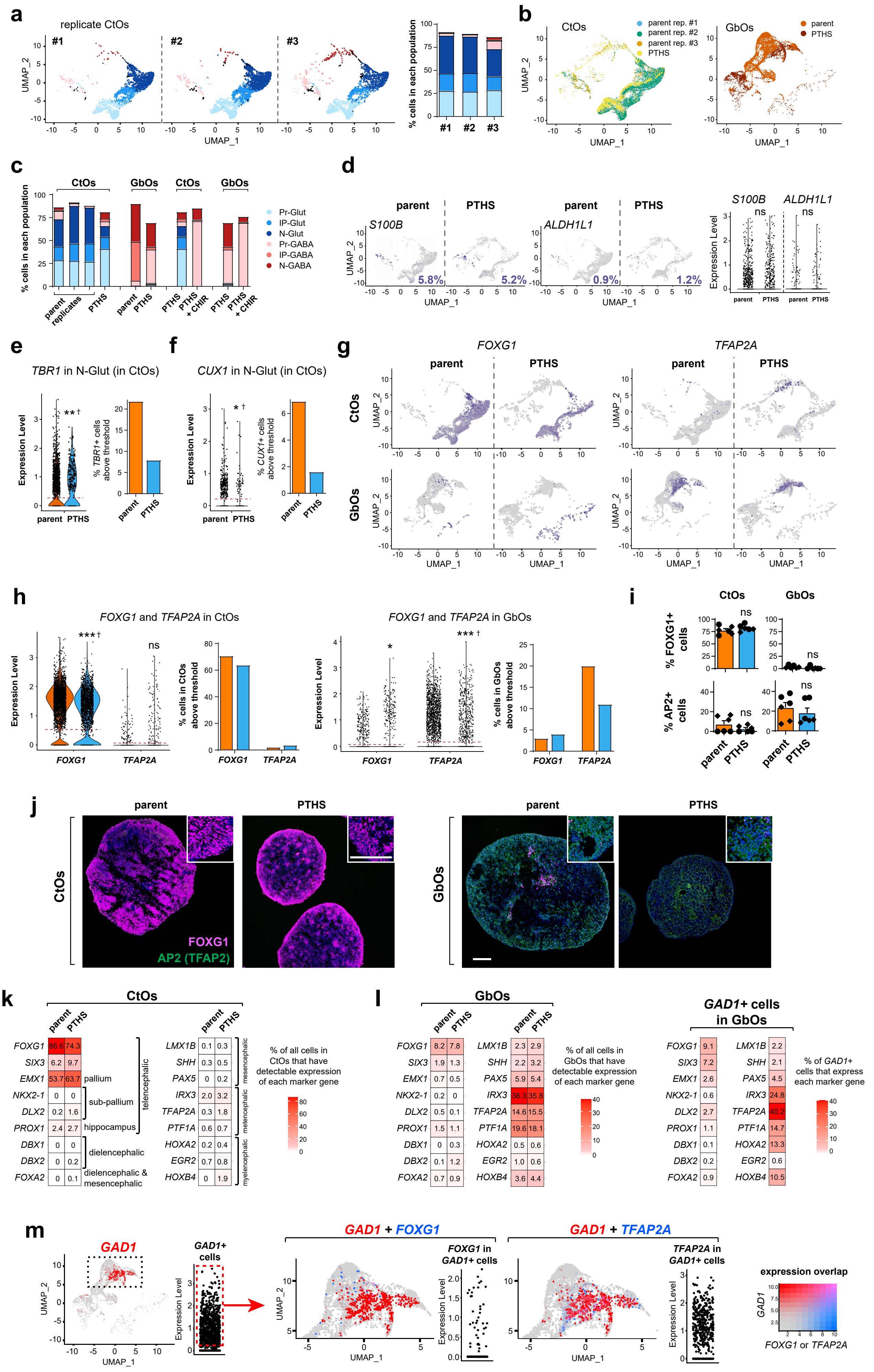

Supplementary Fig. 4: Controls for single cell RNA-Seq investigation of parent and PTHS organoids.

a, Left: Controls showing reproducibility in the generation of CtOs derived from the same genotype in independent experiments (replicates #1, #2, and #3 of parent CtO libraries). Right: Percentages of cells in each of the six subpopulations analyzed in replicate single cell RNA-Seq libraries, showing robustness of scRNA-Seq analyses, which detect equivalent percentages of each cell type in independent batches of organoids. Color code is the same as in Fig. 3a. 'Others' cells are shown in black in UMAP embeddings. **b**, Left: UMAP embedding showing data from 3 replicate libraries of parent CtOs and one library of PTHS CtO, color-coded according to sample of origin, to show absence of segregation of transcriptomic profiles according to sample and absence of gross differences in transcriptomic landscape between parent and PTHS CtOs. Right: UMAP embedding showing data from parent and PTHS GbO libraries, color-coded according to sample of origin, to show absence of gross differences in transcriptomic landscape between parent and PTHS GbOs. **c**, Percentages of cells in each of the six subpopulations analyzed in all 8 single cell RNA-Seq libraries. Comparisons are separated into 4 groups: CtOs, GbOs, PTHS CtOs treated with CHIR99021, and PTHS GbOs treated with CHIR99021 (see data with CHIR-treated organoids below). Color code is the same as in Fig. 3a. See also Supplementary Table 2 for exact percentage numbers for each group and for cells in 'Others' minority category, as well as cells with high mtRNA expression (which were not included in the analyses). The replicate libraries of parent CtOs are shown in the order 'replicate #3', 'replicate #1', and 'replicate #2', to highlight the comparison between parent CtO replicate #2 and PTHS CtO libraries used elsewhere in the figures. **d**, Left: UMAP showing expression of astrocytic markers *S100B* and *ALDH1L1* in parent and PTHS CtOs. Purple numbers indicate the percentages of cells expressing each marker gene at any detectable level in each genotype. Right: Expression level comparison for the same genes in parent and PTHS CtOs. N = 3544 (parent) or 2944 (PTHS) cells. **e,f**, Left: Comparison between parent and PTHS CtOs in terms of expression for genes coding for markers of cortical neuronal subtypes *TBR1* (**e**) and *CUX* (**f**) in the N-Glut subpopulation. N = 1401 (parent) or 380 (PTHS) cells. Right: Severe reduction in percentages of neuronal subtypes in PTHS CtOs (cells expressing each marker gene above threshold corresponding to 40% of the respective mean, which is shown by the red dashed line). **g**, UMAPs showing expression of *FOXG1* (telencephalic marker) or *TFAP2A* (expressed in some non-telencephalic lineage cells) in parent and PTHS CtOs and GbOs. The purple color indicates the expression level for each marker gene at any detectable level in each condition. Notice the predominant expression of *FOXG1* in CtOs and *TFAP2A* expression in the

GABAergic lineage. **h**, Left: Violin plots showing comparison between parent and PTHS CtOs in terms of *FOXG1* and *TFAP2A* expression. N = 3544 (parent) or 2944 (PTHS) cells. Bar plots show percentages of cells in CtOs expressing each gene above threshold corresponding to 40% of the respective mean (shown by the red dashed line in the corresponding violin plots). Notice the predominance of *FOXG1*+ cells in CtOs of either group. Right: Violin plots show comparison between parent and PTHS GbOs in terms of *FOXG1* and *TFAP2A* expression. N = 6466 (parent) or 3792 (PTHS) cells. Bar plots show percentages of cells in GbOs expressing each gene above threshold (red dashed line in corresponding violin plots). Notice that *TFAP2A*+ is expressed in a fraction of GbO cells of either genotype. **i**, Quantification of FOXG1+ and AP2+ cells in parent and PTHS CtOs and GbOs. High magnification images are shown in insets. N = 6 sections per group; 3 sections per subject; 3 ROIs per section. **j**, Representative fluorescence microscopy images of CtOs and GbOs after immunostaining for FOXG1 and AP2 (TFAP2A). **k**, Heatmaps showing percentages of all cells in CtOs that express each neural lineage marker gene at detectable levels. Genes are divided in telencephalic, dielencephalic, mesencephalic, metencephalic, and myelencephalic categories. The numbers inside each square denote the exact percentages of cells in parent and PTHS libraries expressing each marker. **l**, Left: Heatmaps showing percentages of all cells in GbOs that express each neural lineage marker gene. The numbers inside each square denote the exact percentages of cells in parent and PTHS libraries. Right: Heatmaps showing percentages of *GAD1*+ cells (GABAergic neurons) in GbOs that express each marker gene. Similar results were obtained with *GAD2*+ cells. **m**, Examples of expression analysis conducted on the *GAD1*+ population in GbOs in **l**. The red cells in the UMAP plot and the dashed red rectangle in the violin plot on the left represent the *GAD1*+ population analyzed. The levels of overlap between *GAD1* expression (red) and expression of *FOXG1* or *TFAP2A* (blue) are shown as examples on the right. The colormap indicates extent of overlap, and the violin plots indicate *GAD1*+ cells that also express *FOXG1* or *TFAP2A*. All single cell transcriptomic data in the figure were obtained with parent #4 and PTHS #4 CtOs and GbOs that have been maintained for 8 weeks in vitro. Bar graphs represent mean + SEM in **i**. n.s., not statistically significantly different, *$p<0.05$, **$p<0.01$, ***$p<0.001$, two-tailed Wilcoxon-Mann-Whitney U test (**d-f,h**) or two-tailed Welch's t-test assuming unequal variances (**i**). Colors in bar graphs and violin plots in **d-f,h,i** represent parents (orange) or PTHS (blue) groups. The cross symbol indicates that the means of gene expression on a per cell basis was statistically significantly different between the parent and PTHS groups, but the log2 fold change between PTHS and parents was smaller than an arbitrary value of 0.5 (equivalent to a variation of approximately 40% compared to the parent group mean). Scale bar

is 100 μm. See Supplementary Data 1 for statistical test results, including sample sizes, numbers of replicates, exact *p*-values, and effect sizes.

Supplementary Figure 5

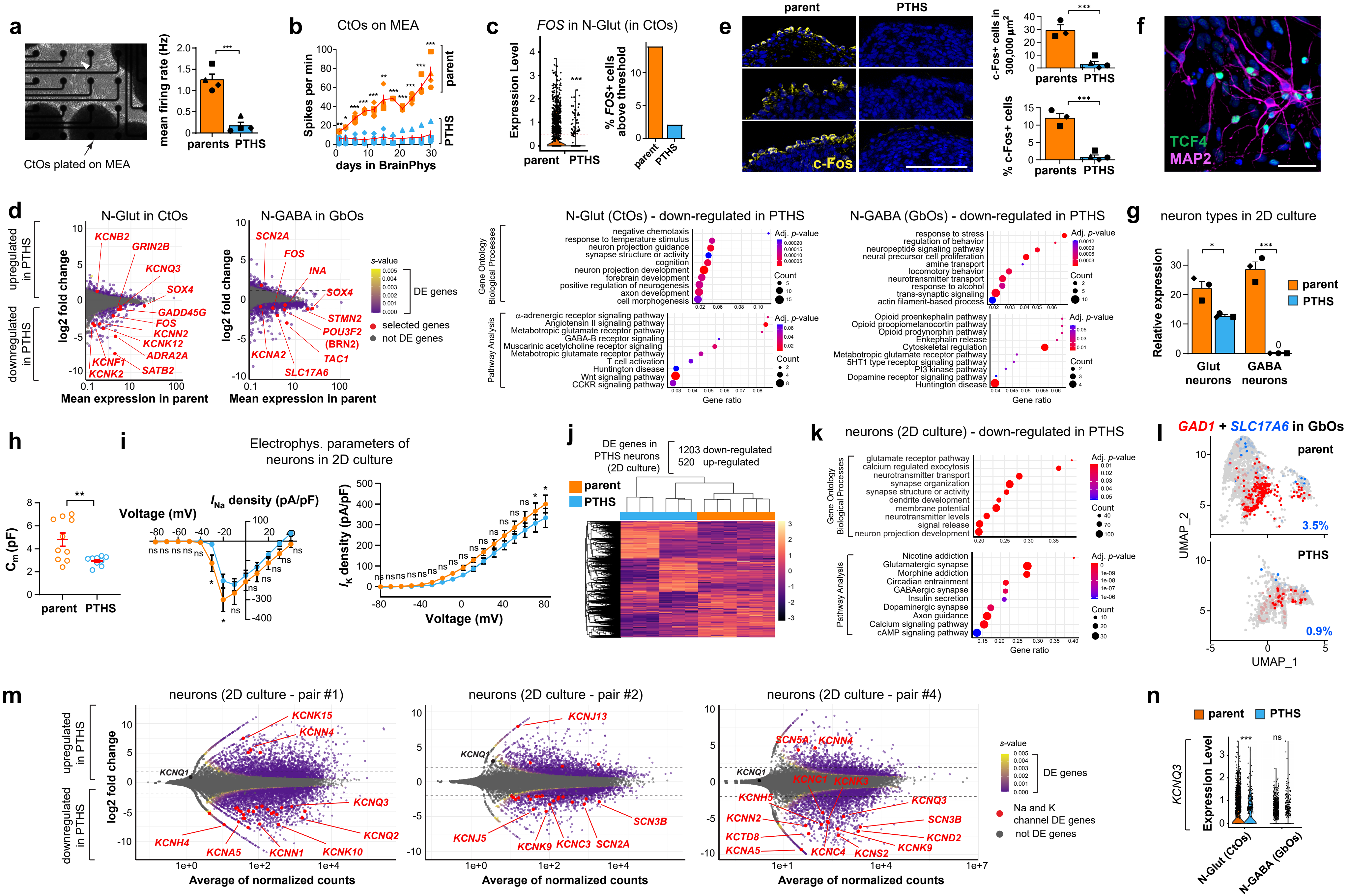

Supplementary Fig. 5: Supporting data for functional investigation of neurons in 2D culture and in organoids.

a, Left: CtOs seeded on multi-electrode array (MEA) plate. Right: Mean firing rate in active electrodes in MEA evaluation of CtOs. N = 4 subjects per group (symbols), 2 independent experiments per subject, 3 replicates (wells) per subject in each experiment, 10 organoids per subject in each replicate. Recordings were performed on organoids at 8 weeks in vitro. Arrowhead indicates neuronal processes on top of electrodes. **b**, Time course of mean firing rate in CtOs from 1 to 2 months in vitro, subjected to multi-electrode array (MEA) assay, showing comparison between parent (orange) and PTHS (blue) organoids. Each patient is represented with a different symbol (as in Supplementary Table 1). The red lines and error bars represent the mean across all subjects over time. N = 4 subjects (symbols), 6 independent replicates per subject, 10 organoids seeded per well in each replicate. **c**, Left: Expression of *FOS* in N-Glut neurons in parent and PTHS CtOs at 8 weeks in vitro. Violin plots represent distribution of gene expression in each population and each dot represents expression in a single cell. N = 1401 (parent) and 380 (PTHS) cells. Right: Percentages of *FOS*+ cells (expression above threshold indicated by red dashed line on the violin plot). Data were computed from scRNA-Seq data from parent-patient pair #4. **d**, DE genes in N-Glut and N-GABA neurons of CtOs and GbOs, comparing parent and PTHS organoids, computed from scRNA-Seq data from parent-patient pair #4. Left: MA plot showing DE genes. Each dot represents a gene, gray color indicates genes that are not DE between parent and PTHS groups, and the yellow-to-purple gradient represents DE genes, color-coded according to *s*-value. The *x*-axis represents average gene expression in the parent group. log2FC in *y*-axis indicates fold-change (in $\log_2$ scale). Genes with average expression below 0.1 in the parent group are not shown, since their expression is small and most are not DE. Selected DE genes described in the study are highlighted in red. Right: Dot plots representing results of Gene Set Enrichment Analysis followed by GO analysis (Biological Processes and Pathways) over DE genes that are down-regulated in PTHS N-Glut neurons in CtOs (left) or down-regulated in PTHS N-GABA neurons in GbOs (right). For each analysis, the top 10 categories in terms of adjusted *p*-value are shown. Dot size represents number of DE genes that fall into each classification category, dot color is the adjusted *p*-value, and the *x*-axis represents the percentage of genes in each category that are DE expressed genes in the scRNA-Seq libraries. Data were computed from scRNA-Seq data from parent-patient pair #4. **e**, Left: Fluorescence microscopy images of parent and PTHS CtOs at 6-8 weeks in vitro after immunostaining for indirect surrogate marker of neuronal activation c-Fos (yellow). Analysis of c-Fos protein

expression is important to confirm the decreased activity in PTHS organoids and to rule out the possibility that the expression of *FOS* gene in organoids is a consequence of the cellular dissociation applied prior to the generation of scRNA-Seq libraries. Right: Quantification of numbers of c-Fos+ cells in an area of 300,000 μm^2 and percentages of c-Fos+ cells in parent and PTHS CtOs (see Methods for a description of the ROIs used). Notice that c-Fos+ cells are concentrated at the organoid's periphery, as previously shown[1]. N = 3 parent and 5 PTHS samples (symbols), 5 sections per subject. **f**, Representative fluorescence microscopy image showing expression of TCF4 protein (green) in 2D cultures of neurons (MAP2 labeling shown in magenta) being differentiated from parent-derived iPSCs (1 month in neuronal medium). **g**, Percentages of glutamatergic (N-Glut) and GABAergic (N-GABA) neurons in 2D cultures of parent and PTHS neurons, as judged from deconvolution of bulk RNA-Seq data assisted with markers identified from the analysis of subpopulations in scRNA-Seq data (see Methods for details). These data suggest a lower percentage of N-Glut and absence of N-GABA neurons in the PTHS cultures. N = 3 subjects per groups; 3 replicate RNA-Seq libraries per subject. **h**, Comparison of membrane capacitance between PTHS (blue circles) and parent (orange circles) cells via patch-clamp electrophysiological analysis in 2D cultures of parent-pair #4 neurons (3 months in neuronal medium). N = 10 (parent) or 9 (PTHS) neurons. **i**, Interrogation of sodium (top) and potassium (bottom) current densities, comparing PTHS (blue line) and parent (orange line) neurons in 2D culture for parent-pair #4 (3 months in neuronal medium). N = 10 (parent) or 9 (PTHS) neurons. **j**, Heat map showing the expression levels for the 20,000 most highly expressed genes in RNA-Seq libraries of neurons in 2D culture (3 months in neuronal medium) from 2 parents and respective PTHS children (PTHS #1 and #4). The numbers of differentially expressed (DE) genes in each parent-child comparison and the number of common DE genes across all 3 parent-child pairs are shown above the plot (see Supplementary Data 2 for list of DE genes). **k**, Dot plot results for Gene Ontology – Biological Processes (top) and Pathway analysis (bottom), for down-regulated DE genes common to all parent-patient comparisons in **j**. For each analysis, the top 10 categories in terms of adjusted *p*-value are shown. Dot size represents number of DE genes that fall into each classification category, dot color is the adjusted *p*-value, and the *x*-axis represents the percentage of genes in each category that are DE expressed genes in the RNA-Seq libraries. Notice the presence of categories related to glutamatergic and GABAergic transmission. **l**, UMAP embeddings showing cells that express *GAD1* (red) and rare cells that express *SLC17A6* (vGLUT2) (blue) within the N-GABA subpopulation of GbOs. The blue numbers on the lower corner indicate the percentages of *SLC17A6*+ cells in parent and PTHS organoids. **m**, MA plot for genes expressed in control and

PTHS neurons in 2D culture. Each parent-patient comparison is shown separately. Gray dots are genes that are not statistically significantly differentially expressed between control and PTHS. Colored dots are statistically significant DE genes, and the color indicates adjusted *p*-value. Red dots represent genes coding for sodium or potassium channels with a $\log_2$ fold change superior to 2 in each direction (dashed lines). The *x* axes represent average normalized expression across all libraries (‘baseMean’). For clarity purposes, only DE channel genes with the largest fold-changes are labeled. See Supplementary Data 2 for full list of DE channel-coding genes. **n**, Expression levels of potassium channel *KCNQ3* in neurons of parent and PTHS organoids (N-Glut neurons of CtOs and N-GABA neurons of GbOs). N = 1401 (parent) or 380 (PTHS) N-Glut cells, or N = 2661 (parent) and 988 (PTHS) N-GABA cells. Data were computed from scRNA-Seq obtained from parent-patient pair #4. Symbols in bar graphs indicate parent-patient identities: diamonds, pair #1; squares, pair #2; triangles, pair #3; circles, pair #4. Colors in the bar graphs and line and violin plots represent parents (orange) or PTHS (blue) groups. Bar graphs represent mean + SEM. ns, not statistically significantly different, *$p<0.05$, **$p<0.01$, ***$p<0.001$; two-tailed Welch’s t-test (**a,e,h**), two-tailed multiple t-test with Holm-Sidak multiple comparison post-hoc test (**b**), two-tailed Wilcoxon-Mann-Whitney U test (**c,n**), or one-way ANOVA followed by HSD post-hoc test (**i**). Scale bars are 100 μm in (**e**) and 50 μm in (**f**). See Supplementary Data 1 for statistical test results, including sample sizes, numbers of replicates, exact *p*-values, and effect sizes.

Supplementary Figure 6

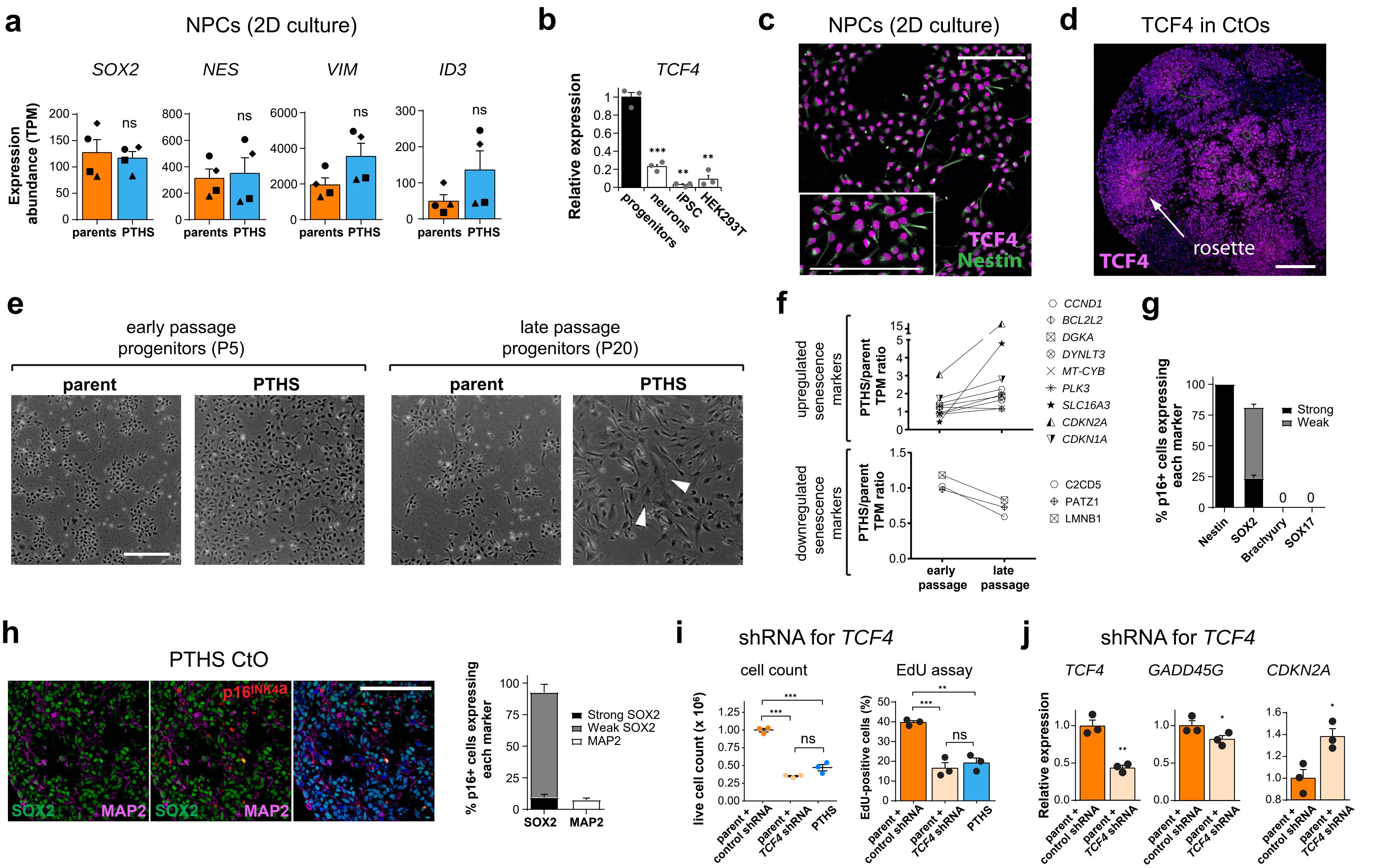

Supplementary Fig. 6: Phenotypic analyses of neural progenitor cells.

a, Expression of several NPC markers in iPSC-derived neural progenitor cells in 2D culture from parents (orange) and PTHS (blue) subjects, as judged from TPM expression abundance in RNA-Seq libraries. N = 4 subjects per group (symbols), 3 independent replicate libraries per subject. **b**, Relative *TCF4* expression levels (RT-qPCR) in iPSCs and iPSC-derived NPCs and neuronal cultures, as well expression in a non-neural cell line (HEK293T). All cells are of normal genotype; iPSCs and iPSC-derived cells are from a control subject not belonging to the PTHS study cohort (WT83). N = 3 independent replicates per group, 2 technical replicates per sample. Mean expression in neural progenitor group was normalized to 1. **c**, Representative fluorescence microscopy image of NPCs in 2D culture after immunostaining for TCF4 (magenta) and NPC marker Nestin (green). Higher magnification image in inset. **d**, Representative fluorescence microscopy image showing abundant expression of TCF4 (magenta) in NPCs of rosettes in control (parent #4) CtOs at 4 weeks in vitro. **e**, Representative images of control and PTHS NPCs in 2D culture at early and late passages. Notice the appearance of large flat cells in PTHS NPCs at passage 20 (P20) (arrowheads). **f**, Ratio between the expression of transcriptomic markers of replicative senescence between PTHS and control samples of NPCs in 2D culture, at early and late passages. For each gene, the mean at each passage was determined from 3 independent biological samples. Each line connects expression for a certain gene at early and late passage conditions. Marker genes are separated according to class (down-regulated or up-regulated in senescent cells, as in Hernandez-Segura et al.[2]). Notice the more pronounced mis-regulation in late passage conditions. Cells are from parent-child pair #4 and similar results were obtained for parent-child pairs #1 to #3 (Supplementary Data 2). **g**, Quantification of the percentages of cells expressing senescence marker $p16^{INK4a}$ that also express neural lineage marker Nestin, progenitor marker SOX2, mesoderm marker Brachyury, or endoderm marker SOX17, in parent and PTHS organoids. For SOX2, some cells exhibit strong staining (black color) and others are weakly stained (gray). See Methods for details on counting of SOX2 cells and colocalization with senescence marker. N = 4 subjects, 6 organoids per subject (from 3 independent experiments), 4 random ROIs per organoid. **h**, Left: High magnification images of CtOs at 4 weeks in vitro, stained for SOX2, MAP2 and $p16^{INK4a}$, followed by quantification of cells co-expressing $p16^{INK4a}$ with SOX2 or MAP2 (right). For SOX2, some cells exhibit strong staining (black color) and others are weakly stained (gray). N = 4 subjects, 6 organoids per subject (from 3 independent experiments), 4 random ROIs per organoid. **i**, Left: shRNA-mediated *TCF4* knockdown reduces proliferation of NPCs in 2D culture. N = 3 independent replicates (circles) per group, 3 technical replicates per sample.

Initial seeding density is 1×10^5 cells. Right: Quantification of percentages of EdU+ NPCs in 2D culture, in the same groups as on the left. N = 3 independent replicates per group (circles). Cells were from parent-patient pair #4. **j**, shRNA-mediated *TCF4* knockdown in NPCs in 2D culture leads to decreased expression of *TCF4* and TCF4 downstream target gene *GADD45G,* as well as increased expression of senescence marker *CDKN2A*. N = 3 independent replicates per group (circles), 2 technical replicates per sample. Cells were from parent-patient pair #4. Symbols in bar graphs indicate parent-patient identities: diamonds, pair #1; squares, pair #2; triangles, pair #3; circles, pair #4. Colors in bar and XY graphs represent parents (orange) or PTHS (blue) groups. Gray dots in **b** represent independent replicates of cells of normal genotype. Bar graphs represent mean + SEM. ns, not statistically significantly different, *$p<0.05$, **$p<0.01$, ***$p<0.001$; two-sample Welch's t-test assuming unequal variances (**a,j**) or one-way ANOVA followed by Tukey-Kramer's HSD post-hoc test (**b,i**). In **j**, mean gene expression was normalized to 1 in each parent + control shRNA group. Scale bars are 100 μm. See Supplementary Data 1 for statistical test results, including sample sizes, numbers of replicates, exact *p*-values, and effect sizes.

Supplementary Figure 7

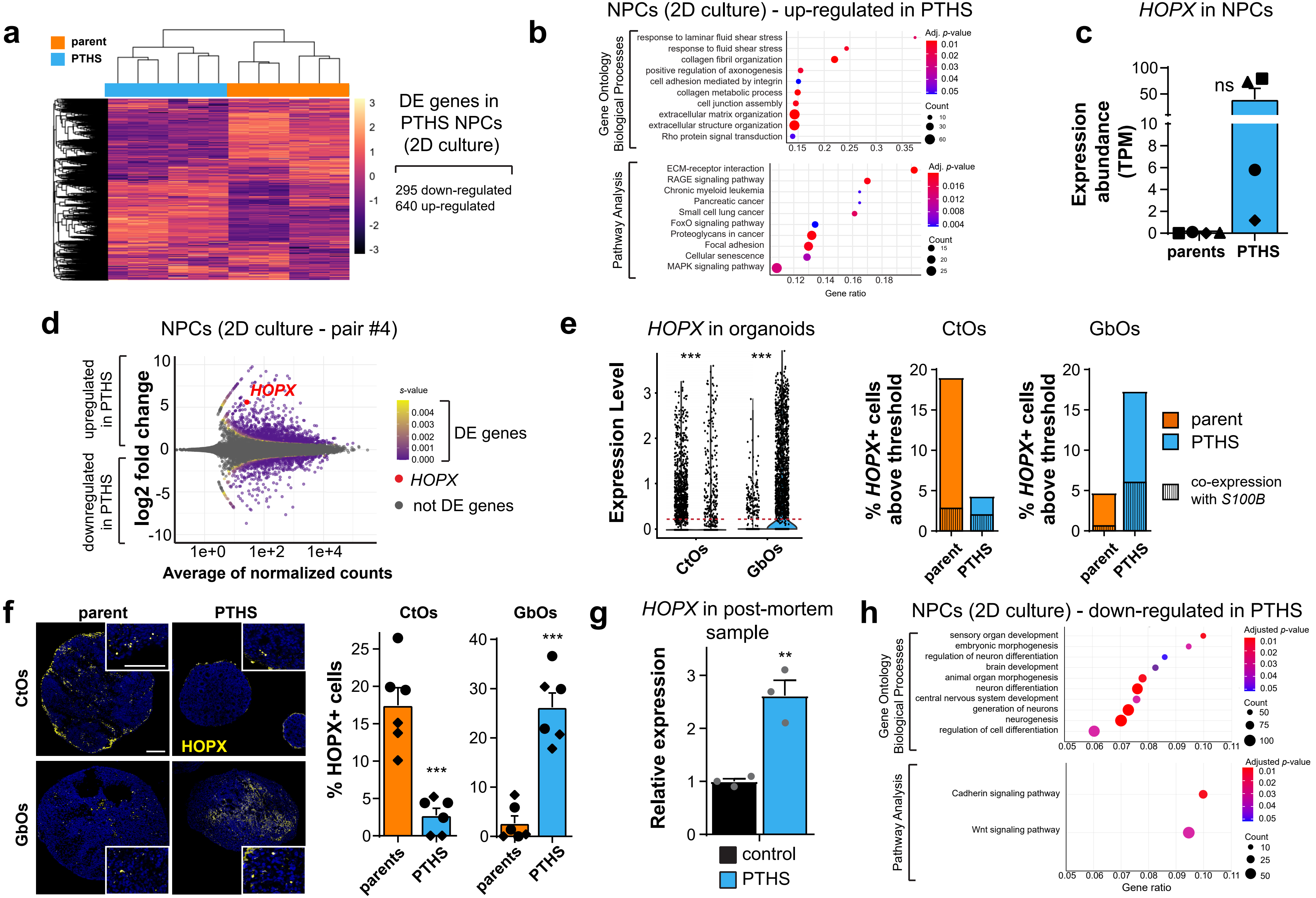

Supplementary Fig. 7: Differential gene expression analysis in neural progenitor cells.

a, Heat map showing the expression levels for the 20,000 most highly expressed genes in RNA-Seq libraries of NPCs in 2D culture from parents #1 and #4 and respective PTHS children, as an example (see Supplementary Data 2 for expression abundances in all 4 parent-child pairs and independent replicates). Three independent RNA-Seq libraries were sequenced per subject (columns in heatmap). The numbers of DE genes in the intersection among comparisons for all four parent-child pairs are shown above the plot (see Supplementary Data 2 for list of DE genes in each parent-child pair and across all 4 pairs). **b**, Dot plot results for Gene Ontology – Biological Processes (top) and Pathway analysis (bottom), for up-regulated DE genes represented in **a** resulting from the intersection among all 4 parent-child pairs. For each analysis, the top 10 categories in terms of adjusted *p*-value are shown. Dot size represents number of DE genes that fall into each classification category, dot color is the adjusted *p*-value, and the *x*-axis represents the percentage of genes in each category that are DE expressed genes in the RNA-Seq libraries. Notice the presence of up-regulated genes involved in cellular senescence (related to Fig. 5) and tissue architecture. **c**, TPM expression abundances of *HOPX* in parent and PTHS NPCs in 2D culture. Notice the tendency for increased expression in all lines, but lack of statistical significance across groups. N = 4 subjects per group (symbols), 3 independent libraries per subject. **d**, MA plot depicting DE genes between control and PTHS NPCs in 2D culture, to evidence upregulation of *HOPX* in the patient line. Only parent-patient pair #4 is shown as an example (see Supplementary Data 2 for list of DE genes in other pairs). Gray dots are genes that are not statistically significantly differentially expressed between control and PTHS. Colored dots are statistically significant DE genes, and the color indicates adjusted *p*-value. Red dot represents *HOPX*. The *x*-axis represents average normalized expression across all libraries ('baseMean'). **e**, Left: Violin plots showing comparison between parent and PTHS CtOs and GbOs in terms of *HOPX* expression. N = 3544 (parent) or 2944 (PTHS) CtO cells, or 6466 (parent) or 3792 (PTHS) GbO cells. Right: Bar plots show percentages of cells in CtOs and GbOs expressing *HOPX* above threshold corresponding to 40% of the respective mean (shown by the red dashed line in the corresponding violin plots). Notice the diverging regulation patterns of *HOPX* in CtOs and GbOs. Data were obtained from scRNA-Seq results from parent #4 and PTHS #4 CtOs and GbOs that have been maintained for 8 weeks in vitro. The hatched pattern in the bar plots indicates the fraction of *HOPX*+ cells that are also positive for astrocytic marker *S100B* expression. **f**, Left: Fluorescence microscopy images of PTHS and control CtOs and GbOs, after immunostaining for HOPX (yellow). High magnification insets show overlap between HOPX and DAPI staining. (Right): Quantification of

percentages of HOPX+ cells in PTHS and parent organoids. N = 6 sections per group (symbols); 3 sections per subject; 3 ROIs per section. **g**, Relative expression (RT-qPCR) of *HOPX* in the post-mortem PTHS cortex sample (PTHS #6 patient; Supplementary Table 1), in comparison with a control cortex. N = 3 independent replicates per group, 4 technical replicates. **h**, Dot plot results for Gene Ontology – Biological Processes (top) and Pathway analysis (bottom), for down-regulated DE genes shown in **a** resulting from the intersection among all 4 parent-child pairs. For each analysis, the top 10 categories in terms of adjusted *p*-value are shown. Dot size represents number of DE genes that fall into each classification category, dot color is the adjusted *p*-value, and the *x*-axis represents the percentage of genes in each category that are DE expressed genes in the RNA-Seq libraries. Notice the presence of down-regulated genes in the Wnt signaling pathway (related to Fig. 6). Symbols in bar graphs indicate parent-patient identities: diamonds, pair #1; squares, pair #2; triangles, pair #3; circles, pair #4; gray dots, post-mortem samples. Colors in bar graphs and violin plots represent parents (orange), PTHS (blue), or control post-mortem sample (black) groups. Bar graphs represent mean + SEM. ns, not statistically significantly different, $^{**}p<0.01$, $^{***}p<0.001$; two-sample Welch's t-test assuming unequal variances (**c,f,g**) or two-tailed Wilcoxon-Mann-Whitney U test (**e**). Scale bar is 100 μm. See Supplementary Data 1 for statistical test results, including sample sizes, numbers of replicates, exact *p*-values, and effect sizes.

Supplementary Figure 8

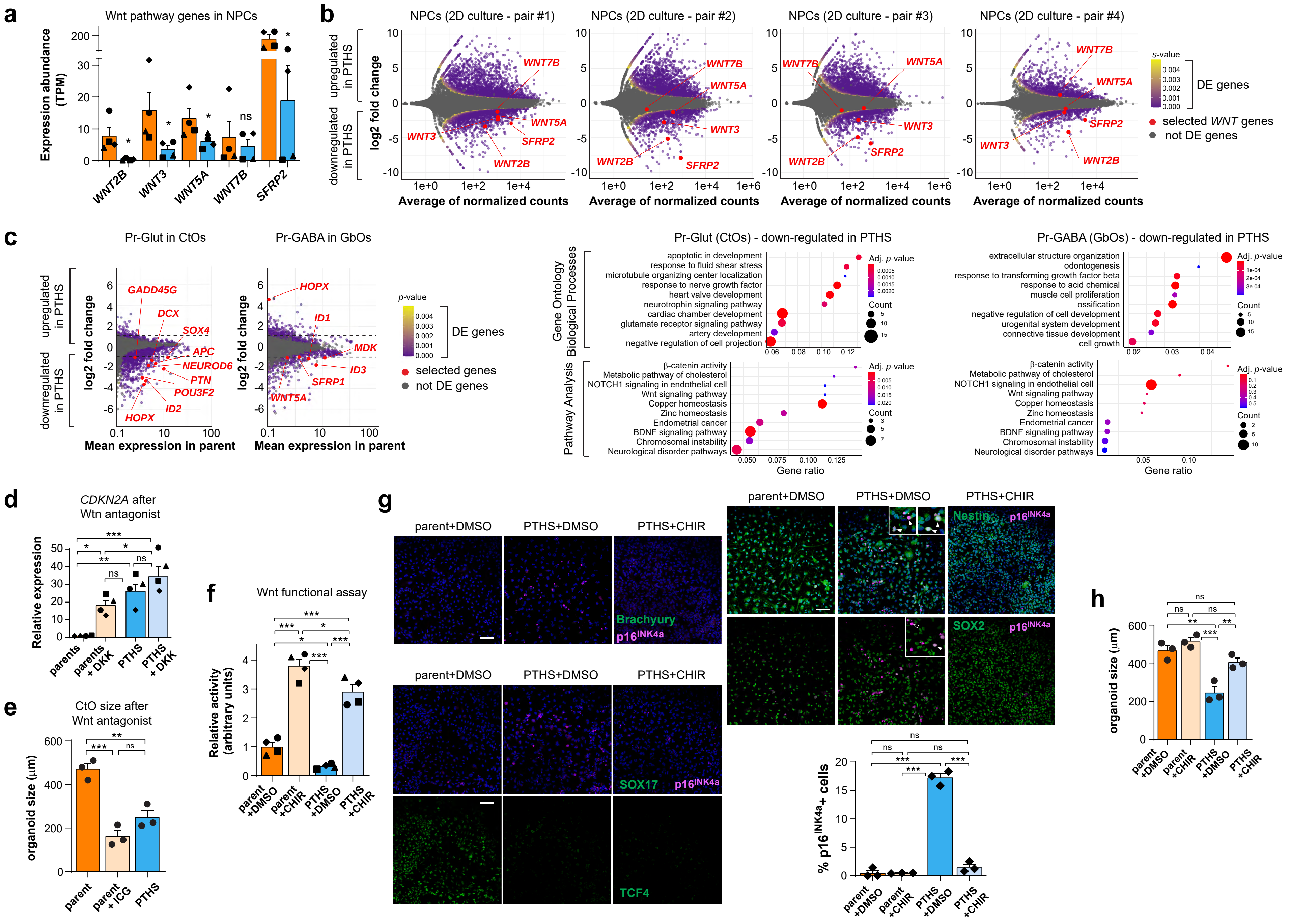

Supplementary Fig. 8: Additional controls for Wnt signaling investigation in organoids and NPCs in 2D culture.

a, Expression abundances for selected genes in the Wnt signaling pathway, comparing parent (orange) and PTHS (blue) NPCs in 2D culture. N = 4 subjects per group (symbols), 3 independent replicates per subject. **b**, MA plot showing genes expressed in control and PTHS NPCs in 2D culture. Each parent-patient comparison is shown separately. Gray dots are genes that are not statistically significantly differentially expressed between control and PTHS. Colored dots are statistically significant DE genes, and the color indicates adjusted *p*-value. The *x*-axes represent average normalized expression across all libraries ('baseMean'). Red dots represent the Wnt signaling pathway genes depicted in **a**. See also Supplementary Data 2 for other DE genes. N = 3 independent RNA-Seq libraries per subject. **c**, DE genes in Pr-Glut and Pr-GABA progenitors of CtOs and GbOs, comparing parent and PTHS organoids, computed from scRNA-Seq data from parent-patient pair #4. Left: MA plot showing DE genes. Each dot represents a gene, gray color indicates genes that are not DE between parent and PTHS groups, and the yellow-to-purple gradient represents DE genes, color-coded according to *s*-value. The *x*-axis represents average gene expression in the parent group. log2FC in *y*-axis indicates the fold-change (in $\log_2$ scale). Genes with average expression below 0.1 in the parent group are not shown, since their expression is small, and most are not DE. Selected DE genes described in the study are highlighted in red. Right: Dot plots representing results of Gene Set Enrichment Analysis followed by GO analysis (Biological Processes and Pathways) over DE genes that are down-regulated in PTHS Pr-Glut progenitors in CtOs (left) or down-regulated in PTHS Pr-GABA progenitors in GbOs (right). For each analysis, the top 10 categories in terms of adjusted *p*-value are shown. Dot size represents number of DE genes that fall into each classification category, dot color is the adjusted *p*-value, and the *x*-axis represents the percentage of genes in each category that are DE expressed genes in the scRNA-Seq libraries. Data were computed from scRNA-Seq data from parent-patient pair #4. **d**, Treatment of parent-derived NPCs in 2D culture with Wnt signaling antagonist DKK-1 increases expression of senescence marker *CDKN2A*. N = 4 parent-patient pairs (symbols), 4 biological replicates per subject, 2 technical replicates. **e**, Treatment of control CtOs with Wnt pathway antagonist ICG-001 (yellow bar) phenocopies small organoid size of PTHS organoids (blue bar). N = 3 independent replicates (circles), 12-30 measured organoids per experiment in each group. Organoids were derived from parent-patient pair #4. **f**, Confirmation of Wnt signaling activity increase after treatment of NPCs in 2D culture with Wnt agonist CHIR99021, as measured by TOP-Flash functional reporter assay. N = 4 subjects per group (symbols), 3 independent replicates per subject. Mean

activity (arbitrary units) was normalized to 1 in ‘parents+DMSO’ group. **g**, Fluorescence microscopy images of NPCs in 2D culture from parent and PTHS subjects treated with CHIR99021 (or DMSO as a control) after staining for mesoderm marker Brachyury and senescence marker $p16^{INK4a}$ (top, left), endoderm marker SOX17, or TCF4 (bottom, left), and neural lineage marker Nestin and SOX2 (right). Filled arrowheads in high magnification insets represent co-localization; open arrowheads indicate absence of co-localization between $p16^{INK4a}$ and SOX2 in some cells (as observed in Supplementary Fig. 6g). The graph at the bottom right shows the quantification of percentages of $p16^{INK4a}$+ cells in NPCs of parent-patient pair #1 in 2D culture treated with CHIR99021. N = 3 independent biological replicates per group. See also Fig. 6j for similar results with parent-pair #4 cells. **h**, Treatment of CtOs with Wnt pathway agonist CHIR99021 at the beginning of the progenitor proliferation phase (light blue bar) rescues organoid size measured at 4 weeks in vitro. N = 3 independent replicates (circles), 15-20 measured organoids per experiment in each group. Organoids were from parent-patient pair #4. Symbols in bar graphs indicate parent-patient identities: diamonds, pair #1; squares, pair #2; triangles, pair #3; circles, pair #4. Colors in bar graphs represent parents (orange), pharmacologically treated parents (yellow), PTHS (blue), or pharmacologically treated PTHS (light blue) groups. Bar graphs represent mean + SEM. n.s., not statistically significantly different, **p*<0.05, ***p*<0.01, ****p*<0.001; two-sample Welch’s t-test in **a** and one-way ANOVA followed by HSD post-hoc test elsewhere. In **d**, mean gene expression was normalized to 1 in the parents group. In **f**, Wnt signaling activity in parent + DMSO group was set to 1. Scale bars are 100 μm. See Supplementary Data 1 for statistical test results, including sample sizes, numbers of replicates, exact *p*-values, and effect sizes.

Supplementary Figure 9

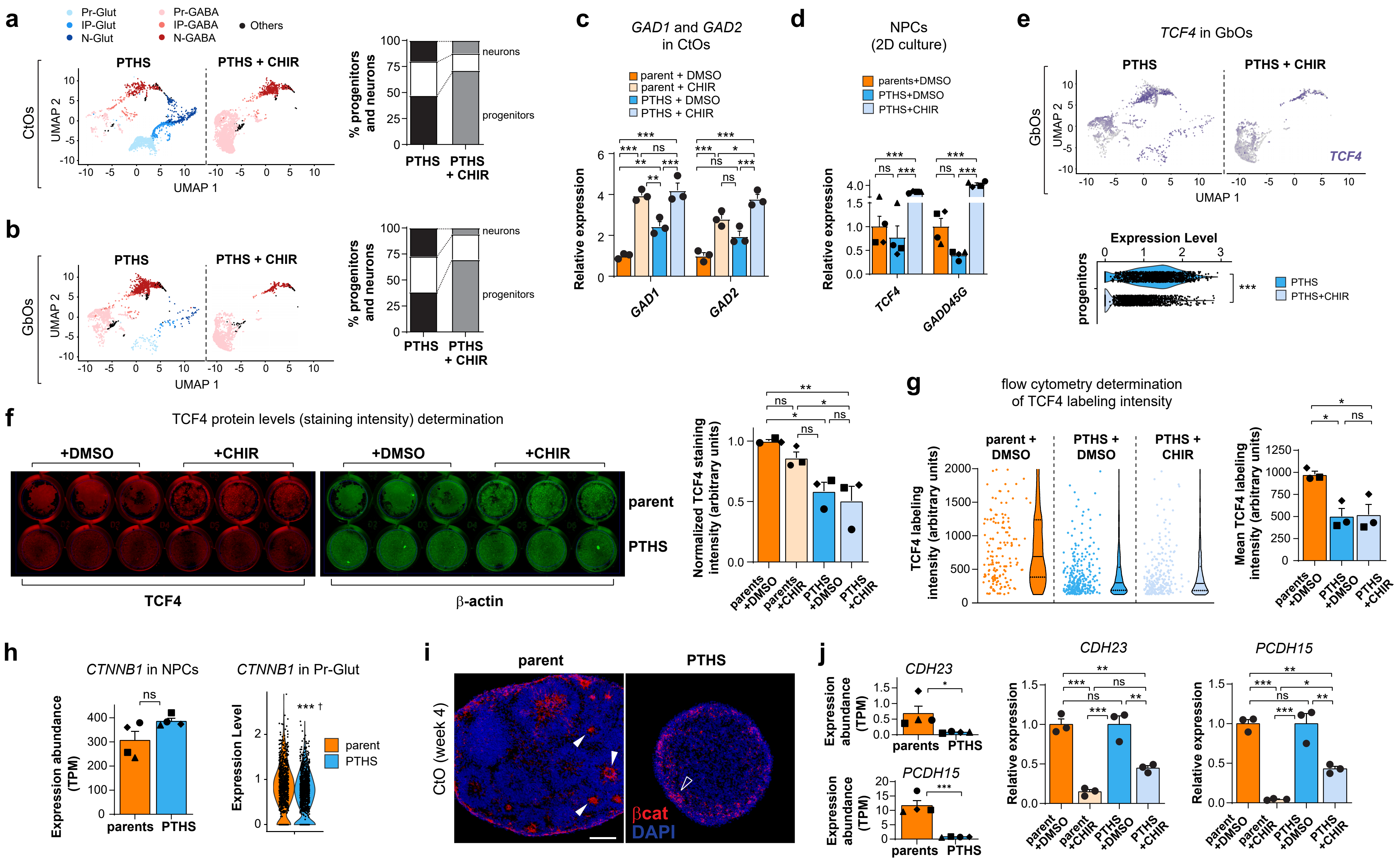

Supplementary Fig. 9: Additional controls for Wnt signaling investigation in organoids and NPCs in 2D culture (part 2).

a,b, Treatment of CtOs (**a**) and GbOs (**b**) at 8 weeks in vitro with Wnt agonist CHIR99021 increases progenitor population, as revealed by single cell RNA-Seq data from parent-patient pair #4. Left: Comparison of UMAP embeddings of cellular diversity in PTHS and CHIR99021-treated PTHS CtOs and GbOs, showing 6 subpopulations of cells, according to color code in Fig. 3a. Cells in 'Others' category are shown in black. Pr-Glut: neural progenitor cells in glutamatergic lineage; IP-Glut: intermediate progenitors in glutamatergic lineage; N-Glut: glutamatergic neurons; Pr-GABA: neural progenitors in inhibitory lineage; IP-GABA: intermediate progenitors in inhibitory lineage; N-GABA: neuronal population containing GABAergic interneurons. Right: quantification of percentages of neural progenitors and neurons in the corresponding types of organoids on the left. **c**, Relative expression of *GAD1* and *GAD2* in CtO organoids at 8 weeks in vitro after treatment with CHIR99021 at the beginning of the progenitor proliferation phase. N = 3 biological replicates per condition for parent/child pair #4, 3 technical replicates. **d**, Treatment of PTHS NPCs in 2D culture with CHIR99021 increases expression of *TCF4* and TCF4 downstream target gene *GADD45G*. N = 4 subjects per group (symbols), 3 independent replicates per subject, 2 technical replicates. Mean expression level was normalized to 1 in 'parents+DMSO' group for each gene. **e**, Top: UMAP showing expression of *TCF4* in PTHS GbOs treated with CHIR99021 (right) in comparison with untreated PTHS GbOs (left). Data was computed from scRNA-Seq results obtained with parent-patient pair #4 organoids at 8 weeks in vitro. Intensity of purple indicates relative expression level. Bottom: Violin plot showing expression level of *TCF4* in single cells (dots) of untreated and CHIR-treated PTHS GbOs. N = 6,466 (PTHS) or 3,792 (PTHS+CHIR) cells. **f**, Left: Representative images of parental control and CHIR99021-treated NPCs in 2D culture seeded onto well plates, after immunostaining for TCF4 and β-actin (as a normalization control). Cells in representative images are from parent-patient pair #1. Right: Quantification of normalized TCF4 expression levels, as judged by fluorometric analysis of staining on the left (see Methods for details). N = 3 subjects per group (symbols), 3 replicates (wells) per subject. **g**, Left: Representative plots showing the levels of TCF4 labeling fluorescence intensity via flow cytometry in PTHS NPCs in 2D culture subjected to treatment with CHIR99021 (see Methods for details), which are indicative of TCF4 protein expression levels. Each dot represents a cytometer event. The violin plots summarize the distribution of TCF4 fluorescence intensity for each group (medians are shown as thick horizontal lines and upper and lower quartiles are

shown as dotted lines). NPCs in the representative examples are from parent-patient pair #1. N = 178 (parent+DMSO), 833 (PTHS+DMSO), or 948 (PTHS+CHIR) cytometer events. Right: Quantification of the mean TCF4 protein levels across parent and PTHS NPC lines (2D culture), as judged by flow cytometry. N = 3 parent-patient pairs (symbols); 3 replicates per subject. **h**, Expression levels for *CTNNB1* (coding for β-catenin) in NPCs in 2D culture (left) or progenitors of the excitatory lineage in CtOs at 8 weeks in vitro (right). For RT-qPCR results on the left, N = 4 subjects per group (symbols), 3 biological replicates per subject. For single cell transcriptomic results on the right, data are from parent #4 and PTHS #4 CtOs. **i**, Fluorescence microscopy images of CtOs at 4 weeks in vitro after staining for β-catenin, showing localization at the centers of rosettes in control organoids, but disorganized staining in PTHS CtOs (arrowheads). **j**, Left: TPM expression abundances of genes coding for cadherin 23 (*CDH23*) and protocadherin 15 (*PCDH15*) in NPCs in 2D culture, which are DE genes between parent and PTHS NPCs in RNA-Seq libraries (see Supplementary Data 2). N = 4 subjects per group (symbols), 3 biological replicates per subject. Right: Graphs represent expression levels of *CDH23* and *PCDH15* in NPCs in 2D culture treated with Wnt agonist CHIR99021. N = 3 biological replicates (circles), 3 technical replicates for cells from parent-patient pair #4. Symbols in bar graphs indicate parent-patient identities: diamonds, pair #1; squares, pair #2; triangles, pair #3; circles, pair #4. Colors in bar graphs and violin plots represent parents (orange), pharmacologically treated parents (yellow), PTHS (blue), or pharmacologically treated PTHS (light blue) groups. Bar graphs represent mean + SEM. n.s., not statistically significantly different, *$p<0.05$, **$p<0.01$, ***$p<0.001$; two-sample Welch's t-test in **h** and **j** (left graphs), two-tailed Wilcoxon-Mann-Whitney U test (right graph in **h**), and one-way ANOVA followed by HSD post-hoc test elsewhere. In **c,d,j**, mean expression in parents + DMSO groups was set to 1 for each gene. The cross symbol in **h** indicates that the means of gene expression on a per cell basis was statistically significantly different between the parent and PTHS groups, but the log2 fold change between PTHS and parents was smaller than an arbitrary value of 0.5 (equivalent to a variation of approximately 40% compared to the parent group mean). Scale bar is 100 μm. See Supplementary Data 1 for statistical test results, including sample sizes, numbers of replicates, exact *p*-values, and effect sizes.

Supplementary Figure 10

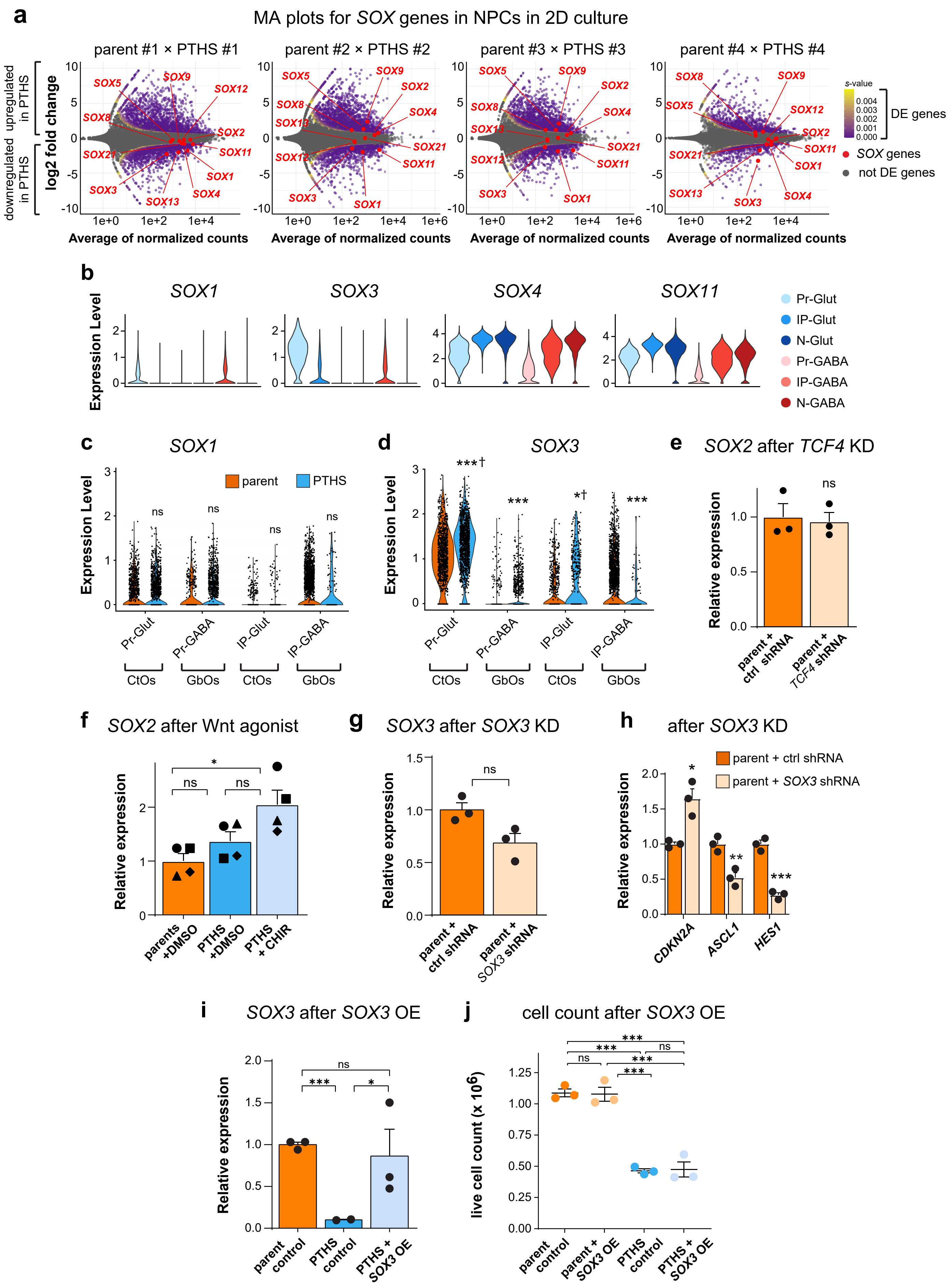

Supplementary Fig. 10: Control data for investigation of *SOX* genes in organoids and NPCs in 2D culture.

a, MA plot showing genes expressed in control and PTHS NPCs in 2D culture. Each parent-patient comparison is shown separately. Gray dots are genes that are not statistically significantly differentially expressed between control and PTHS. Colored dots are statistically significant DE genes, and the color indicates adjusted *p*-value. The *x*-axes represent average normalized expression across all libraries ('baseMean'). Red dots represent *SOX* genes. See also Supplementary Data 2 for other DE genes. N = 3 independent RNA-Seq libraries per subject. **b**, Violin plots showing expression of *SOX1*, *SOX3*, *SOX4*, and *SOX11* in cellular subpopulations in single cell transcriptomic data from CtOs and GbOs at 8 weeks in vitro. See Supplementary Fig. 3b for *SOX2* expression. Pr-Glut: neural progenitor cells in glutamatergic lineage; IP-Glut: intermediate progenitors in glutamatergic lineage; N-Glut: glutamatergic neurons; Pr-GABA: neural progenitors in inhibitory lineage; IP-GABA: intermediate progenitors in inhibitory lineage; N-GABA: neuronal population containing GABAergic interneurons. Data were computed from scRNA-Seq results for parent-patient pair #4. **c,d**, Expression of *SOX1* (**c**) and *SOX3* (**d**) in progenitors (Pr-Glut) and intermediate progenitors (IP-Glut) of CtOs at 8 weeks in vitro, and in progenitors (Pr-GABA) and intermediate progenitors (IP-GABA) of GbOs at 8 weeks in vitro. Each dot represents a single cell; violin plots represent distribution of gene expression in each population; N = 959 and 1230 Pr-Glut cells in parent and PTHS CtO groups, respectively; N = 717 and 382 IP-Glut cells in parent and PTHS CtO groups, respectively; N = 346 and 1376 Pr-GABA cells in parent and PTHS GbO groups, respectively; N = 2737 and 105 IP-GABA cells in parent and PTHS GbO groups, respectively. Data were obtained from parent #4 and PTHS #4 CtOs and GbOs (see also Fig. 3 and Supplementary Figs. 3,4). **e**, *SOX2* expression (RT-qPCR) remains unchanged after *TCF4* knockdown in control NPCs in 2D culture. N = 3 independent experiments (circles) with parent #4 cells, 2 technical replicates per sample. **f**, Treatment of PTHS NPCs in 2D culture with CHIR99021 does not significantly change *SOX2* expression (RT-qPCR). N = 4 subjects per group (symbols), 3 independent experiments, 2 technical replicates per sample. **g**, Relative expression of *SOX3* (RT-qPCR) after shRNA-mediated *SOX3* knockdown in control NPCs in 2D culture. N = 3 independent replicates (circles), 2 technical replicates per sample. There is a tendency for lower expression, which was short of significant ($P \approx 0.50$) but in the expected direction. Mean expression in the parent + control shRNA group was normalized to 1. NPCs used were from parent #4 line. **h**, Relative expression (RT-qPCR) of senescence marker *CDKN2A* and pro-neural genes *ASCL1 and HES1* after shRNA-mediated *SOX3* knockdown in control NPCs in 2D culture. N = 3

independent replicates (circles), 2 technical replicates per sample. NPCs used were from parent #4 line. **i**, Relative expression of *SOX3* after *SOX3* over-expression in NPCs in 2D culture. N = 3 biological replicates (circles), 3 technical replicates, with cells from parent-patient pair #4. **j**, Live cell count in parent and PTHS NPCs subjected to *SOX3* over-expression. N = 3 biological replicates (circles), with cells from parent-patient pair #4. Symbols in bar graphs indicate parent-patient identities: diamonds, pair #1; squares, pair #2; triangles, pair #3; circles, pair #4. Colors in bar graphs and dot and violin plots (except in **b**) represent parents (orange), genetically manipulated parents (yellow), PTHS (blue), or genetically manipulated PTHS (light blue) groups. Bar graphs represent mean + SEM. n.s., not statistically significantly different, *$p<0.05$, **$p<0.01$, ***$p<0.001$; two-sample Welch's t test assuming unequal variances (**e,g,h**), Kruskal-Wallis H test to compare gene expression in PTHS versus respective parent (**c,d**), and one-way ANOVA followed by Tukey-Kramer HSD *post-hoc* test elsewhere. In **g**, comparisons between parent and respective PTHS groups yielded statistical significance at the limit *p*-value of 0.5. The cross symbol indicates that the means of gene expression on a per cell basis was statistically significantly different between the parent and PTHS groups, but the log2 fold change between PTHS and parents was smaller than an arbitrary value of 0.5 (equivalent to a variation of approximately 40% compared to the parent group mean). See Supplementary Data 1 for statistical test results, including sample sizes, numbers of replicates, exact *p*-values, and effect sizes.

Supplementary Figure 11

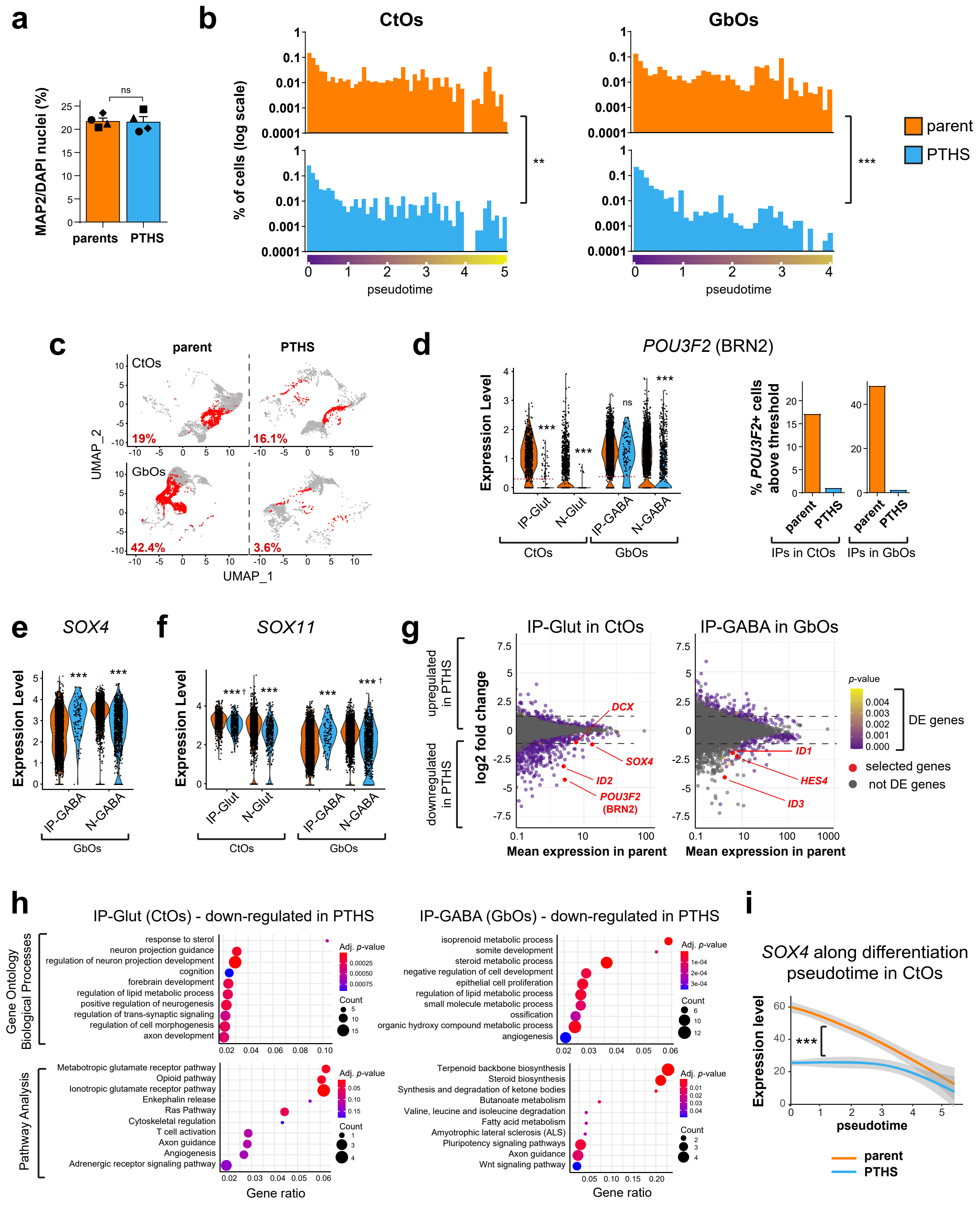

Supplementary Fig. 11: Controls showing impairment of neuronal differentiation in PTHS.

a, Quantification of percentages of MAP2+ cells in 2D cultures of differentiating neurons derived from parental controls and PTHS subjects (2 months in neuronal medium). N = 4 subjects per group (symbols), 2 independent differentiation experiments, 3 independent replicates per subject, 4 counted randomly chosen fields of view per independent replicate. **b**, Histograms representing the fractions of cells at each stage along the differentiation trajectory pseudotime in parent and PTHS CtOs and GbOs, showing the skewed distribution of cells towards early timepoints in PTHS organoids. Binwidth is 0.1 in arbitrary pseudotime scale. Rainbow colormaps below the graphs represent the differentiation pseudotime, as shown in Fig. 3b. See Methods for details on pseudotime and differentiation trajectory analysis. **c**, UMAP embedding of single cell RNA-Seq results in PTHS and parental control CtOs (top) and GbOs (bottom) at 8 weeks in vitro, highlighting the intermediate progenitors (IP) in red in each plot. The percentages of IPs are displayed in red at the bottom left of each quadrant. Data were derived from scRNA-Seq results for parent-patient pair #4. **d**, Left: Violin plots showing expression of *POU3F2* (which encodes BRN2, expressed in intermediate progenitors) in IPs and neurons of CtOs and GbOs at 8 weeks in vitro. Each dot represents a single cell. Right: Severe reduction in the percentage of intermediate progenitors in PTHS CtOs and GbOs, as judged from quantification of *POU3F2*+ cellular populations in single cell RNA-Seq data from parent-patient pair #4. Color code for violin plots is the same as in **a**. N = 717 and 382 IP-Glut cells in parent and PTHS CtO groups, respectively; N = 1401 and 380 N-Glut neurons in parent and PTHS CtO groups, respectively; N = 2737 and 105 IP-GABA cells in parent and PTHS GbO groups, respectively; N = 2661 and 988 N-GABA neurons in parent and PTHS GbO groups, respectively. **e**, *SOX4* expression in intermediate progenitors (IP-GABA) and neurons (N-GABA) in parent (orange) and PTHS (blue) GbOs at 8 weeks in vitro. Each dot represents a single cell; violin plots represent distribution of gene expression in each population; N = 2737 and 105 IP-GABA cells in parent and PTHS groups, respectively; N = 2661 and 988 N-GABA neurons in parent and PTHS groups, respectively. Organoids are from parent-patient pair #4. Although expression was not changed per cell, there is a clear reduction in the number of both intermediate progenitors and neurons in the PTHS organoids. **f**, *SOX11* expression in intermediate progenitors (IP-Glut and IP-GABA) and neurons (N-Glut and N-GABA) in parent (orange) and PTHS (blue) CtOs and GbOs at 8 weeks in vitro. Each dot represents a single cell; violin plots represent distribution of gene expression in each population; N = 717 and 382 IP-Glut cells in parent and PTHS CtO groups, respectively; N = 1401 and 380 N-Glut neurons in parent and PTHS CtO groups, respectively; N = 2737 and 105 IP-GABA cells in parent and PTHS GbO groups, respectively; N = 2661 and

988 N-GABA neurons in parent and PTHS GbO groups, respectively. Organoids are from parent-patient pair #4. **g**, Left, MA plot showing DE genes in IP-Glut and IP-GABA intermediate progenitors of CtOs and GbOs, comparing parent and PTHS organoids, computed from scRNA-Seq data from parent-patient pair #4. Each dot represents a gene, gray color indicates genes that are not DE between parent and PTHS groups, and the yellow-to-purple gradient represents DE genes, color-coded according to *s*-value. The *x*-axis represents average gene expression in the parent group. log2FC in the *y*-axis indicates fold-change (in $\log_2$ scale). Genes with average expression below 0.1 in the parent group are not shown, since their expression is small, and most are not DE. Selected DE genes described in the study are highlighted in red. **h**, Dot plots representing results of Gene Set Enrichment Analysis followed by GO analysis (Biological Processes and Pathways) over DE genes that are down-regulated in PTHS IP-Glut intermediate progenitors in CtOs (left) or down-regulated in PTHS IP-GABA intermediate progenitors in GbOs (right). For each analysis, the top 10 categories in terms of adjusted *p*-value are shown. Dot size represents number of DE genes that fall into each classification category, dot color is the adjusted *p*-value, and the *x*-axis represents the percentage of genes in each category that are DE expressed genes in the scRNA-Seq libraries. Data were computed from scRNA-Seq data from parent-patient pair #4. **i**, Expression of SOX4 along the differentiation trajectory path depicted in Fig. 3b, in parent and PTHS CtOs. The shaded areas around each line indicate 95% confidence intervals at each timepoint along the pseudotime scale (see Methods for details). Data were computed from scRNA-Seq data from parent-patient pair #4. Symbols in bar graphs indicate parent-patient identities: diamonds, pair #1; squares, pair #2; triangles, pair #3; circles, pair #4. Colors in bar and line graphs and violin plots represent parents (orange) or PTHS (blue) groups. Bar graphs represent mean + SEM. n.s., not statistically significantly different, ***$p<0.001$; two-sample Welch's t test assuming unequal variances (**a**), Kruskal-Wallis H test to compare gene expression in PTHS versus respective parent (**d,e,f**), or Kolmogorov-Smirnov two-sample distribution test (**b,i**). The cross symbol indicates that the means of gene expression on a per cell basis was statistically significantly different between the parent and PTHS groups, but the log2 fold change between PTHS and parents was smaller than an arbitrary value of 0.5 (equivalent to a variation of approximately 40% compared to the parent group mean). See Supplementary Data 1 for statistical test results, including sample sizes, numbers of replicates, exact *p*-values, and effect sizes.

Supplementary Figure 12

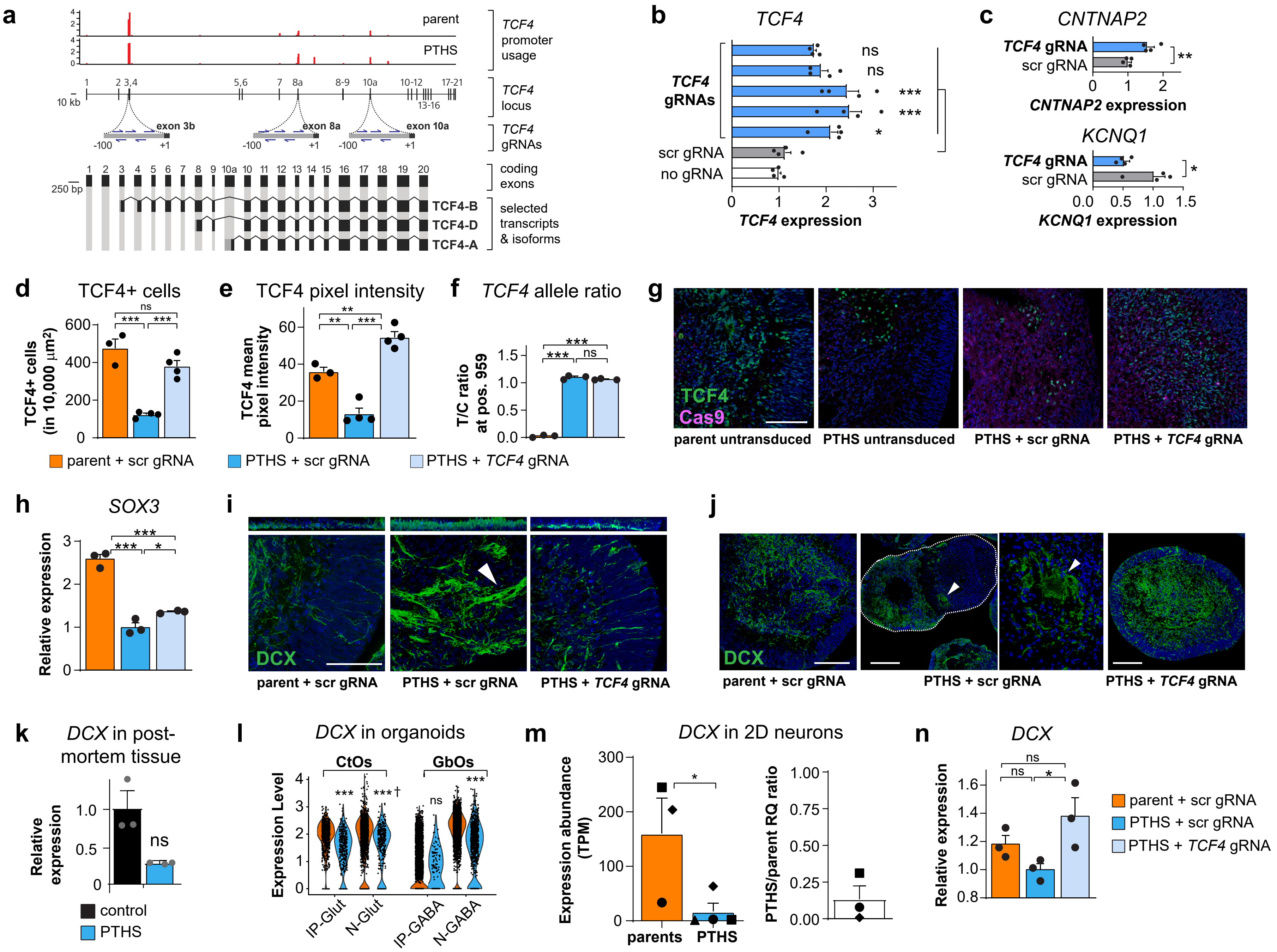

Supplementary Fig. 12: Controls for CRISPR-mediated trans-epigenetic correction of *TCF4* expression.

a, Normalized transcriptional activity from alternative promoters in the *TCF4* locus (red bars), in parent and PTHS samples (first row). The second row depicts a schematic representation of the *TCF4* locus, showing exon locations. The position of the designed gRNAs (blue arrows) is shown for the three chosen *TCF4* alternative promoters, upstream of exons 3b, 8a and 10a (see Methods for details on promoter choice and gRNA design and positioning). The remaining rows show the transcripts formed from transcription initiated at exons 3b, 8a and 10a, which yield TCF4 protein isoforms TCF4-B, TCF4-D, and TCF4-A, respectively[3]. **b**, Testing of transactivation efficiency of five *TCF4* gRNAs on *TCF4* expression in SH-SY5Y cells. scr gRNA, control scrambled gRNA; no gRNA, empty expression construct. N = 4 independent experiments, 3 technical replicates per sample. Statistical results shown are for the comparisons between each *TCF4* gRNA group and the control scr gRNA group. **c**, Trans-epigenetic correction of *TCF4* expression increases *CNTNAP2* expression (top) and decreases *KCNQ1* expression (bottom) in SH-SY5Y cells. N = 4 independent experiments, 3 technical replicates per sample. **d**, Quantification of TCF4+ immunostained cells (in 100 × 100 μm square area) in histological sections from organoids subjected to trans-epigenetic correction of *TCF4* expression. N = 3 (parent + scr gRNA) or 4 (other groups) sections per group, 4 100 × 100 μm ROIs per section. **e**, Quantification of TCF4 staining pixel intensity in histological sections from organoids subjected to trans-epigenetic correction of *TCF4* expression (see Methods for details). N = 3 (parent + scr gRNA) or 4 (other groups) sections per group, 4 100 × 100 μm ROIs per section. **f**, Ratio between expression abundance for the normal (C at position 959 of the coding sequence) and mutated (T at that position) *TCF4* alleles. N = 3 independent replicates per group (circles), 10 pooled organoids per sample, 3 technical replicates. Organoids are from parent-patient pair #4. **g**, Representative images of untransduced and transduced organoids at 2 weeks in vitro (with scr or *TCF4* gRNAs) after immunostaining for dead Cas9 (magenta) and TCF4 (green). Notice the presence of Cas9 immunostaining in transduced organoids, indicating efficient transduction, as well abnormally low TCF4 green fluorescence in PTHS organoids prior to correction of *TCF4* expression (PTHS untransduced and PTHS+scr gRNA), and increased fluorescence after transduction with *TCF4* gRNA. **h**, Expression levels (RT-qPCR) of *SOX3* in organoids at 4 weeks in vitro after trans-epigenetic *TCF4* expression correction. It should be noted that the correction was partial, probably because not all cells in the organoids express *SOX3.* N = 3 independent replicates per group (circles), 10 pooled

organoids per sample, 3 technical replicates. Organoids are from parent-patient pair #4. Bar colors are the same as in **d**. **i**, Large images: fluorescence microscopy images of transduced organoids (at 4 weeks in vitro) in CRISPR-mediate trans-epigenetic *TCF4* correction experiment after immunostaining for doublecortin (DCX; green) to show aberrant clumps of immature neuronal cells in non-corrected PTHS organoids (arrowhead in middle panel). The rectangular images above each panel are z-projections, showing larger dispersion of DCX+ neuronal processes in non-corrected organoids, which is reversed in the PTHS + *TCF4* gRNA group (right). See Methods for details on DCX imaging as *z*-series stacks. **j**, Low magnification fluorescence microscopy images of transduced organoids at 4 weeks in vitro in CRISPR-mediate trans-epigenetic *TCF4* correction experiment after immunostaining for doublecortin (DCX; green), showing aggregation of DCX+ fibers (arrowheads) in the organoid's outgrowths in the PTHS + scr gRNA panel (high magnification inset is shown on the right for the PTHS + scr gRNA condition). **k**, Relative expression of *DCX* in post-mortem PTHS cortex tissue (PTHS #6 subject). N = 3 biological replicates, 4 technical replicates. **l**, Single cell transcriptomic data show that *DCX* expression is reduced in intermediate progenitors (IP-Glut and IP-GABA) and neurons (N-Glut and N-GABA) of PTHS CtOs and GbOs (blue violin plots) at 8 weeks in vitro, as compared to the respective parent CtOs and GbOs (orange violin plots). N = 717 (parent) and 382 (PTHS) IP-Glut cells, 1401 (parent) and 380 (PTHS) N-Glut neurons, 2737 (parent) and 105 (PTHS) IP-GABA cells, or 2661 (parent) and 988 (PTHS) N-GABA neurons. Data were computed from scRNA-Seq experiments with parent-patient pair #4 organoids. **m**, Left: Expression abundance (TPM) of *DCX* in PTHS neurons in 2D culture (3 months in neuronal medium) in comparison with neurons derived from parental controls, as judged from RNA-Seq data. Right: Ratio of relative quantitation of *DCX* expression (RQ) between PTHS and parent samples. N = 3 (parents) or 4 (PTHS) subjects per group (symbols), 3 independent replicate libraries per subject. **n**, Correction of *DCX* expression levels in organoids at 4 weeks in vitro, after trans-epigenetic *TCF4* expression correction. N = 3 independent replicates per group (circles), 10 pooled organoids per sample, 3 technical replicates. Organoids are from parent-patient pair #4. Symbols in bar graphs indicate parent-patient identities: diamonds, pair #1; squares, pair #2; triangles, pair #3; circles, pair #4; gray dots, post-mortem samples. Black dots in **b** and **c** represent independent replicates of transfected cells. Colors in bar graphs and violin plots represent parents (orange), PTHS (blue), genetically manipulated PTHS (light blue), or control post-mortem sample (black) groups, except in **b** and **c**. In **b** and **c**, the blue bars indicate cells that have been transfected with the *TCF4* gRNAs. Bar graphs represent mean + SEM. n.s., not statistically significantly different, *p<0.05, **p<0.01, ***p<0.001; two-sample Welch's t

test (**c,k,m**), Kruskal-Wallis H test to compare gene expression in PTHS versus respective parent (**l**), or one-way ANOVA followed by Tukey-Kramer's HSD post-hoc test elsewhere in the figure. The cross symbol indicates that the means of gene expression on a per cell basis was statistically significantly different between the parent and PTHS groups, but the log2 fold change between PTHS and parents was smaller than an arbitrary value of 0.5 (equivalent to a variation of approximately 40% compared to the parent group mean). Scale bars are 100 μm. See Supplementary Data 1 for statistical test results, including sample sizes, numbers of replicates, exact *p*-values, and effect sizes.

Supplementary Figure 13

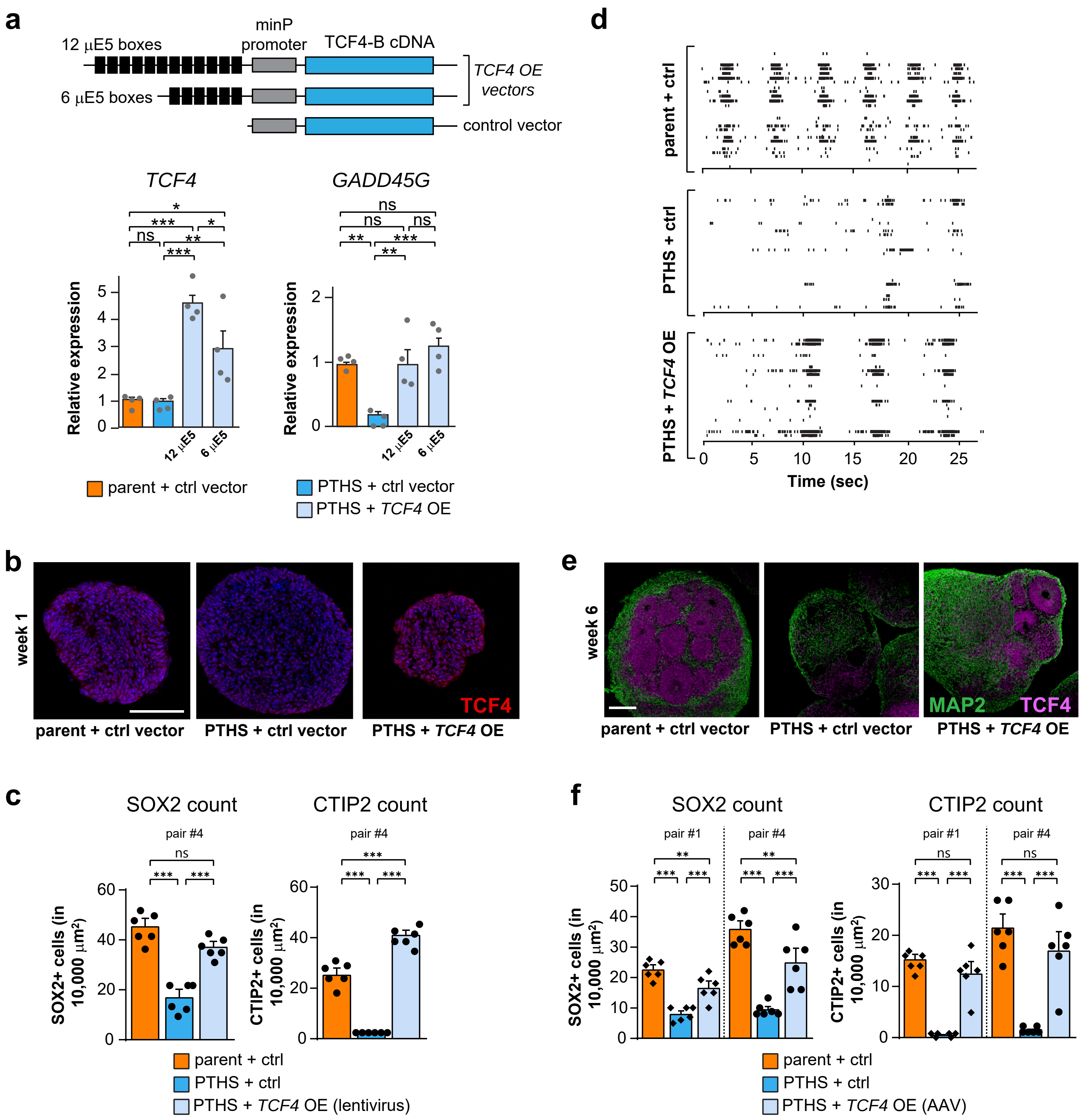

Supplementary Fig. 13: Controls for *TCF4* over-expression experiments in organoids.

a, Validation of correction of *TCF4* expression with over-expression (OE) cassettes (top) containing the cDNA coding for TCF4-B isoform preceded by a synthetic promoter containing micro-E5 (μE5) regulatory binding sites, which allows over-expression in TCF4-expressing cells, thereby preventing ectopic expression. Versions of this construct (with 6 or 12 μE5 boxes) were transfected into PTHS NPCs in 2D culture, followed by RT-qPCR evaluation of expression of *TCF4* and TCF4 target gene *GADD45G*. N = 4 independent replicates per group. Means of relative expression levels are compared for cells transfected with over-expression cassettes containing 6 or 12 μE5 boxes against a control condition transfected with vector containing a minimal promoter (minP), which is not expected to increase *TCF4* levels. Expression of each gene in the parent group is normalized to 1. Cells are from NPCs of parent #4 and PTHS #4. **b**, Fluorescence microscopy images of CtOs at 1 week in vitro, after transduction with OE lentiviral vector containing 12 μE5 boxes (*TCF4* OE) followed by immunostaining for TCF4 (red). **c**, Densities of SOX2+ and CTIP2+ cells in CtOs subjected to *TCF4* OE (evaluated at 6 weeks in vitro). N = 6 biological replicates per subject, for organoids from parent-patient pair #4. **d**, Raster plots showing differences in firing activity between parent (left) and PTHS (top right) CtOs in MEA assay, but partial rescue of activity in PTHS organoids treated with *TCF4* OE (bottom right) lentiviral vectors. Representative plots from 3 independent replicates per group, with 10 seeded organoids per replicate in each group. Each line in the raster plot represents an electrode. **e**, Fluorescence microscopy images of CtOs at 6 weeks in vitro, after transduction with *TCF4* OE AAV vectors, immunostained for MAP2 (green) and TCF4 (magenta), showing rescue of rosettes in PTHS line subjected to *TCF4* OE. **f**, Densities of SOX2 and CTIP2-positive cells in CtOs subjected to *TCF4* OE (evaluated at 6 weeks in vitro). N = 6 biological replicates per subject, for organoids from parent/child pairs #1 or #4. Symbols in bar graphs indicate parent-patient identities: diamonds, pair #1; circles, pair #4. Gray dots in **a** represent independent replicates of transfected cells. Colors in bar graphs represent parents (orange), PTHS (blue), or genetically manipulated PTHS (light blue) groups. Bar graphs represent mean + SEM. n.s., not statistically significant; *p<0.05, **p<0.01, ***p<0.001; one-way ANOVA followed by Tukey-Kramer's HSD post-hoc test in all comparisons. Scale bars are 100 μm. See Supplementary Data 1 for statistical test results, including sample sizes, numbers of replicates, exact p-values, and effect sizes.

Supplementary Figure 14

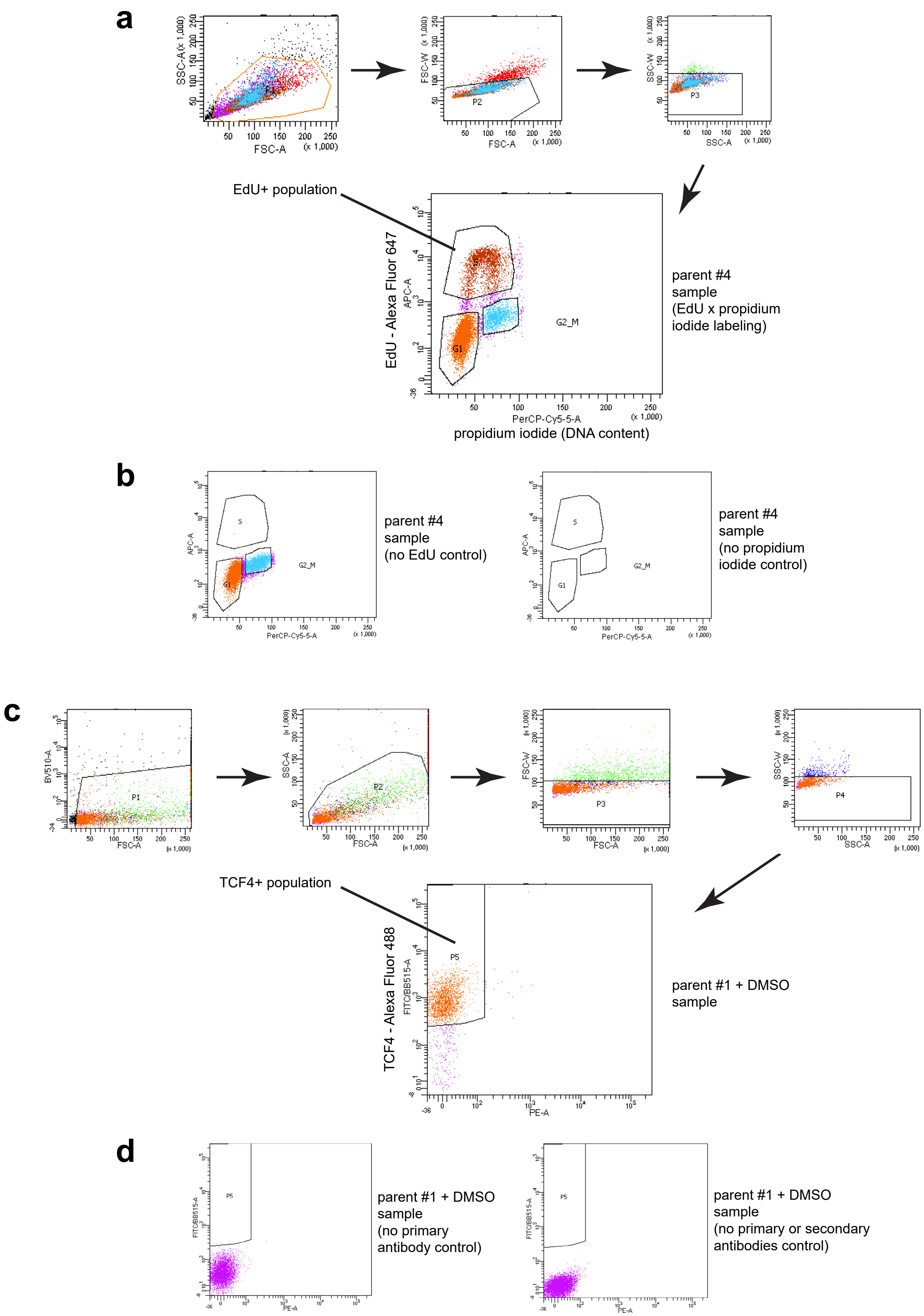

Supplementary Fig. 14: Flow cytometry controls.

a, Example of gating strategy applied to flow cytometry determination of EdU+ cells in NPCs in 2D culture (Fig. 5f). The same gates were applied to all samples. **b**, ‘no EdU’ and ‘no propidium iodide’ negative controls, illustrating the robustness of the gating strategy (see Methods for details). **c**, Example of gating strategy applied to flow cytometry determination of TCF4 expression levels in NPCs in 2D culture after treatment with Wnt agonist CHIR99021 (Supplementary Fig. 9g). The same gates were applied to all samples. **d**, ‘no anti-TCF4 primary antibody’ and ‘no primary or secondary antibodies’ negative controls, illustrating the robustness of the gating strategy (see Methods for details).

Supplementary Table 1: Summary of participating subjects and clinical characteristics.

patient	symbol in figures	type of *TCF4* mutation	PTHS clinical features	abMRI
PTHS #1	◆	small insertion (c.1067_1068insTC)	FG MM CT SZ BA VA	yes
PTHS #2	■	whole gene deletion (del 6.6 q21.2-q21.33)	FG MM AS CT VA RB	yes
PTHS #3	▲	translocation [t(2;18)(chr2:197,989,212-197,989,746_chr18:52,895,297-52,895,597)]	FG SM AS CT VA RB	no
PTHS #4	●	point mutation (c.959C>T ; Thr320Ile)	FG SM AS CT VA	no
PTHS #5	✚	partial gene deletion (c.454_1072del)	FG SM AS SZ RB	yes
PTHS #6	Post-mortem sample	whole gene deletion (q21.5-q21.32.3)	FG MM AS CT VA RB	yes
parent #1	◆	-	-	-
parent #2	■	-	-	-
parent #3	▲	-	-	-
parent #4	●	-	-	-
parent #5	✚	-	-	-

For all patients, controls are parents of matching sex. Abbreviations: FG: dysmorphic facial *gestalt*; SM/MM: severe (SM) or mild (MM) motor delay at age 3; AS: absent speech; CT: constipation; SZ: seizures; BA: breathing problems (hyperventilation or apnea); UA: urinary abnormalities (retention or incontinence); VA: visual abnormalities (ocular anomalies); RB: repetitive behaviors; abMRI: brain anomalies (thinned *corpus callosum*) detected by MRI.

Supplementary Table 2: Percentages of cell types in scRNA-Seq libraries

	CtOs				GbOs		CtOs + CHIR		GbOs + CHIR	
	parent #4 rep. #1	parent #4 rep. #2	parent #4 rep. #3	PTHS #4	parent #4	PTHS #4	PTHS #4	PTHS #4 + CHIR	PTHS #4	PTHS #4 + CHIR
Pr-Glut	27.9%	27.1%	28.7%	41.8%	0.9%	2.1%	41.8%	0.0%	2.1%	0.0%
IP-Glut	18.9%	20.2%	14.9%	13.0%	0.1%	0.8%	13.0%	0.0%	0.8%	0.0%
N-Glut	41.1%	39.5%	29.7%	12.9%	0.0%	1.1%	12.9%	0.0%	1.1%	0.0%
Pr-GABA	2.9%	2.4%	9.1%	5.3%	5.4%	36.3%	5.3%	71.2%	36.3%	69.5%
IP-GABA	0.1%	0.1%	0.7%	3.1%	42.3%	2.8%	3.1%	1.5%	2.8%	0.5%
N-GABA	0.7%	0.5%	3.2%	7.1%	41.2%	26.1%	7.1%	12.4%	26.1%	5.9%
Others	2.7%	3.1%	6.0%	4.8%	3.2%	9.8%	4.8%	3.8%	9.8%	5.5%
high mtRNA	5.8%	7.1%	7.7%	12.0%	7.0%	21.2%	12.0%	11.1%	21.2%	18.7%
Total	100.0%	100.0%	100.0%	100.0%	100.0%	100.0%	100.0%	100.0%	100.0%	100.0%

Supplementary References

1. Kim, H. *et al.* Pluripotent Stem Cell-Derived Cerebral Organoids Reveal Human Oligodendrogenesis with Dorsal and Ventral Origins. *Stem Cell Reports* **12**, 890–905 (2019).

2. Hernandez-Segura, A. *et al.* Unmasking Transcriptional Heterogeneity in Senescent Cells. *Curr. Biol.* **27**, 2652-2660.e4 (2017).

3. Sepp, M., Kannike, K., Eesmaa, A., Urb, M. & Timmusk, T. Functional Diversity of Human Basic Helix-Loop-Helix Transcription Factor TCF4 Isoforms Generated by Alternative 5′ Exon Usage and Splicing. *PLoS One* **6**, e22138 (2011).
