# Supplementary material for: Transcription Factor 4 loss-of-function is associated with deficits in progenitor proliferation and cortical neuron content
Source: Nat Commun. 2022 May 2;13:2387. doi: 10.1038/s41467-022-29942-w (PMC9061776; doi:10.1038/s41467-022-29942-w)
Supplement: Supplementary file 2 — Description of Additional Supplementary Files [file 41467_2022_29942_MOESM2_ESM.pdf]

Description of Additional Supplementary Files

Supplementary Data 1: Summary of statistical tests and metrics.

For all figure panels (listed in order of appearance in the paper), we present complete statistical evaluation results, including type of analysis, type of cellular model, groups being compared, means and standard errors of the means (SEM) for each group, numbers of subjects, independent replicates, technical replicates (or numbers of cells), as well as the number of observations used to calculate means and perform statistical comparisons (N), the statistical test used, metrics results (*t, H, W,* or *F*), effect sizes, exact *p*-values for global statistical comparisons, and, when necessary, effect sizes and exact *p*-values for each individual pairwise comparison after post-hoc test. When experimentation involved more than one independent replicate per subject cell line, or more than one technical replicate per independent biological replicate, these numbers are also indicated in separate columns, even though the statistical test was run based solely on the means of different lines in these cases. The comparisons of electrophysiological measurements are presented in the second tab (part 2). The comparisons of gene expression between parent and PTHS single cell RNA-Seq libraries are listed in the third tab (part 3), including gene name, cell types, groups being compared, means and SEM, fold change (PTHS versus parent), as well as statistical test results.

Supplementary Data 2: Gene expression datasets for neural progenitor cells and neurons and lists of DE genes between PTHS and parents.

Tab 'neural progenitors': Gene expression abundance values for RNA sequencing libraries from NPCs of subjects PTHS #1 to PTHS #4 and respective parents. Gene expression is quantified in transcripts per million reads (TPM) aggregated to gene level (see Methods for details). Gene names follow nomenclature in GRCh38 (GENCODE 32) human genome build. Independent replicates are denoted as A, B, and C.

Tab 'DE gene neural progenitors': Metrics of differential expression (DE) analysis for pairwise comparisons (each parent versus respective PTHS child) obtained using DESeq2 (see Methods for details). For each comparison, 'baseMean' indicates across-sample average of normalized counts aggregated at the gene level, 'log2FoldChange' is the apeglm-shrunken log2 fold change (effect size), 'lfcSE' is the standard error of log2 fold change, and 's-value' indicates the estimated rate of false sign among genes with equal or smaller *s*-values. The last two columns indicate whether the difference in expression between the parent and respective PTHS child is statistically significant (genes with *s*-values smaller than 0.005) and the direction of change.

Tab 'Common DE – progenitors': Lists and numbers of DE genes in comparisons between 2 and 4 parent-PTHS subject pairs. Cells marked in yellow indicate WNT, cadherin, and protocadherin DE genes in 'Cadherin' and 'Wnt signaling' pathway analysis categories.

Tab 'neurons': Gene expression abundance values for RNA sequencing libraries from neurons of PTHS #1 through #4 patients and parents #1, #2, and #4.

Tab 'DE gene neurons': Metrics of differential expression analysis for pairwise comparisons (parent versus PTHS).

Tab 'Common DE – neurons: List and number of DE genes in comparison between 3 parent-PTHS subject pairs. Genes coding for ion channels are highlighted.

Tab 'neural progenitors-low passage': Gene expression abundance values for RNA sequencing libraries from NPCs of low passage (P5) from subjects PTHS #1 and PTHS #4 and respective parents.

Supplementary Data 3: List of DE genes in organoids.

Each tab contains information on each subpopulation type being compared. The first column in each tab is a list of all genes. The second and third columns indicate the average log-transformed values of expression for each gene across all cells in the parent and PTHS groups (see Methods for details on log-transformation). The fourth and fifth columns indicate the average values of expression (calculated in the non-log space) for each gene across all cells in the parent and PTHS subpopulations being contrasted. 'avg_log2FC' column shows the average log2 fold-change between parent and PTHS across all cells in the subpopulations being contrasted (negative values indicate lower expression in the PTHS group). 'corrected avg_log2FC' is the log2FC corrected by adding 0.0001 to the expression values in each group, to eliminate the issue of dividing by null values in the parent group. The last two columns show the *p*-value (p_value) and adjusted *p*-value (adj p_value) for multiple comparisons, according to the DESEq2 method.

Supplementary Data 4: Assignment of subpopulation identities in scRNA-Seq experiments.

Metadata table informing the barcodes for each individual cell across all 8 single cell RNA-Seq libraries and the corresponding assignment to the 8 types of cellular subpopulations annotated as described in the Methods section. For the excitatory lineage, these are: neural progenitor cells (Pr-Glut), intermediate progenitors (IP-Glut), and glutamatergic neurons (N-Glut). For the inhibitory lineage: neural progenitor cells (Pr-GABA), intermediate progenitors (IP-GABA), and neuronal population containing GABAergic cells (N-GABA). The 'Others' subpopulation consists of a minority group of cells of neural origin which could not be unequivocally assigned to the previous six subpopulations. They were not the focus of our study and were not further analyzed. The 'high_mt' subpopulation includes few cells (usually less than 5% per library) that display high expression of mitochondrial genes (more than 20% median expression) and were therefore excluded from all analyses.

Supplementary Data 5: Sequences of mutated *TCF4* transcripts and *TCF4* expression quantification at the transcript level.

Tab '*TCF4* mut transcript sequences': Reconstructed sequences of all *TCF4* transcripts encompassing the mutation sites found in patients PTHS #1 and #4. Sequences of mutated transcripts for PTHS #2 and #3 are not shown because these patients carry large deletion or translocation mutations.

Tabs '*TCF4* expression in neurons' and '*TCF4* expression in NPCs': Quantification of expression abundances (TPM) at the transcript level for normal and mutated alleles across all patients. As expected, *TCF4* expression levels in patients PTHS #1 to #3 are diminished relative to the respective parents, and each mutated allele is only found in the respective patient.

Supplementary Data 6: List of materials and reagents used in the study.

This table contains oligo and plasmid information, as well as a list of the most important reagents used in the study, to ensure reproducibility of our results.
