# Supplementary material for: Transcription Factor 4 loss-of-function is associated with deficits in progenitor proliferation and cortical neuron content
Source: Nat Commun. 2022 May 2;13:2387. doi: 10.1038/s41467-022-29942-w (PMC9061776; doi:10.1038/s41467-022-29942-w)
Supplement: Supplementary file 9 — Reporting Summary [file 41467_2022_29942_MOESM9_ESM.pdf]

nature research

Corresponding author(s): Fabio Papes & Alyssson R Muotri

Last updated by author(s): Mar 7, 2022

Reporting Summary

Nature Research wishes to improve the reproducibility of the work that we publish. This form provides structure for consistency and transparency in reporting. For further information on Nature Research policies, see our Editorial Policies and the Editorial Policy Checklist.

Statistics

For all statistical analyses, confirm that the following items are present in the figure legend, table legend, main text, or Methods section.

n/a	Confirmed	
[ ]	[x]	The exact sample size (*n*) for each experimental group/condition, given as a discrete number and unit of measurement
[ ]	[x]	A statement on whether measurements were taken from distinct samples or whether the same sample was measured repeatedly
[ ]	[x]	The statistical test(s) used AND whether they are one- or two-sided<br>*Only common tests should be described solely by name; describe more complex techniques in the Methods section.*
[x]	[ ]	A description of all covariates tested
[ ]	[x]	A description of any assumptions or corrections, such as tests of normality and adjustment for multiple comparisons
[ ]	[x]	A full description of the statistical parameters including central tendency (e.g. means) or other basic estimates (e.g. regression coefficient) AND variation (e.g. standard deviation) or associated estimates of uncertainty (e.g. confidence intervals)
[ ]	[x]	For null hypothesis testing, the test statistic (e.g. *F*, *t*, *r*) with confidence intervals, effect sizes, degrees of freedom and *P* value noted<br>*Give P values as exact values whenever suitable.*
[x]	[ ]	For Bayesian analysis, information on the choice of priors and Markov chain Monte Carlo settings
[x]	[ ]	For hierarchical and complex designs, identification of the appropriate level for tests and full reporting of outcomes
[ ]	[x]	Estimates of effect sizes (e.g. Cohen's *d*, Pearson's *r*), indicating how they were calculated

Our web collection on statistics for biologists contains articles on many of the points above.

Software and code

Policy information about availability of computer code

Data collection	Image J (FIJI; version 1.53o); ZEN Lite software (Zeiss; version 3.1); AxIS Software (Axion Biosystems; version 1.0); and CFX Connect Real Time PCR detection system and Maestro software (Bio-Rad; version 1.1).
Data analysis	Bulk RNA-Seq expression abundance estimation: Salmon (version 0.14.1); Pairwise differential expression (DE) comparisons for bulk RNA-Seq data: DESeq2 (version 1.22.1), tximport (version 1.10.1), and apeglm algorithm (version 1.16.0); Gene Ontology (GO) and pathway analyses: online WebGestalt tool (version 2019); Deconvolution of bulk RNA-Seq data: CibersortX (version 2021); RT-qPCR: CFX Connect Real Time PCR detection system and Maestro software (Bio-Rad; version 1.1); MEA data analysis: Neural Metrics Tool (Axion Biosystems; version 2.5.1); Neuronal morphometric measurements: Neurolucida Neuron Tracing Software (MBF Bioscience; version 2017.01.1); Electrophysiology analyses: Axon pCLAMP 10 software (Molecular Devices; version 10.7); Statistical analyses: Prism software (GraphPad; version 9.2.0), RStudio (version 1.4.1106), G*Power (version 3.1), and WebPower (version 2018); Single cell RNA-Seq analyses: Cell Ranger (version 4.0.0), Cell Loupe browser (10x Genomics; version 4.1.0), Seurat (version 3.2.2), DESeq2 (version 3.14), UMAP algorithm (0.1.10), and Monocle 3 (version 0.2.2); Promoter usage analysis: STAR aligner software (version STAR 2.7.6a) and proActiv R package (version 0.99.0); Image processing and cell counting: Image J (FIJI; version 1.53o); EdU assay and TCF4 flow-cytometry quantification: FlowJo (BD Biosciences; version 10.8.1); Figure composition: Prism software (GraphPad; version 9.2.0) and Illustrator CS4 (Adobe; version 14.0.0). No new custom software or code has been used in this paper. Codes (R programming language) used for bioinformatic analyses are all strictly based on pre-existing, regularly used published codes for RNA-Seq and single cell RNA-Seq analyses. These codes are available at https://github.com/apcamargo/2020-pths-rna-seq-analysis (Zenodo repository DOI: doi.org/10.5281/zenodo.6323774), along with examples of their use with our dataset and validation data, to ensure reproducibility.

For manuscripts utilizing custom algorithms or software that are central to the research but not yet described in published literature, software must be made available to editors and reviewers. We strongly encourage code deposition in a community repository (e.g. GitHub). See the Nature Research guidelines for submitting code & software for further information.

Data

Policy information about availability of data

All manuscripts must include a data availability statement. This statement should provide the following information, where applicable:

- Accession codes, unique identifiers, or web links for publicly available datasets
- A list of figures that have associated raw data
- A description of any restrictions on data availability

Data supporting the findings in this study are included within the Supplementary Material. The source data relevant to Figs. 1–9 and Supplementary Figs. 1–13 are provided as a Source Data file. RNA sequencing and single cell RNA sequencing raw and processed data generated during this study were deposited at the Gene Expression Omnibus (GEO) of the National Center for Biotechnology Information (NCBI), under accession numbers GSE159392 (https://www.ncbi.nlm.nih.gov/geo/query/acc.cgi?acc=GSE159392), GSE159859 (https://www.ncbi.nlm.nih.gov/geo/query/acc.cgi?acc=GSE159859), GSE159860 (https://www.ncbi.nlm.nih.gov/geo/query/acc.cgi?acc=GSE159860), and GSE189121 (https://www.ncbi.nlm.nih.gov/geo/query/acc.cgi?acc=GSE189121), which are available publicly without restriction. The following public databases have been used in this study and can be accessed via the corresponding weblinks in parentheses: GRCh38-2020-A (10x) (support.10xgenomics.com/single-cell-gene-expression/software/release-notes/build#GRCh38_2020A); GENCODE release 32 (www.gencodegenes.org/human/release_32.html); and GENCODE release 34 (www.gencodegenes.org/human/release_34.html). Large tables containing data related to gene expression and differential expression analyses of bulk RNA sequencing experiments and data related to differential expression analysis of single cell RNA sequencing experiments (Supplementary Data 2 and 3) are publicly available without restriction from the Zenodo repository (Zenodo repository DOI: doi.org/10.5281/zenodo.6325406). Microscopy images obtained during this study were not deposited in public repositories as they contain human patient sensitive information, but requests for these data will be fulfilled by the corresponding authors upon reasonable request following appropriate procedures of the Ethics Committees of the institutions where the patient biological samples and cells were collected or are maintained. Source data are provided with this paper.

Further information and requests for resources and reagents should be directed to and will be fulfilled by the corresponding authors, Fabio Papes (fpapes@ucsd.edu; papesf@unicamp.br) and Alysson Muotri (muotri@ucsd.edu), upon reasonable request. When provided to others, unique reagents generated in this study will be available with a completed Materials Transfer Agreement.

Field-specific reporting

Please select the one below that is the best fit for your research. If you are not sure, read the appropriate sections before making your selection.

[x] Life sciences [ ] Behavioural & social sciences [ ] Ecological, evolutionary & environmental sciences

For a reference copy of the document with all sections, see nature.com/documents/nr-reporting-summary-flat.pdf

Life sciences study design

All studies must disclose on these points even when the disclosure is negative.

Sample size	Sample sizes are indicated in the figure legends and in Supplementary Data 1 file. We did not use power analysis to determine a priori sample sizes, because we were initially restricted by the patient samples available, which were chosen based on availability of detailed information about the types of TCF4 mutation carried by each patient. However, based on the strong and consistent effect sizes observed throughout the study (Supplementary Data 1 file) and on the level of variability across cell lines from all subjects (in NPCs and organoids), further power analysis determined that increasing sample size is not expected to change statistical significance of our results. Power analysis was performed using G*Power aiming at a target power of 0.9 (90% of probability of rejecting the null hypothesis when it is in fact false, that is, of avoiding a type II error), with an alpha of 0.05 (probability of type I error) and considered two-tailed tests for comparison between independent groups.
Data exclusions	The only data exclusion applied throughout the study was for rare instances when technical replicates in qPCR experiments were clear outliers. We used G*power software and R (Outliers package) to determine effect sizes and outliers in this case.
Replication	Most experiments in this study were conducted with 4 or 5 subjects (patients and controls) per group. For every subject, most experiments were conducted with at least 3 independent batches, which were considered independent biological replicates for each subject in figures throughout the study and in Supplementary Data 1, with at least 3 technical replicates (wells of organoids or cells) per batch. For phenotypic evaluations conducted on 4 or more separate batches, we used two or more independent clones of iPSCs to produce organoids and NPCs to confirm the effect of genotype, as depicted in Supplementary Fig. 2a. When experimentation involved more than one independent replicate per subject cell line, or more than one technical replicate per independent replicate, the numbers of replicates are also indicated in the figure legends and Supplementary Data 1, even though each statistical test was computed based solely on the comparison between the means of different subjects. All attempts at replication were successful. P-values are reported as asterisks in the figures for significance levels defined as $p<0.05$ (*), $p<0.01$ (**), or $p<0.001$ (***). Supplementary Data 1 file presents extended results for all statistical tests performed, including sample sizes, statistical tests employed, effect sizes, statistics metrics (H, F, t, or W), along with exact p-values, listed according to order of appearance in the figure panels throughout the study.
Randomization	Experiments conducted with 4 subjects always included samples from PTHS #1 to #4, and experiments conducted with 3 subjects always included samples from PTHS #1, #2 and #4, along with the respective parental controls, and no randomization was applied because we were restricted by the limited number of patients with PTHS included in the study and by the different types of TCF4 mutation each subject carries. When experiments involved data collected from independent biological replicates, results were collected from randomly chosen replicates (batches in organoid preparation or wells/plates of experiments involving cells in 2D culture). For each batch, organoids were randomly selected from each well for data collection.
Blinding	Blinding was used for most analyses comparing patients and control samples, including immunostaining, measurement of organoid size, cell counting, patch-clamp electrophysiological measurements, and multi-electrode array assays. Blinding was not used when analyzing results

from RNA sequencing and single cell RNA sequencing experiments, due to the inherently unbiased nature of the bioinformatic approaches used for quantitating gene expression and determining differential expression between genotypes or cell types.

Reporting for specific materials, systems and methods

We require information from authors about some types of materials, experimental systems and methods used in many studies. Here, indicate whether each material, system or method listed is relevant to your study. If you are not sure if a list item applies to your research, read the appropriate section before selecting a response.

Materials & experimental systems

n/a	Involved in the study	
☐	☒	Antibodies
☐	☒	Eukaryotic cell lines
☒	☐	Palaeontology and archaeology
☒	☐	Animals and other organisms
☐	☒	Human research participants
☒	☐	Clinical data
☒	☐	Dual use research of concern

Methods

n/a	Involved in the study	
☒	☐	ChIP-seq
☐	☒	Flow cytometry
☒	☐	MRI-based neuroimaging

Antibodies

Antibodies used

Antibodies used in this study were: rabbit anti-SOX2 (Abcam; ab97959; 1:1000); rabbit anti-OCT4 (Abcam; ab19857; 1:100); rabbit anti-NANOG (GeneTex; GTX100863; 1:100); rabbit anti-LIN28 (Cell Signaling Technology; 3978; 1:500), rat anti-CTIP2 (Abcam; ab18465; 1:500); rabbit anti-SATB2 (Abcam; ab34735; 1:200); chicken anti-MAP2 (Abcam; ab5392; 1:1000); rabbit anti-SOX2 (Cell Signaling Technology; 2748; 1:500); rabbit anti-GAD65/67 (Abcam; ab11070; 1:200); rabbit anti-CUX1 (CUTL1 or CASP) (Abcam; ab54583; 1:200); rabbit anti-TCF4 (Abcam; ab217668; 1:1000); rabbit anti-vGLUT1 (Synaptic Systems; 135311; 1:500); rabbit anti-CC3 (Cleaved Caspase 3) (Cell Signaling Technology; 9664S; 1:500); rabbit anti-doublecortin (DCX) (Abcam; ab18723; 1:200); mouse anti-Cas9 (Abcam; ab210571; clone [8C1-F10]; 1:200); mouse anti-p16INK4a (CDKN2A) (Abcam; ab54210; clone [2D9A12]; 1:1000); rabbit anti-SOX3 (Abcam; ab183606; 1:200); mouse anti-Nestin (Abcam; ab22035; clone [10C2]; 1:1000); goat anti-SOX17 (R&D Systems; AF1924; 1:200); rabbit anti-Brachyury (Sigma; B8436; 1:200); rabbit anti-beta-catenin (Cell Signaling Technology; 9582S; 1:100); rabbit anti-FOXG1 (Abcam; ab196868; 1:500); mouse anti-AP2 (TFAP2A) (Thermo Fisher; MA1-872; clone [3B5]; 1:50); rabbit anti-HOPX (Abcam; ab230544; 1:100); rabbit anti-c-Fos (EMD Millipore; PC38, 1:1000); mouse anti-beta-actin (Abcam; ab6276; clone [AC-15]; 1:10,000); chicken anti-vimentin (VIM) (Abcam; ab22651; 1:2000); anti-rabbit Alexa-488 secondary antibody (Thermo Fisher; A-11034; 1:500); IRDye 680RD goat anti-rabbit IgG (H + L) (Li-COR; #926-68071; 1:5,000); or IRDye 800CW donkey anti-mouse IgG (H + L) (Li-COR; #926-32212; 1:5,000).

Validation

All antibodies employed in this study are commercially available antibodies commonly used is several other publications. The anti-TCF4 antibody was validated via Western Blotting on protein extracts from cell lines over-expressing the human TCF4 coding sequence. All other antibodies were validated in the lab to exclude non-specific signals due to secondary antibodies. Additionally, the primary antibodies were validated for immunofluorescence experiments by the manufacturers, as follows. The specificity of antibodies from Abcam was confirmed by the manufacturer via analyzing cells that either do or do not express the target protein within the same tissue and in different tissues, using multi-normal human tissue microarrays (TMAs), resulting in the analysis of many tissues at the same time, providing uniformity as all tissues are exposed to the exact same conditions, as stated on their website (https://www.abcam.com/primary-antibodies/how-we-validate-our-antibodies). Similar orthogonal validation was used by GeneTex for the anti-NANOG antibody. Antibodies from Cell Signaling Technology were validated by the manufacturer via a combination of analyzing cell lines or tissues with known target expression levels and performing siRNA treatment or over-expression of the target protein to verify target specificity, as specified on their website (https://www.cellsignal.com/about-us/our-approach-process/antibody-validation-immunofluorescence). The anti-vGLUT1 antibody from Synaptic Systems, the anti-SOX17 antibody from R&D Systems, and the anti-AP2 antibody from Thermo Fisher were validated by the manufacturers via gene knockdown. The anti-c-Fos antibody from EMD Millipore was validated in many studies and by our team using a combination of naturally transient gene expression after neuronal activation and a combination of immunostaining with in situ hybridization (DOI: 10.1016/j.xpro.2020.100153).

Eukaryotic cell lines

Policy information about cell lines

Cell line source(s)

iPSC cell lines were obtained via cellular reprogramming of patient or control fibroblasts. All iPSC clones were derived at the University of California San Diego or at the University of Campinas with IRB approval and written consent. HEK293T cells were obtained from ATCC (Cat# CRL-3216).

Authentication

All iPSC cell lines were assessed for pluripotency and genomic integrity. A total of 20 iPSC clonal lines were produced for each subject in the study, all of which were analyzed through a combination of immunostaining and SNP mapping to rule out the presence of unwanted chromosomal abnormalities and mutations (example in Supplementary Fig. 1c). The HEK293T cell line was authenticated via short tandem repeat profiling (performed every 5 months for passages 2, 5 and 10 of our original frozen stocks).

Mycoplasma contamination

Cultures were tested every two weeks for mycoplasma, and contamination was never identified at any stage.

Commonly misidentified lines (See ICLAC register)	No misidentified cell lines were used.

Human research participants

Policy information about studies involving human research participants

Population characteristics	To maximize comparability, we selected only male patients with PTHS for the histological and manipulative experiments in this study, and they are 4 to 14 years old (patients #1 to #5). Control subjects were the patients' corresponding fathers (30 to 50 years old), who had no history of psychiatric or genetic disorders. The post-mortem PTHS brain cortex sample is from a female individual who died during a surgical procedure to correct scoliosis, due to complications unrelated to the PTHS neurological symptoms (patient #6). A detailed and personalized questionnaire to gather information related to the patients' PTHS clinical symptoms was answered by all participating families, encompassing questions about neurological findings, cognitive, behavioral and gastroenterological manifestations, age at diagnosis, general quality of life, temporal evolution of motor milestones, communication level, dysmorphic facial features, urological symptoms, vision problems, sensory responsivity, sleep disturbances, respiratory anomalies such as apnea and hyperventilation, feeding habits and bowel symptoms, history of seizures, as well as MRI findings. These data are also reported in Supplementary Table 1.
Recruitment	Patients were recruited either at Institute of Biology and Medical School hospital of the University of Campinas (Brazil) or at the University of California San Diego School of Medicine (USA). For the latter, patients were recruited with the intermediation of the Pitt Hopkins Research Foundation. Patients were recruited via direct contact with the parents, without any bias related to gender, age, or ethnical or economic background. Although self-bias is inherently present due to the geographical location of the participant families' place of abode, this was minimized by recruiting families from the whole country and abroad who either virtually or physically attended the Pitt Hopkins Annual Conferences in 2018, 2019 and 2020 (organized by the Pitt Hopkins Research Foundation) or who were registered at the international Pitt Hopkins family registry. Although PTHS is caused by de novo mutations in the TCF4 gene and is expected to be equally prevalent independent of geographic factors, it is possible that an inherent bias due to the location of our laboratories may have resulted in the selection of patients with a particular genetic background, and care should be exercised when extrapolating our findings to other backgrounds, a point that should be further explored in future studies.
Ethics oversight	Participation of all subjects was approved by the Human Subjects Ethics Committees of the institutes in which the study was conducted (University of California San Diego IRB/ESCRO Committee and University of Campinas Ethics on Human Subjects Committee). Written informed consent was obtained from all participating families after receiving a thorough description of the study and no compensation was provided to participants.

Note that full information on the approval of the study protocol must also be provided in the manuscript.

Flow Cytometry

Plots

Confirm that:

- [x] The axis labels state the marker and fluorochrome used (e.g. CD4-FITC).
- [x] The axis scales are clearly visible. Include numbers along axes only for bottom left plot of group (a 'group' is an analysis of identical markers).
- [x] All plots are contour plots with outliers or pseudocolor plots.
- [x] A numerical value for number of cells or percentage (with statistics) is provided.

Methodology

Sample preparation	For the experiment shown in Fig. 5f (EdU labeling of NPCs in 2D culture), we used the Click-iT EdU Flow Cytometry Assay Kit (Thermo Fisher Scientific), following the manufacturer's protocol. Briefly, 70% confluent NPCs from 10 cm dishes were dissociated with Accutase, resuspended in StemDiff Neural Progenitor Medium (Stem Cell Technologies) and plated onto Matrigel-coated 6-well plates at a density of 0.2 × 10^6 cells/well. Cells were incubated at 37°C and 5% CO2 for 12 h before EdU was added to the culture medium at a final concentration of 10 micromolar. Cells were incubated for another 2.5 h for EdU incorporation, and subsequently harvested by Accutase-mediated dissociation, resuspension in 3 mL of 1% BSA in 1× PBS, and pelleting at 500 × g for 5 min. Pellets were resuspended and incubated in the kit's fixative solution for 15 min in the dark at 25°C, followed by the addition of 3 mL of 1% BSA in 1× PBS to stop fixation. Next, NPCs were pelleted at 500 × g for 5 min, the supernatant was removed, and the pelleted cells were incubated for 15 min in 1× Click-iT saponin-based permeabilization and wash reagent. During incubation, the Click-iT reaction cocktail was prepared based on the manufacturer's protocol, and then added to the samples, followed by homogenization and incubation for 30 min, protected from light. Cells were re-homogenized every 5 min and then washed in 3 mL of 1× Click-iT permeabilization and wash reagent, pelleted, and resuspended in the same solution, before nuclear staining in 1× PBS / 0.1% Triton X-100 / 100 micrograms/mL RNase A solution containing 20 micrograms/mL propidium iodide.<br><br>For the experiment shown in Supplementary Fig. 9g (flow cytometry-based determination of TCF4 protein expression levels), NPCs in 2D culture (3 replicate wells per subject/condition) were seeded onto 6-well plates at equivalent numbers and treated with DMSO (control) or CHIR99201 for 10 days. Cells were then washed with cold PBS, dissociated with Accutase for 5 min, followed by inactivation with 5 volumes of DMEM/F12 and centrifugation to pellet the cells. The NPCs were then subjected to incubation for 20 min with the LIVE/DEAD Fixable Dead Cell Stain 367/526 nm kit (Thermo Fisher Scientific; cat# L34965) to stain dead cells, following the manufacturer's recommendations. Immediately after, cells were fixed with 0.5% formaldehyde in 1× PBS for 15 min, pelleted, and permeabilized with 0.1% Triton X-100 in 1× PBS for 15 min. After

	centrifugation, cells were resuspended in 0.1% Triton X-100 / 1× PBS / 2% BSA and incubated for 30 min for blocking. Cells were then incubated with anti-TCF4 primary antibody (Abcam; ab217668; diluted 1:100 in the same solution) for 1 h, with constant flicking. After 3 washes in 0.1% Triton X-100 in 1× PBS, cells were centrifuged and incubated with 0.1% Triton X-100 / 1× PBS / 2% BSA containing anti-rabbit Alexa-488 secondary antibody (Thermo Fisher; 1:500) for 30 min in the dark. After 3 washes in 0.1% Triton X-100 in 1× PBS, cells were centrifuged and resuspended in ice-cold PBS and kept on ice in the dark until flow cytometry analysis on a LSR Fortessa X-20 cell cytometer (BD Biosciences).
Instrument	LSR Fortessa X-20 cell cytometer (BD Biosciences)
Software	Data were analyzed with the FlowJo v10.8.1 software (FlowJo, LLC).
Cell population abundance	No collection of sorted cells was applied in our study. Pre-sort fractions were typically at the concentration of 1 x 10^6 cells/ mL.
Gating strategy	For EdU assays, cell events in a SSC-A versus FSC-A plot were gated to include events between 0 and 150,000 in the SSC-A dimension and between 20,000 and 220,000 in the FSC-A dimension; this population was further gated to exclude doublets and clumps by including events below 100,000 in the FSC-W dimension and then events below 120,000 in the SSC-W dimension. The final population was analyzed for Alexa Fluor 647 fluorescence (EdU labeling) and propidium iodide fluorescence with excitation at 482 nm to reveal DNA content. The EdU+ population was defined in a parental control sample and the same gates were used for all remaining samples under analysis. The EdU+ population corresponds to the S cell cycle population and was gated according to standard practice, following the manufacturer's instructions, by selecting the arch of cells above 1,000 in the APC-A (650 nm excitation) dimension. The effectiveness of the gating strategy was confirmed with negative controls not labeled with EdU, not labeled with propidium iodide, or not labeled with both (Supplementary Fig. 14a,b).<br><br>For the flow cytometry determination of TCF4 protein expression levels, the following gating strategy was applied: we kept cell events below 1,000 in the BV510 dimension in a plot of LIVE/DEAD Fixable Dead Cell Stain kit fluorescence versus FSC-A, as we judged them to represent live cells, as per the kit's recommendations; this population was further gated in a SSC-A versus FSC-A plot to include events between 0 and 100,000-150,000 in the SSC-A dimension and between 20,000 and 260,000 in the FSC-A dimension; this population was further gated to exclude doublets and clumps by including events below 100,000 in the FSC-W dimension and then events below 110,000 in the SSC-W dimension. The final population was analyzed for Alexa Fluor 488 fluorescence (TCF4 labeling) with excitation at 515 nm to reveal TCF4 expression levels. The TCF4+ population was defined in a parental control sample and the same gates were used for all remaining samples under analysis. The TCF4+ population was defined as cells containing fluorescence above 125.00 in the Alexa Fluor 488 dimension. The effectiveness of this gating strategy was confirmed with negative controls not labeled with anti-TCF4 primary antibody or not labeled with both primary and secondary antibodies (Supplementary Fig. 14c,d).

☒ Tick this box to confirm that a figure exemplifying the gating strategy is provided in the Supplementary Information.
